# Supplementary material for: SLIT3 fragments orchestrate neurovascular expansion and thermogenesis in brown adipose tissue
Source: Nat Commun. 2026 Mar 25;17:2445. doi: 10.1038/s41467-026-70310-9 (PMC13018599; doi:10.1038/s41467-026-70310-9)
Supplement: Supplementary file 1 — Supplementary Information [file 41467_2026_70310_MOESM1_ESM.pdf]

SLIT3 Fragments Orchestrate Neurovascular Expansion and Thermogenesis in Brown Adipose Tissue

Supplementary Information

This Supplementary Information file contains the following materials:

Supplementary Figures 1-13

Supplementary Tables 1-3

Uncropped scans of all blots and gels in Supplementary Figures

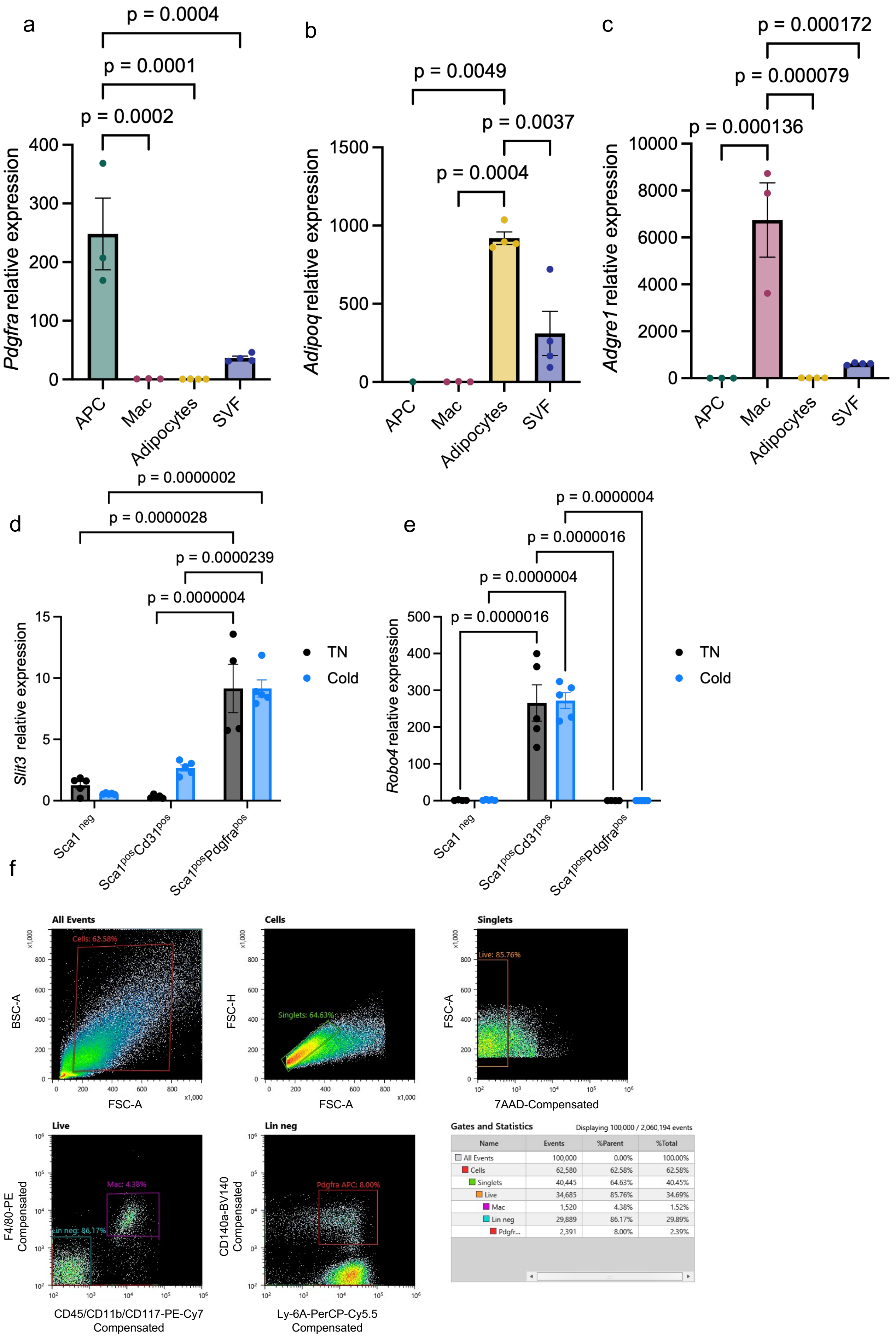

a
p = 0.0004
p = 0.0001
p = 0.0002
Pdgfra relative expression
APC
Mac
Adipocytes
SVF
b
p = 0.0049
p = 0.0037
p = 0.0004
Adipoq relative expression
c
p = 0.000172
p = 0.000079
p = 0.000136
Adgre1 relative expression
d
p = 0.0000002
p = 0.0000028
p = 0.0000239
p = 0.0000004
Slit3 relative expression
TN
Cold
Sca1 neg
Sca1posCd31pos
Sca1posPdgfrapos
e
p = 0.0000004
p = 0.0000016
p = 0.0000004
p = 0.0000016
Robo4 relative expression
f
All Events
Cells: 62.58%
BSC-A
FSC-A
Cells
Singlets: 64.63%
FSC-H
Singlets
Live: 85.76%
7AAD-Compensated
Live
Mac: 4.38%
Lin neg: 86.17%
F4/80-PE Compensated
CD45/CD11b/CD117-PE-Cy7 Compensated
Lin neg
Pdgfra APC: 8.00%
CD140a-BV140 Compensated
Ly-6A-PerCP-Cy5.5 Compensated
Gates and Statistics
Displaying 100,000 / 2,060,194 events
Name | Events | %Parent | %Total
All Events | 100,000 | 0.00% | 100.00%
Cells | 62,580 | 62.58% | 62.58%
Singlets | 40,445 | 64.63% | 40.45%
Live | 34,685 | 85.76% | 34.69%
Mac | 1,520 | 4.38% | 1.52%
Lin neg | 29,889 | 86.17% | 29.89%
Pdgfr... | 2,391 | 8.00% | 2.39%

Supplementary Figure 1. Related to Figure 1.

(a-c) Expression of *Pdgfra*, *Adipoq*, and *Adgre1* in isolated adipocyte progenitors, macrophages, adipocytes, and total stromal vascular fraction (SVF) from mouse BAT. n = 3, 3, 4, and 4 samples. Each sample was derived from a pool of three animals.

(d-e) Expression of *Slit3* and *Robo4* in Sca1$^-$, Sca1$^+$Cd31$^+$, and Sca1$^+$Pdgfra$^+$ cells isolated from BAT of mice housed at thermoneutrality (30 °C) or cold (5 °C) for 7 days. n = 5 samples per group. Each sample was derived from a pool of three animals.

(f) Gating strategy for sorting macrophages and *Pdgfra*-expressing adipocyte progenitors from BAT SVF.

Source data are provided in the Source Data file. Data are presented as mean ± SEM and analyzed by one-way ANOVA with Dunnett's multiple comparisons test (a-c) and two-way ANOVA with Tukey's multiple comparisons test (d-e).

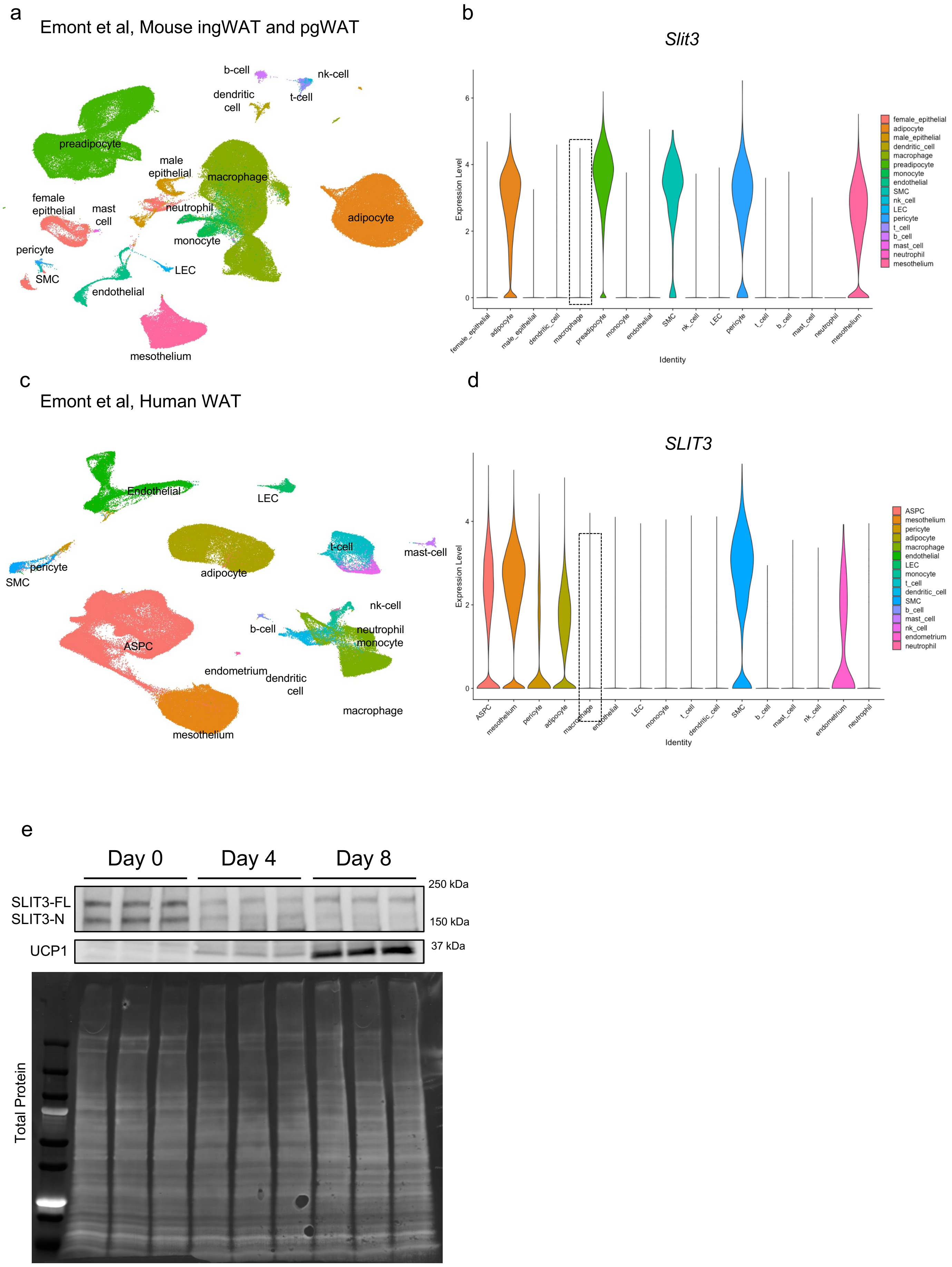
a
Emont et al, Mouse ingWAT and pgWAT
b-cell
nk-cell
dendritic cell
t-cell
preadipocyte
male epithelial
macrophage
female epithelial
mast cell
neutrophil
adipocyte
monocyte
pericyte
LEC
SMC
endothelial
mesothelium
b
Slit3
Expression Level
Identity
female_epithelial
adipocyte
male_epithelial
dendritic_cell
macrophage
preadipocyte
monocyte
endothelial
SMC
nk_cell
LEC
pericyte
t_cell
b_cell
mast_cell
neutrophil
mesothelium
c
Emont et al, Human WAT
Endothelial
LEC
pericyte
SMC
adipocyte
t-cell
mast-cell
nk-cell
b-cell
neutrophil
monocyte
ASPC
endometrium
dendritic cell
macrophage
mesothelium
d
SLIT3
Expression Level
Identity
ASPC
mesothelium
pericyte
adipocyte
macrophage
endothelial
LEC
monocyte
t_cell
dendritic_cell
SMC
b_cell
mast_cell
nk_cell
endometrium
neutrophil
e
Day 0
Day 4
Day 8
SLIT3-FL
SLIT3-N
250 kDa
150 kDa
UCP1
37 kDa
Total Protein

Supplementary Figure 2. Related to Figure 1.

(a-b) UMAP of unsupervised clustering of nuclei from mouse ingWAT and pgWAT (a) and violin plot showing *Slit3* transcript expression across clusters (b).

(c-d) UMAP of unsupervised clustering of nuclei from human WAT (c) and violin plot showing *SLIT3* transcript expression across clusters (d).

(e) SLIT3, UCP1, and total protein levels in SVF-derived brown adipocytes at day 0, day 4, and day 8 of differentiation. Source data are provided in the Source Data file. n = 3 samples per group.

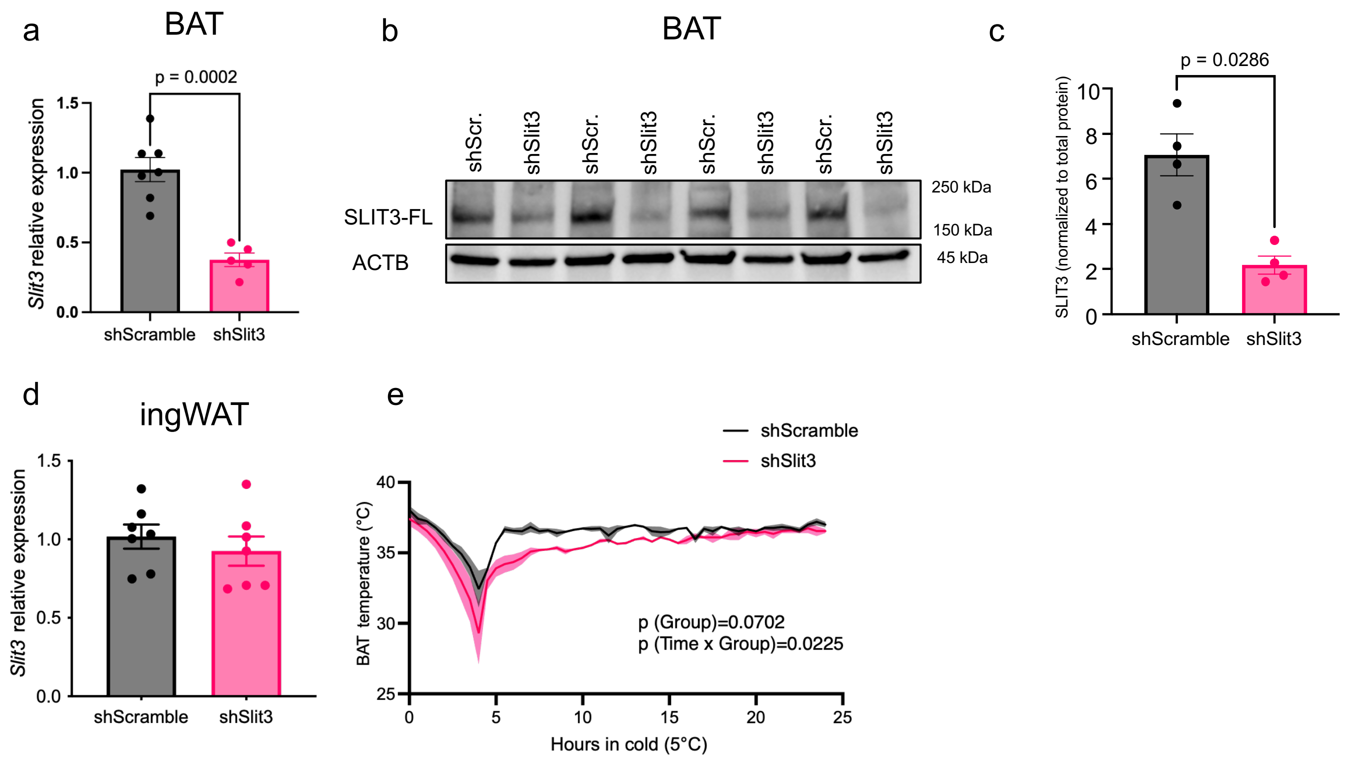

Supplementary Figure 3. Related to Figure 1.

(a) *Slit3* transcript levels in BAT. n= 7, 5 animals.

(b-c) SLIT3 protein levels in BAT. n= 4 animals per group.

(d) *Slit3* transcript levels in inguinal WAT (ingWAT). n= 7 animals per group.

(e) BAT temperature in mice injected with AAV-shSlit3 or scramble shRNA and housed at cold (5 °C) for 7 days. n= 4 animals per group

Data are presented as mean ± SEM and analyzed by unpaired two-sided Student's t-tests (a, c-d) and repeated measures ANOVA (e). Source data are provided in the Source Data file. The experiments were repeated two times with similar results.

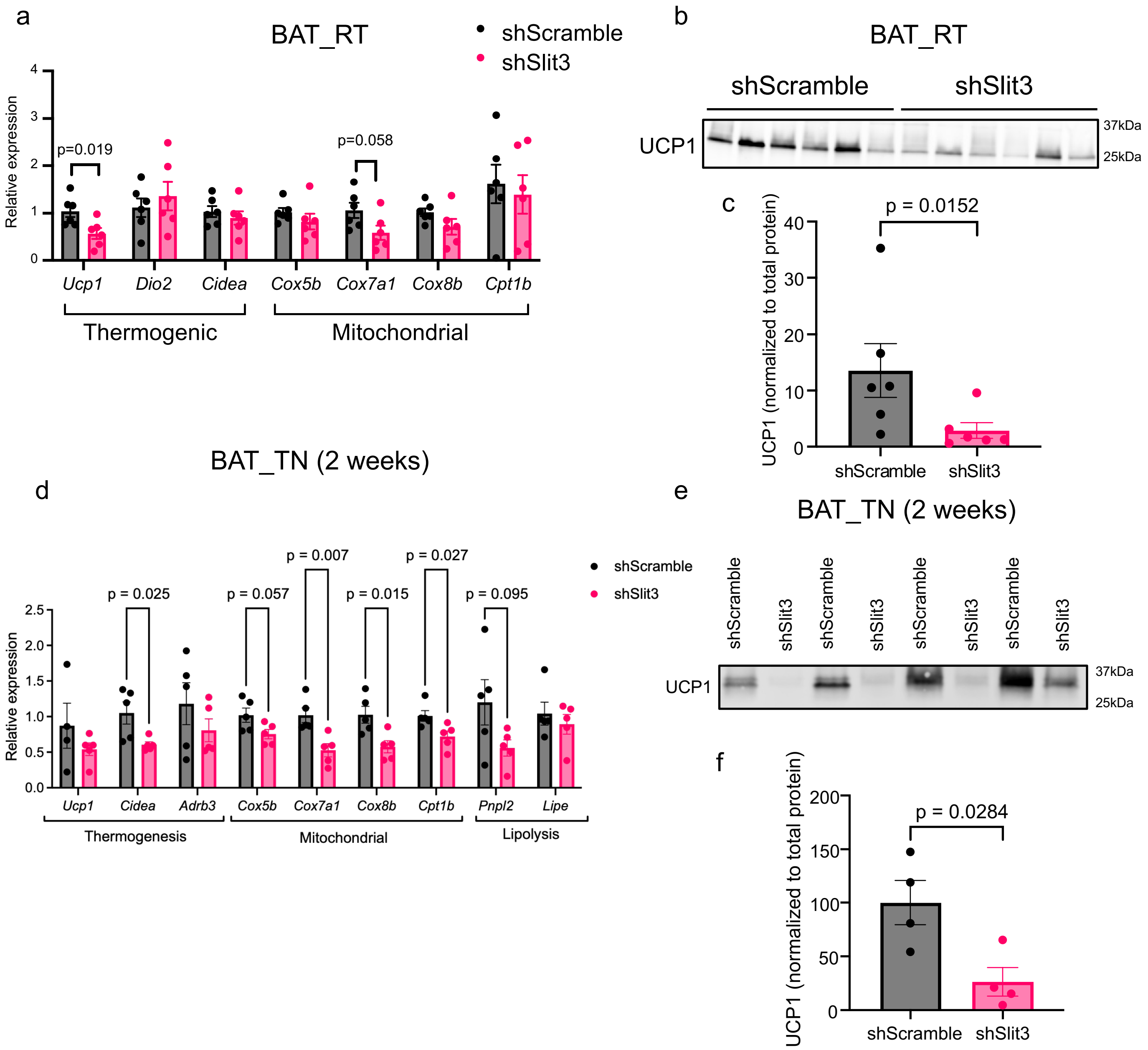

Supplementary Figure 4. Related to Figure 1.

(a) Expression of thermogenic and mitochondrial genes, and (b-c) UCP1 protein levels in BAT of mice injected with AAV-shSlit3 or scramble shRNA and housed at room temperature. n= 6 animals per group.

(d) Expression of thermogenic and mitochondrial genes. n= 6 animals per group, and (e-f) UCP1 protein levels in BAT of mice injected with AAV-shSlit3 or scramble shRNA and housed at thermoneutrality (30 °C) for two weeks. n= 4 animals per group.

Data are presented as mean ± SEM and analyzed by unpaired two-sided Student's t-test (a, c, d, f). Source data are provided in the Source Data file.

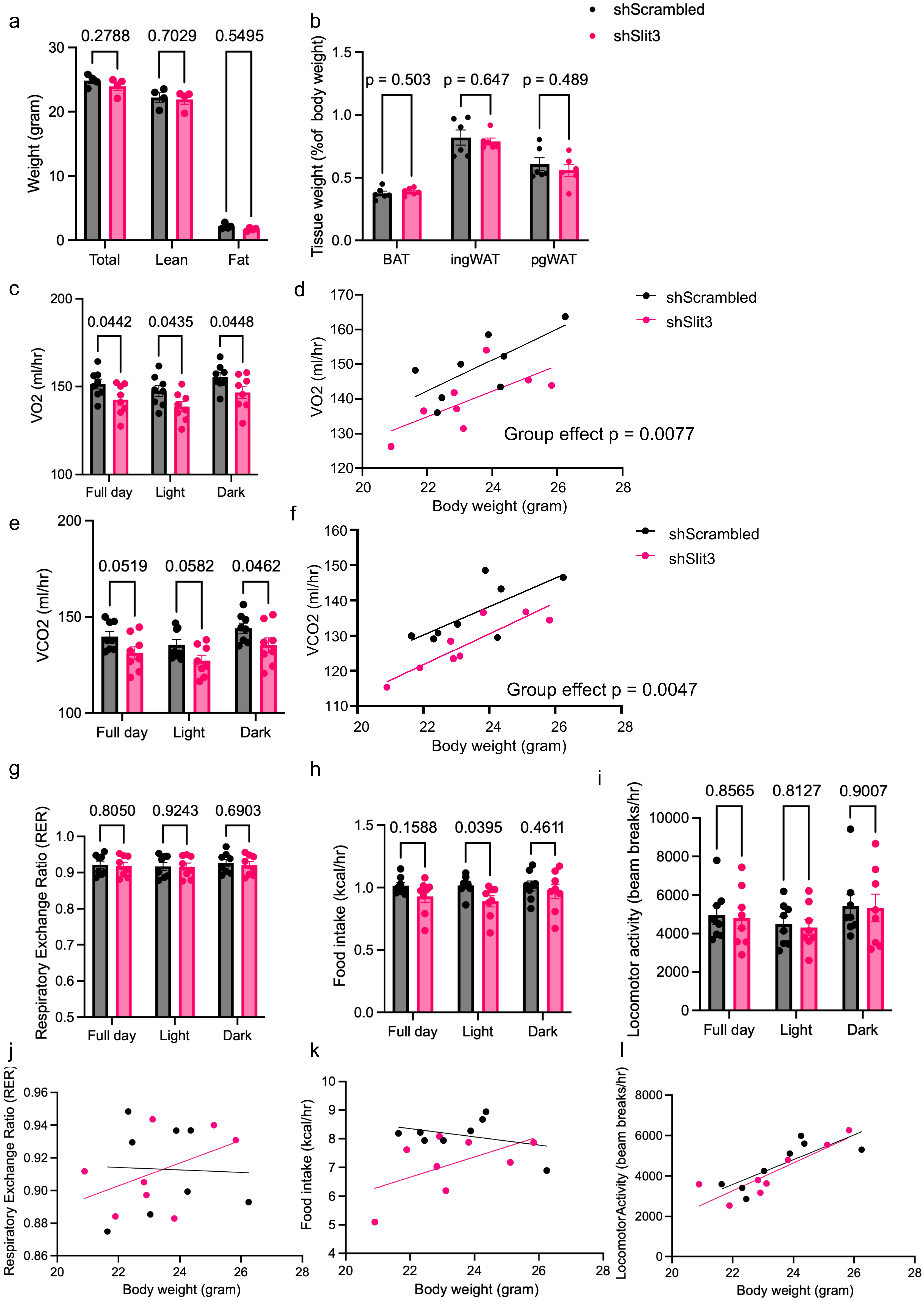
a
b
shScrambled
shSlit3
0.2788
0.7029
0.5495
Weight (gram)
Total
Lean
Fat
p = 0.503
p = 0.647
p = 0.489
Tissue weight (%of body weight)
BAT
ingWAT
pgWAT
c
0.0442
0.0435
0.0448
VO2 (ml/hr)
Full day
Light
Dark
d
VO2 (ml/hr)
Group effect p = 0.0077
Body weight (gram)
e
0.0519
0.0582
0.0462
VCO2 (ml/hr)
f
VCO2 (ml/hr)
Group effect p = 0.0047
Body weight (gram)
g
0.8050
0.9243
0.6903
Respiratory Exchange Ratio (RER)
h
0.1588
0.0395
0.4611
Food intake (kcal/hr)
i
0.8565
0.8127
0.9007
Locomotor activity (beam breaks/hr)
j
Respiratory Exchange Ratio (RER)
Body weight (gram)
k
Food intake (kcal/hr)
Body weight (gram)
l
LocomotorActivity (beam breaks/hr)
Body weight (gram)

Supplementary Figure 5. Related to Figure 1.

(a) Body composition analysis in mice injected with AAV-shSlit3 or scramble shRNA and housed at cold (5 °C) for 3 days. n= 4 animals per group.

(b) Adipose depot weights in mice injected with AAV-shSlit3 or scramble shRNA and housed at cold (5 °C) for 7 days. n= 6 animals per group.

(c, e, g-i) Average hourly rates of VO_2, VCO_2, respiratory exchange ratio (RER), food intake, and locomotor activity. n= 8 animals per group.

(d, f, j-l) Regression plots of VO_2 (c), VCO_2 (e), RER (i), food intake (j), and locomotor activity (k) versus total body mass. n= 8 animals per group.

Data are presented as mean ± SEM and analyzed by unpaired two-sided Student's t-test (a-b), two-way ANOVA (b, d, f-h), and ANCOVA (c, e). Source data are provided in the Source Data file. The experiment was repeated two times with similar results.

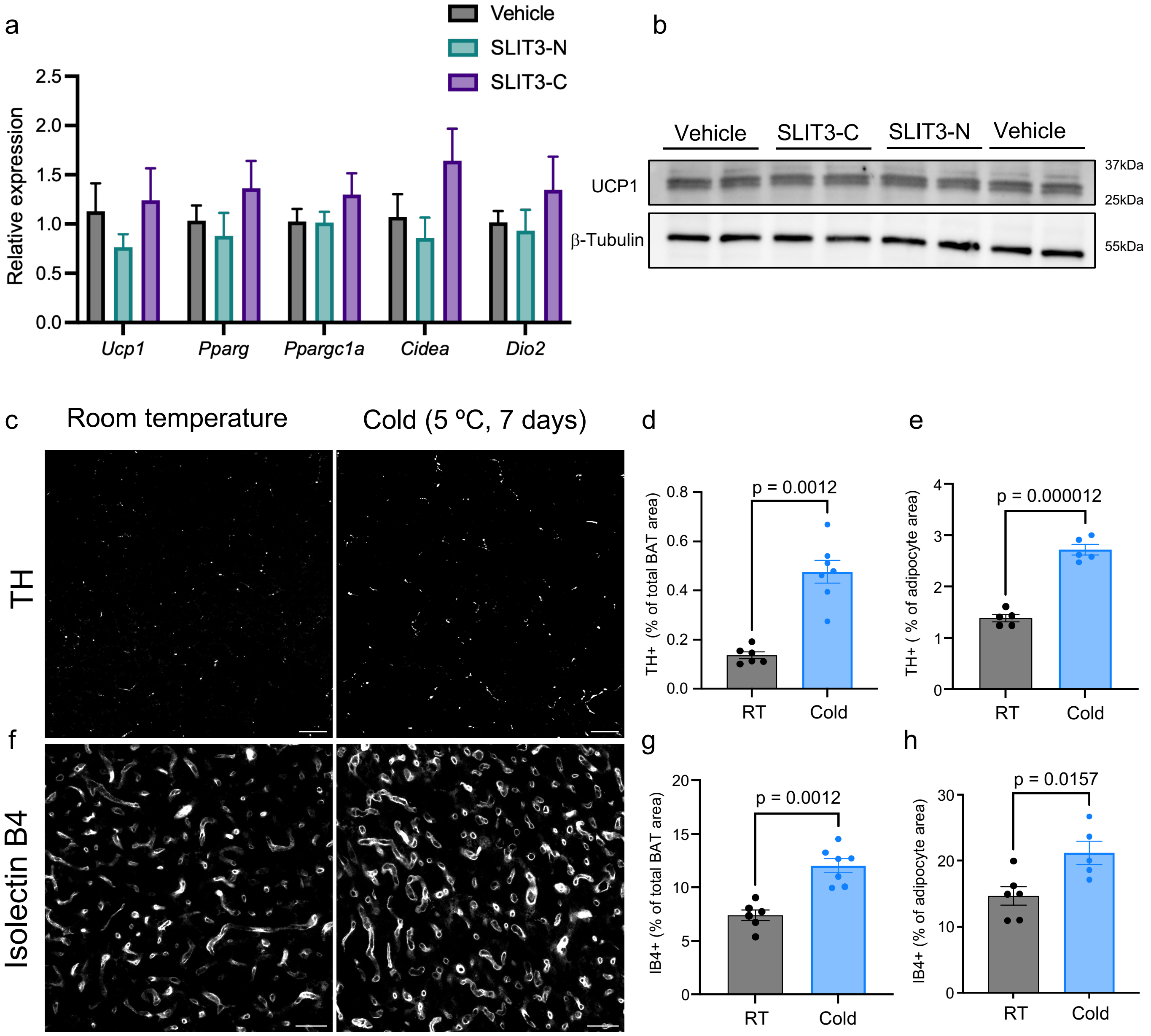

Supplementary Figure 6. Related to Figure 2.

(a) Expression of thermogenic genes in in vitro differentiated brown adipocytes treated with recombinant SLIT3-C (100 ng/ml), SLIT3-N (100 ng/ml), or vehicle for 48 hours.

(b) UCP1 protein levels in in vitro differentiated brown adipocytes treated with recombinant SLIT3-C, SLIT3-N, or vehicle for 48 hours.

(c) Representative images of TH and PLIN1 staining and (d-e) quantification of the percentage of TH^+ area normalized to the total tissue area (d) or to the $PLIN1^+$ area. n= 6, 7 animals. (e) in BAT of mice housed at room temperature and cold (5°C) for 7 days. n= 5 animals per group. Scale bar = 50 µm.

(f) Representative images of Isolectin B4 staining and (g-h) quantification of the percentage of IB4+ area normalized to the total tissue area (g) or to the $PLIN1^+$ area. n= 6, 7 animals. (h) in BAT housed at room temperature and cold (5°C) for 7 days. n= 6, 5 animals. Scale bar = 50 µm.

N = 5-7 per group. Data are presented as means ± SEM and analyzed by unpaired two-sided Student's t-tests (d-e and g-h). Source data are provided in the Source Data file. The experiments were repeated two times with similar results.

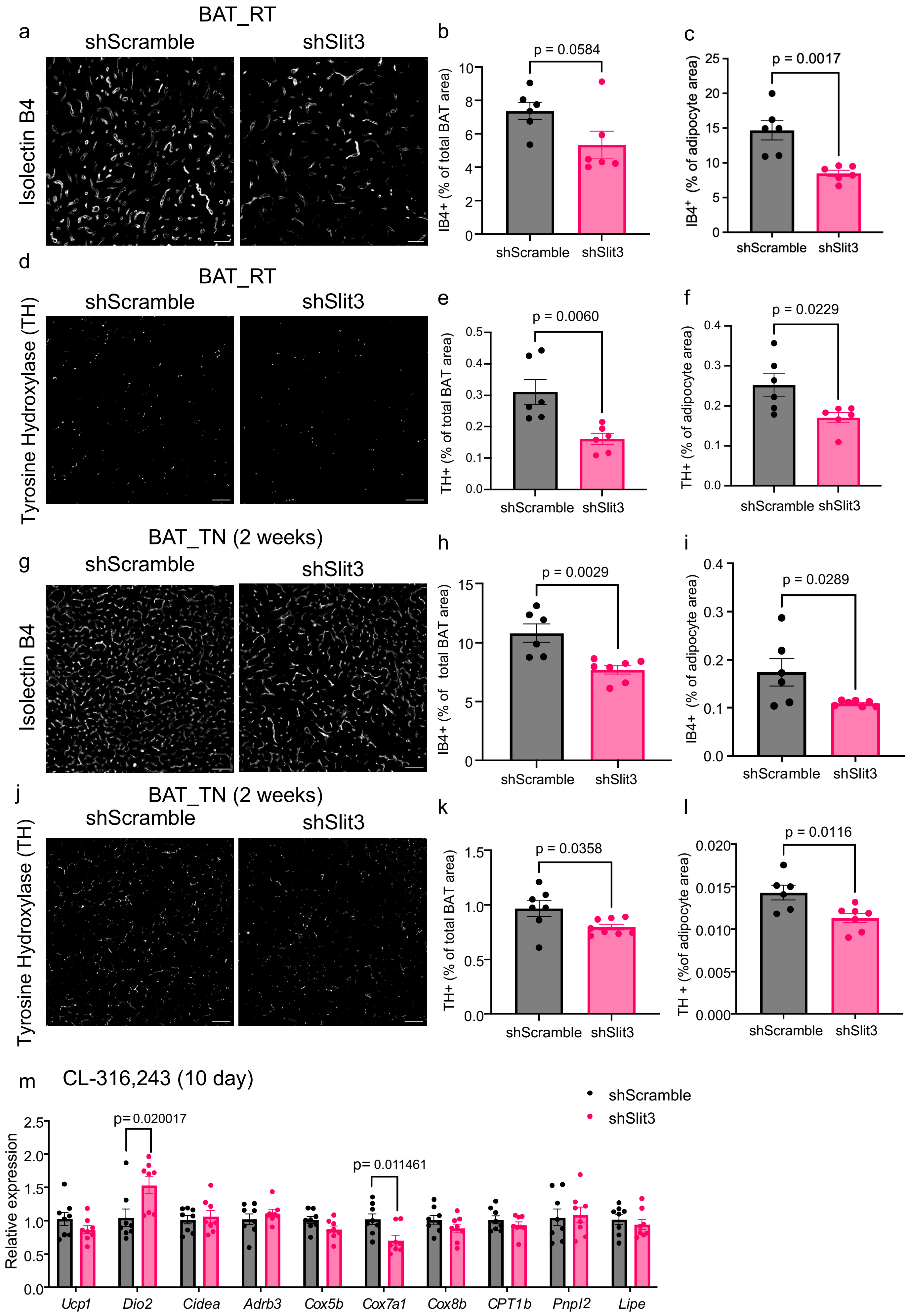

a
BAT_RT
shScramble
shSlit3
Isolectin B4
b
p = 0.0584
IB4+ (% of total BAT area)
shScramble
shSlit3
c
p = 0.0017
IB4+ (% of adipocyte area)
shScramble
shSlit3
d
BAT_RT
shScramble
shSlit3
Tyrosine Hydroxylase (TH)
e
p = 0.0060
TH+ (% of total BAT area)
shScramble
shSlit3
f
p = 0.0229
TH+ (% of adipocyte area)
shScramble shSlit3
g
BAT_TN (2 weeks)
shScramble
shSlit3
Isolectin B4
h
p = 0.0029
IB4+ (% of total BAT area)
shScramble
shSlit3
i
p = 0.0289
IB4+ (% of adipocyte area)
shScramble
shSlit3
j
BAT_TN (2 weeks)
shScramble
shSlit3
Tyrosine Hydroxylase (TH)
k
p = 0.0358
TH+ (% of total BAT area)
shScramble
shSlit3
l
p = 0.0116
TH + (%of adipocyte area)
shScramble
shSlit3
m
CL-316,243 (10 day)
shScramble
shSlit3
p= 0.020017
p= 0.011461
Relative expression
Ucp1
Dio2
Cidea
Adrb3
Cox5b
Cox7a1
Cox8b
CPT1b
Pnpl2
Lipe

Supplementary Figure 7. Related to Figure 2.

(a) Representative images of Isolectin B4 staining and (b-c) quantification of the percentage of Isolectin B4+ area normalized to the total tissue area (b) or to the PLIN1$^+$ area (c) in BAT of mice receiving AAV-shSlit3 or scramble shRNA and housed at room temperature. Scale bar = 20 μm. n= 6 animals per group.

(d) Representative images of TH staining and (e-f) quantification of the percentage of TH$^+$ area normalized to the total tissue area (e) or to the PLIN1$^+$ area (f) in BAT of mice receiving AAV-shSlit3 or scramble shRNA and housed at room temperature. Scale bar = 25 μm. n= 6 animals per group.

(g) Representative images of Isolectin B4 staining and (h-i) quantification of the percentage of Isolectin B4+ area normalized to the total tissue area (h) or to the PLIN1$^+$ area (i) in BAT of mice receiving AAV-shSlit3 or scramble shRNA and housed at thermoneutrality (30°C) for two weeks. Scale bar = 50 μm. n= 6, 7 animals.

(j) Representative images of TH staining and (k-l) quantification of the percentage of TH$^+$ area normalized to the total tissue area (k) or to the PLIN1$^+$ area (l) in BAT of mice receiving AAV-shSlit3 or scramble shRNA and housed at thermoneutrality (30°C) for two weeks. Scale bar = 50 μm. n= 7, 8 animals.

(i) Expression of thermogenic, mitochondrial, and lipolysis related genes in BAT of mice receiving AAV-shSlit3 or scramble shRNA and housed at room temperature following 10 day of CL-316,243 administration. n= 8 animals per group.

Data are presented as means ± SEM and analyzed by unpaired two-sided Student's t-tests. Source data are provided in the Source Data file.

a

Total protein staining for Figure 3a

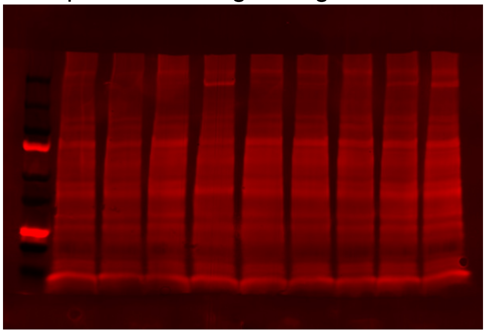

b

Total protein staining for Figure 3c

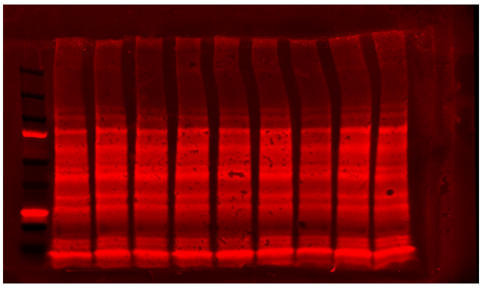

c

Total protein staining for Figure 3e

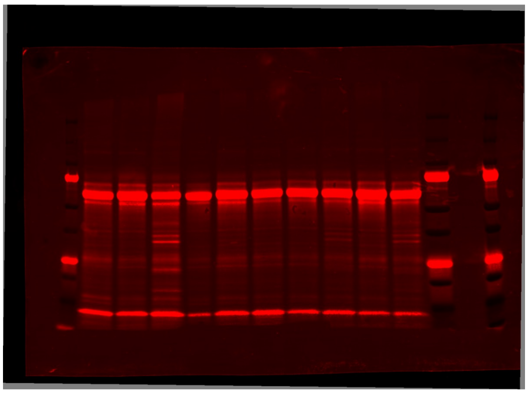

d

Total protein staining for Figure 3g

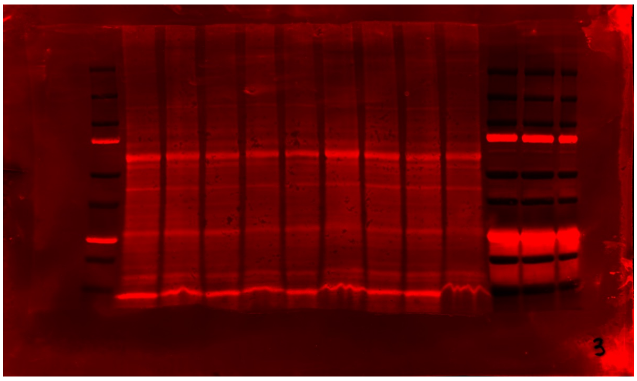

Supplementary Figure 8. Related to Figure 2.

(a-d) Total protein staining corresponding to Figures 3a, 3c, 3e, and 3g.

Tll1

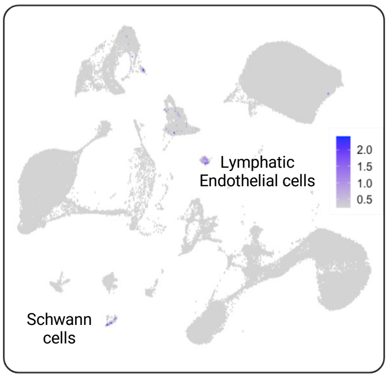

Supplementary Figure 9. Related to Figure 3.

Expression of *Tll1* in scRNA-seq data of mouse BAT.

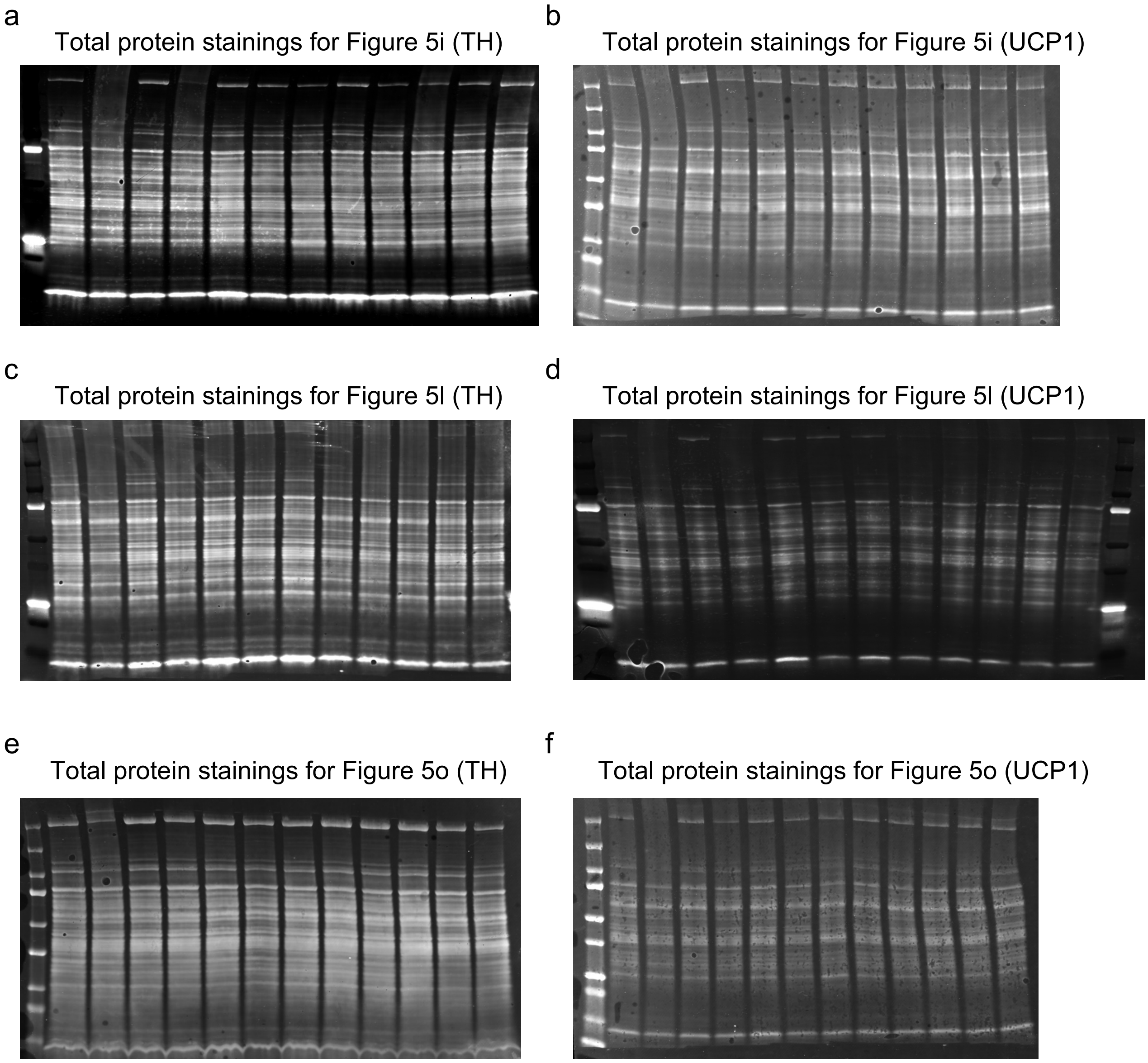

Supplementary Figure 10. Related to Figure 5.

(a-f) Total protein staining corresponding to Figures 5i, 5l, and 5o.

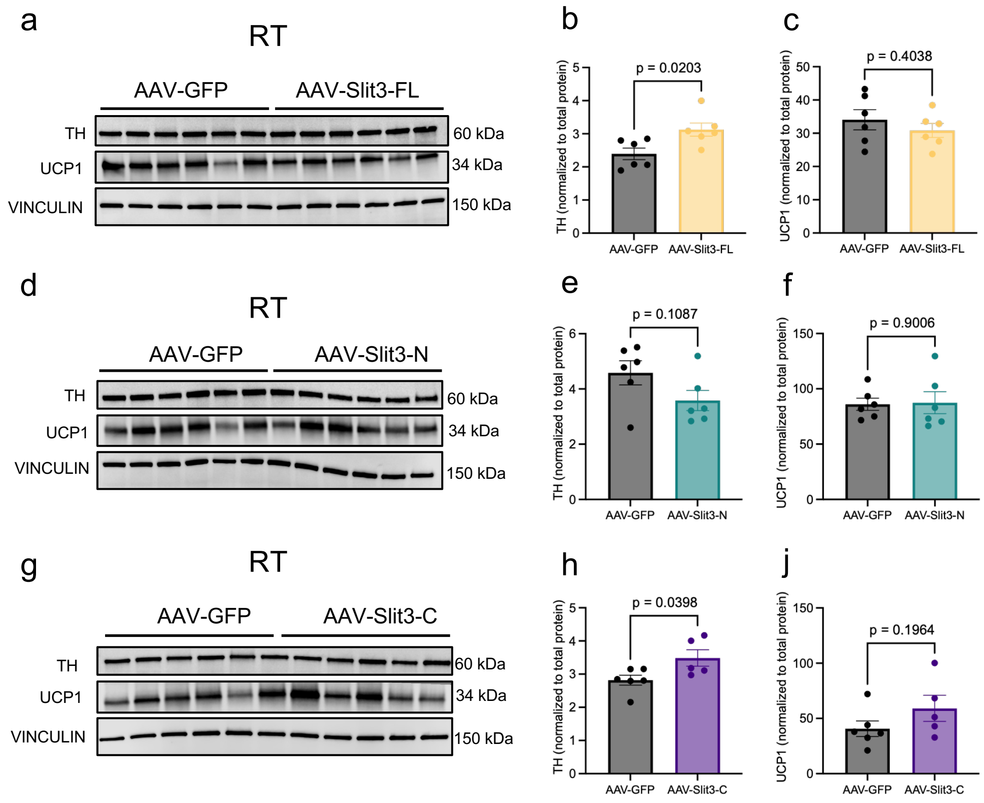

Supplementary Figure 11. Related to Figure 5.

(a-j) Western blots and quantification of TH and UCP1 in BAT from mice expressing AAV-GFP, AAV-Slit3-FL (a-c), AAV-Slit3-N (d-f), or AAV-Slit3-C (g-h) housed at room temperature. n= 6 animals per group.

Data are presented as mean ± SEM and analyzed by unpaired two-sided Student's t-test. Source data are provided in the Source Data file.

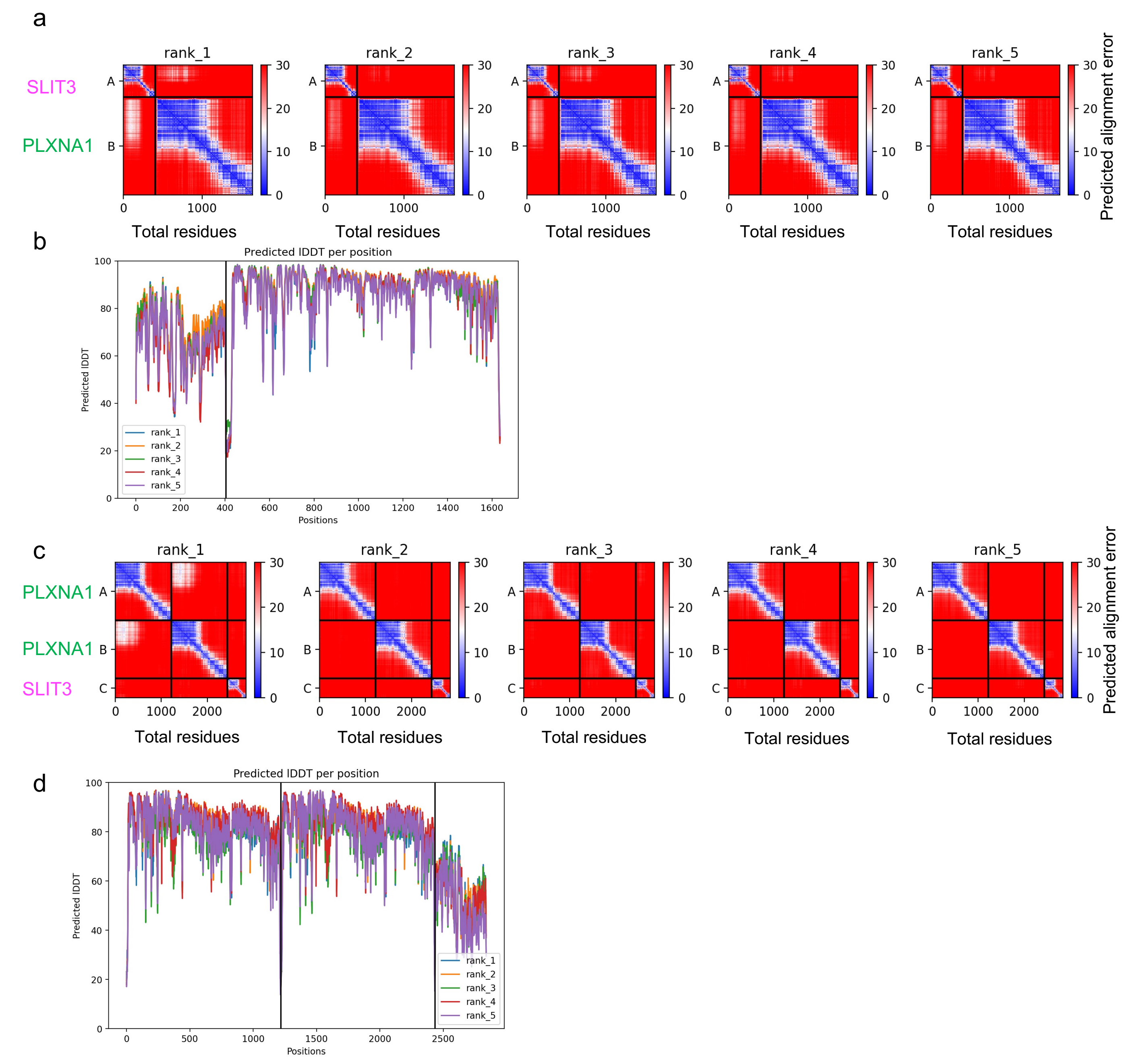

Supplementary Figure 12. Related to Figure 6.

AlphaFold2 Multimer predicts the human PLXNA1-SLIT3 complex. Diagnostic plots and images of 3D models demonstrate the quality of the protein complex predictions using the sequences of human PLXNA1 extracellular domains 1-10 and the C-terminal region of the human SLIT3.

(a) The PAE (predicted error in A° between every pair of residues) plots of monomeric PLXNA1 and SLIT3 interaction model. A and B tiles represent SLIT3 and PLXNA1, respectively. The X, Y axes indicate the total amino acids in these analyses.

(b) The pLDDT (predicted Local Distance Difference between all atoms) plots show the predicted IDDT per residue in the top five ranked models for monomeric PLXNA1- SLIT3 complex. The X, Y axes indicate the total amino acids in these analyses.

(c) The PAE plot of dimeric PLXNA1 and monomeric SLIT3 interaction model. A and B tiles represent each PLXNA1 monomer, and the C tile shows SLIT3.

(d) The pLDDT plots show the predicted lDDT per residue in the top five ranked models for the PLXNA1 dimer complex with SLIT3.

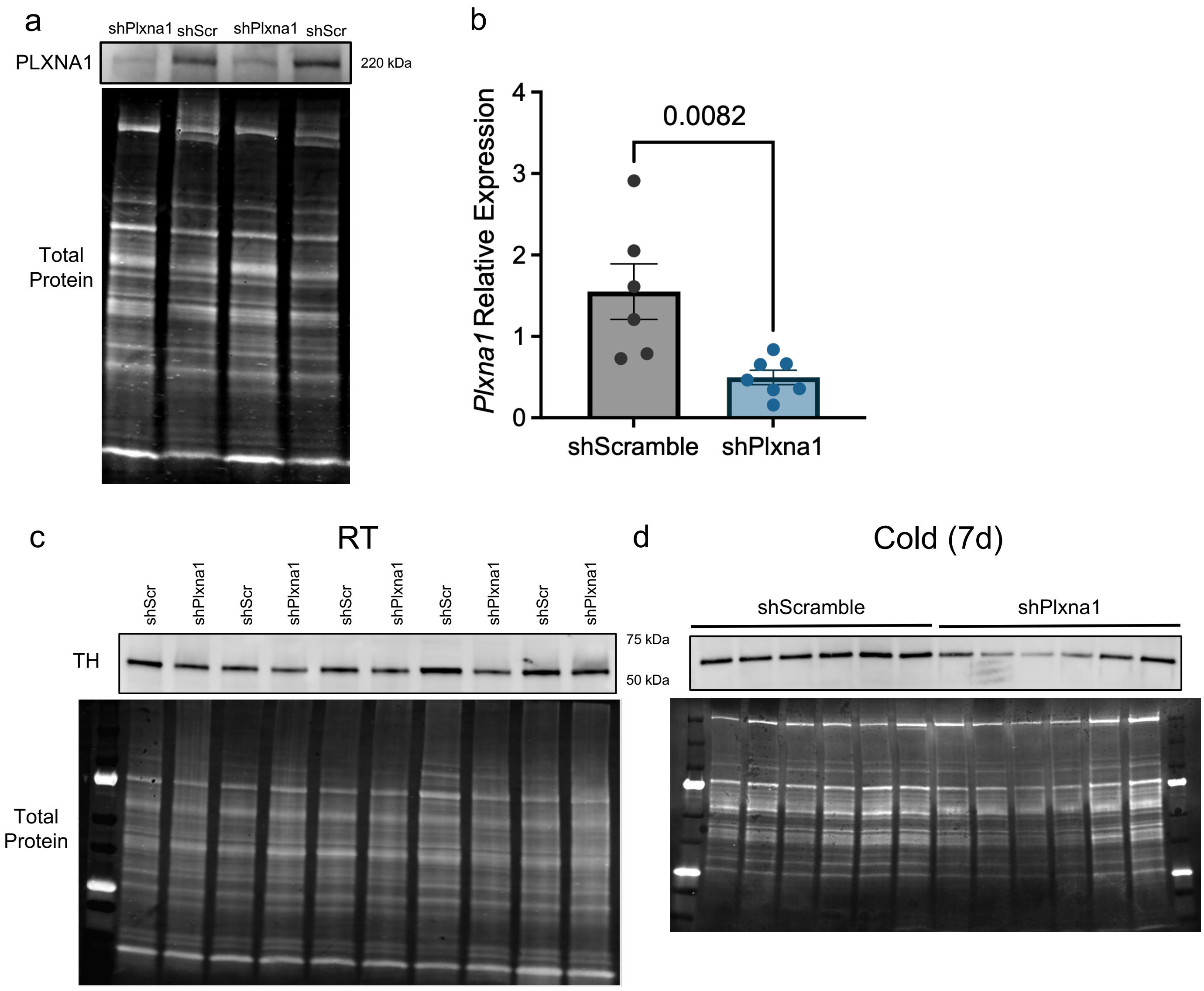

Supplementary Figure 13. Related to Figure 7.

(a) PLXNA1 protein and (b) transcript expression levels. n=6, 7 animals.

(c-d) Total TH protein levels in BAT of mice receiving AAV-shPlxna1 or scramble shRNA housed at (c) room temperature. n= 5 animals per groups. (d) cold for 7 days. n= 6 animals per group.

Data are presented as mean ± SEM and analyzed by unpaired two-sided Student's t-test (b). Source data are provided in the Source Data file. The experiment was repeated two times with similar results.

Supplementary Table 1. shRNA sequences.

Construct	Target Sequence	AAV serotype
AAV[shRNA]-mCherry-U6>mSlit3 [shRNA#1]	CGCTTGCTCATGCAGTAATAA	AAV8
pAAV[shRNA]-mCherry-U6>mSlit3 [shRNA#2]	GAACCTGCAACTGGACAATAA	AAV8
pAAV[shRNA]-mCherry-U6>mSlit3 [shRNA#3]	TTCGTGGGCAAGGACTCTTAT	AAV8
pAAV[shRNA]-mCherry-U6>Scramble shRNA	CCTAAGGTTAAGTCGCCCTCG	AAV8
pAAV[shRNA]-TagBFP2-U6>mPlxna1[shRNA#1]	TGGTTCTGCCCAGGGATATTT	AAV9
pAAV[shRNA]-TagBFP2-U6>mPlxna1[shRNA#2]	GGTTCTGCCCAGGGATATTTG	AAV9
pAAV[shRNA]-TagBFP2-U6>mPlxna1[shRNA#3]	CCGAGGTGAAGTACAACTATA	AAV9
pAAV[shRNA]-TagBFP2-U6>Scramble shRNA	CCTAAGGTTAAGTCGCCCTCG	AAV9

Supplementary Table 2. List of antibodies and dilutions used in this study.

**Antibody**	**Catalog number**	**WB**	**IHC**	**FACS**
Anti-Slit3 antibody	R&D Systems (AF3629)	1:1000		
Anti-Tyrosine Hydroxylase antibody	Millipore Sigma (AB1542)	1:1000	1:200	
Anti-UCP1 antibody	Abcam (Ab23841)	1:2000		
Anti-Total OXPHOS antibody	Abcam (Ab110413)	1:1000		
Anti- β Actin antibody	Cell Signaling (3700S)	1:1000		
Anti-Perilipin 1 antibody	Cell Signaling (9349S)		1:250	
Anti-Griifonia Simplicifolia Lectin I (GSL I) Isolectin B4, Biotynilated	Vector Laboratories (B-1205)		1:200	
Anti-Robo1 antibody	Abcam (ab7279)			
Anti-Robo4 antibody	Santa Cruz Biotech (sc-166872)			
Anti-Plexin A1 antibody	Abcam (ab23391)			
Anti-GAP43 antibody	Abcam (ab75810)		1:200	
Anti-SNAP-tag antibody	New England Biolabs (P9310S)	1:1000		
Anti-HaloTag antibody	Promega (G9211)	1:1000		
Anti-6X His tag® antibody [HIS.H8]	Abcam (ab18184)	1:5000		
HRP Goat-anti-mouse IgG secondary antibody	Invitrogen (31432)	1:5000		
HRP Donkey anti-goat IgG secondary antibody	Invitrogen (A16005)	1:5000		
HRP Donkey anti-rabbit IgG secondary antibody	Biolegend (406401)	1:5000		
HRP Donkey anti-Sheep IgG secondary antibody	Invitrogen (A16041)	1:5000		
Streptavidin (DyLight® 488) secondary antibody	Vector Laboratories (SA-5488-1)		1:200	

Alexa 594 Donkey anti-rabbit IgG secondary antibody	Biolegend (406418)		1:200	
Alexa 647 Donkey anti-Sheep IgG	Invitrogen (A21448)		1:200	
PE anti-mouse F4/80 Antibody	Biolegend (123110)			1:200
PE/Cyanine7 anti-mouse CD117 (c-Kit) Antibody	Biolegend (105814)			1:200
PE/Cyanine7 anti-mouse/human CD11b Antibody	Biolegend (101216)			1:200
PE/Cyanine7 anti-mouse CD45 Antibody	Biolegend (103114)			1:200
Alexa Fluor® 488 anti-mouse CD31 Antibody	Biolegend (102514)			1:200
Ly-6A/E (Sca-1) Monoclonal Antibody (D7), PerCP-Cyanine5.5	eBioscience (5015865)			1:200
Brilliant Violet 421™ anti-mouse CD140a Antibody	Biolegend (135923)			1:200
PE Rat IgG2a, κ Isotype Ctrl Antibody	Biolegend (400507)			1:200
PE/Cyanine7 Rat IgG2b, κ Isotype Ctrl Antibody	Biolegend (400617)			1:200
Alexa Fluor® 488 Rat IgG2a, κ Isotype Ctrl Antibody	Biolegend (400525)			1:200
Brilliant Violet 421™ Rat IgG2a, κ Isotype Ctrl Antibody	Biolegend (400535)			1:200

Supplementary Table 3. qPCR primer sequences.

Primer	Sequence (5'-3')
mSlit3-F2	ATGCCAACTCCCTCTCCTG
mSlit3-R2	CTAGGCGTATTTCCACGATGC
mSlit3-F3	AAGACTACGCCTCTTGTGCC
mSlit3-R3	GACATATTTCCGCCGCTTGC
mSlit3-F4	AGACAATGACCCCTTGGCAC
mSlit3-R4	TGGCCATCATTCACCGTCTC
mUcp1_F	CTGCCAGGACAGTACCCAAG
mUcp1_R	TCAGCTGTTCAAAGCACACA
mCidea_F	ATCACAACTGGCCTGGTTACG
mCidea_R	TACTACCCGGTGTCCATTTCT
mDio2_F	CAGTGTGGTGCACTGCTCCAATC
mDio2_R	TGAACCAAAGTTGACCACCAG
mEssra_F	GCAGGGCAGTGGGAAGCTA
mEssra_R	CCTCTTGAAGAAGGCTTTGCA
mCpt1b_F	CGAGGATTCTCTGGAACTGC
mCpt1b_R	GGTCGCTTCTTCAAGGTCTG
mCox5b_F	GCTGCATCTGTGAAGAGGACAAC
mCox5b_R	CAGCTTGTAATGGGTTCCACAGT
mCox7a1_F	CAGCGTCATGGTCAGTCTGT
mCox7a1_R	AGAAAACCGTGTGGCAGAGA
mCox8b_F	GAACCATGAAGCCAACGACT
mCox8b_R	GCGAAGTTCACAGTGGTTCC
mPparg_F	TCAGCTCTGTGGACCTCTCC
mPparg_R	ACCCTTGCATCCTTCACAAG

mPpargc1a_F	CCCTGCCATTGTTAAGACC
mPpargc1a_R	TGCTGCTGTTCCTGTTTTC
mAdiponectin_F	GGCAGGAAAGGAGAACCTGG
mAdiponectin_R	AGCCTTGTCCCTCTTGAAGAG
mFasn_F1	GGCTCTATGGATTACCCAAGC
mFasn_R1	CCAGTGTTCGTTCCTCGG
mLeptin_F1	CCTCATCAAGACCATTGTCACC
mLeptin_R1	TCTCCAGGTCATTGGCTATCTG
mVegfa_F	GCTTCCTACAGCACAGCAGA
mVegfa_R	AATGCTTTCTCCGCTCTGAA
mVegfr2 (Kdr)_F	TTT GGC AAA TAC AAC CCT TCA GA
mVegfr2 (Kdr)_R	GCA GAA GAT ACT GTC ACC ACC
mPecam1-F1	CTG CCA GTC CGA AAA TGG AAC
mPecam1-R1	CTT CAT CCA CCG GGG CTA TC
mRobo4-F1	ACTCGGGGACCTATATGTGTATG
mRobo4-R1	GGGATTCCTGGATAGACACCC
mAdrb3_F	GTCGTCTTCTGTGTAGCTACGGT
mAdrb3_R	CATAGCCATCAAACCTGTTGAG
mArbp_F	TTTGGGCATCACCACGAAAA
mArbp_R	GGACACCCTCCAGAAAGCGA
mTbp_F	ACG CTT CAC CAA TGA CTC CTA
mTbp_R	TGA CTG CAG CAA ATC GCT TGG
mPdgfra_F1	GACCTGCAGTGGACTTACCC
mPdgfra_R1	GGCAGCACATTCATACTCTCC
mPlxna1_F	GGGTGTGTGGATAGCCATCAG
mPlxna1_R	GCCAACATATACCTCTCCTGTCT
mAdgre1 (F4/80)_F	TTTCCTCGCCTGCTTCTTC
mAdgre1 (F4/80)_R	CCCCGTCTCTGTATTCAACC

mGap43-F	TGGTGTCAAGCCGGAAGATAA
mGap43-R	GCTGGTGCATCACCCTTCT
mCkb-F	GCC TCA CTC AGA TCG AAA CTC
mCkb-R	GGC ATG TGA GGA TGT AGC CC
