# Supplementary material for: SLIT3 fragments orchestrate neurovascular expansion and thermogenesis in brown adipose tissue
Source: Nat Commun. 2026 Mar 25;17:2445. doi: 10.1038/s41467-026-70310-9 (PMC13018599; doi:10.1038/s41467-026-70310-9)
Supplement: Supplementary file 2 — Reporting Summary [file 41467_2026_70310_MOESM2_ESM.pdf]

nature portfolio

Corresponding author(s): Farnaz Shamsi

Last updated by author(s): Jan 28, 2026

Reporting Summary

Nature Portfolio wishes to improve the reproducibility of the work that we publish. This form provides structure for consistency and transparency in reporting. For further information on Nature Portfolio policies, see our Editorial Policies and the Editorial Policy Checklist.

Statistics

For all statistical analyses, confirm that the following items are present in the figure legend, table legend, main text, or Methods section.

n/a	Confirmed	
☐	☒	The exact sample size (*n*) for each experimental group/condition, given as a discrete number and unit of measurement
☐	☒	A statement on whether measurements were taken from distinct samples or whether the same sample was measured repeatedly
☐	☒	The statistical test(s) used AND whether they are one- or two-sided<br>*Only common tests should be described solely by name; describe more complex techniques in the Methods section.*
☐	☒	A description of all covariates tested
☐	☒	A description of any assumptions or corrections, such as tests of normality and adjustment for multiple comparisons
☐	☒	A full description of the statistical parameters including central tendency (e.g. means) or other basic estimates (e.g. regression coefficient) AND variation (e.g. standard deviation) or associated estimates of uncertainty (e.g. confidence intervals)
☐	☒	For null hypothesis testing, the test statistic (e.g. *F*, *t*, *r*) with confidence intervals, effect sizes, degrees of freedom and *P* value noted<br>*Give P values as exact values whenever suitable.*
☒	☐	For Bayesian analysis, information on the choice of priors and Markov chain Monte Carlo settings
☒	☐	For hierarchical and complex designs, identification of the appropriate level for tests and full reporting of outcomes
☐	☒	Estimates of effect sizes (e.g. Cohen's *d*, Pearson's *r*), indicating how they were calculated

Our web collection on statistics for biologists contains articles on many of the points above.

Software and code

Policy information about availability of computer code

Data collection	Microscopy data were collected using Leica LAS X or Zeiss image acquisition software. Three-dimensional reconstructions were generated using Imaris (Bitplane). Western blot data were collected using Li-COR image acquisition software. Indirect calorimetry data were collected using TSE PhenoMaster software. Lean and fat mass were quantified using dual-energy X-ray absorptiometry (DEXA) (Insight, Osteosys). qPCR data were collected using the QuantStudio™ 5 Real-Time PCR System. FACS was performed using a Sony SH800 cell sorter. H&E-stained slides were imaged using an Aperio slide scanner (Leica Biosystems). Body temperature was measured using a rectal probe (Physitemp, RET3) with a reader (Physitemp, BAT-12), or using RFID transponder temperature microchips implanted under the interscapular BAT area (Unified Information Devices).
Data analysis	ImageJ was used for all image processing and quantifications. Western blot data are analyzed using LiCor Empiria Studio software. Indirect calorimetry data were analyzed using CalR.

For manuscripts utilizing custom algorithms or software that are central to the research but not yet described in published literature, software must be made available to editors and reviewers. We strongly encourage code deposition in a community repository (e.g. GitHub). See the Nature Portfolio guidelines for submitting code & software for further information.

Data

Policy information about availability of data

All manuscripts must include a data availability statement. This statement should provide the following information, where applicable:

- Accession codes, unique identifiers, or web links for publicly available datasets
- A description of any restrictions on data availability
- For clinical datasets or third party data, please ensure that the statement adheres to our policy

The data supporting the findings of this study are available within the paper and its supplementary information files. The human RNA-seq data from the LOBB have not been deposited in a public repository due to restrictions imposed by patient consent but can be obtained from Matthias Blüher upon request.

Research involving human participants, their data, or biological material

Policy information about studies with human participants or human data. See also policy information about sex, gender (identity/presentation), and sexual orientation and race, ethnicity and racism.

Reporting on sex and gender	The human data used in this research were sourced from the Leipzig Obesity Biobank (LOBB, https://www.helmholtz-munich.de/en/hi-mag/cohort/leipzig-obesity-bio-bank-lobb), which comprises paired samples of abdominal subcutaneous and omental visceral adipose tissue. The metabolically healthy versus unhealthy obese cohort (MHO/MUO) comprises paired samples of omental visceral and abdominal subcutaneous tissues from 31 insulin-sensitive patients (71% women; age: 38.8 ± 11.1 years old; BMI: 45.9 ± 6.9 kg/m²; fasting plasma glucose: 5.2 ± 0.2 mmol/l; fasting plasma insulin: 27.9 ± 13.5 pmol/l) and 42 insulin-resistant patients (71.43% female; age: 47.2 ± 7.7 years old; BMI: 47.3 ± 8.1 kg/m²; fasting plasma glucose: 5.7 ± 0.3 mmol/l; fasting plasma insulin: 113.7 ± 45.7 pmol/l). The cross-sectional cohort (CSC) comprises 1,479 individuals, categorized as either normal/overweight (N = 31; 52% women; age: 55.8 ± 13.4 years; BMI: 25.7 ± 2.7 kg/m²) or obese (N = 1,448; 71% women; age: 46.9 ± 11.7 years; BMI: 49.2 ± 8.3 kg/m²). Adipose tissue samples were collected during elective laparoscopic abdominal surgeries, following established protocols57,58. Body composition and metabolic parameters were assessed using standardized techniques as described previously59,60. The study was approved by the Ethics Committee of the University of Leipzig (approval numbers: 363-10-13122010 and 017-12-230112) and adhered to the principles outlined in the Declaration of Helsinki. All participants provided written informed consent before being included in the study. Participants received no compensation for their tissue donation to the Leipzig Obesity BioBank. Exclusion criteria included individuals under 18 years of age, those with chronic substance or alcohol abuse, smoking within the 12 months prior to surgery, acute inflammatory conditions, concurrent use of glitazones, end-stage malignancies, weight loss greater than 3% in the three months leading up to surgery, uncontrolled thyroid disorders, and Cushing's disease.<br><br>In analysis of the RNA-seq data, normalization was carried out using a weighted trimmed mean (TMM) of the log expression ratios, with adjustments made for age and sex.
Reporting on race, ethnicity, or other socially relevant groupings	*Please specify the socially constructed or socially relevant categorization variable(s) used in your manuscript and explain why they were used. Please note that such variables should not be used as proxies for other socially constructed/relevant variables (for example, race or ethnicity should not be used as a proxy for socioeconomic status).*<br>*Provide clear definitions of the relevant terms used, how they were provided (by the participants/respondents, the researchers, or third parties), and the method(s) used to classify people into the different categories (e.g. self-report, census or administrative data, social media data, etc.)*<br>*Please provide details about how you controlled for confounding variables in your analyses.*
Population characteristics	Human data were obtained from the Leipzig Obesity Biobank (LOBB), which includes paired abdominal subcutaneous and omental visceral adipose tissue samples. The metabolically healthy vs. unhealthy obese cohort (MHO/MUO) comprised 31 insulin-sensitive patients (71% female; age 38.8 ± 11.1 years; BMI 45.9 ± 6.9 kg/m²) and 42 insulin-resistant patients (71.43% women age 47.2 ± 7.7 years; BMI 47.3 ± 8.1 kg/m²), with corresponding fasting glucose and insulin measures. The cross-sectional cohort (CSC) included 1,479 individuals categorized as normal/overweight (N = 31; 52% women; age 55.8 ± 13.4 years; BMI 25.7 ± 2.7 kg/m²) or obese (N = 1,448; 71% women; age 46.9 ± 11.7 years; BMI 49.2 ± 8.3 kg/m²). Analyses were adjusted for age and sex.
Recruitment	The human data used in this research were sourced from the Leipzig Obesity Biobank (LOBB; https://www.helmholtz-munich.de/en/hi-mag/cohort/leipzig-obesity-bio-bank-lobb), which comprises paired samples of abdominal subcutaneous and omental visceral adipose tissue. The samples are provided by patients of Leipzig University Hospital and cooperating obesity centers in Germany as part of obesity treatment after detailed information and the consent of the participants. Adipose tissue samples were collected during elective laparoscopic abdominal surgeries, following established protocols. The study was approved by the Ethics Committee of the University of Leipzig (approval numbers: 363-10-13122010 and 017-12-230112) and adhered to the principles outlined in the Declaration of Helsinki. All participants provided written informed consent before being included in the study. Exclusion criteria included individuals under 18 years of age, those with chronic substance or alcohol abuse, smoking within the 12 months prior to surgery, acute inflammatory conditions, concurrent use of glitazones, end-stage malignancies, weight loss greater than 3% in the three months leading up to surgery, uncontrolled thyroid disorders, and Cushing's disease.<br>Two cohorts were included in this study. The metabolically healthy versus unhealthy obese cohort (MHO/MUO) comprises paired samples of omental visceral and abdominal subcutaneous tissues from 31 insulin-sensitive patients and 42 insulin-resistant patients. In addition, the cross-sectional cohort (CSC) comprises 1,479 individuals categorized as either normal/overweight (N = 31) or obese (N = 1,448). Body composition and metabolic parameters were assessed using standardized techniques as described previously.<br>Potential sources of bias include self-selection bias and sampling bias, as participants were recruited from individuals undergoing obesity treatment and elective surgical procedures and who consented to participation. This recruitment strategy

	may enrich the cohort for individuals with more severe obesity and/or obesity-related comorbidities and may therefore not fully represent the broader population of individuals with obesity. In addition, the cohort is predominantly female (~71% women in the obese groups), which may limit generalizability to men and may influence observed associations if sex-specific adipose tissue biology contributes to the outcomes measured. Finally, cross-sectional analyses are inherently limited in causal interpretation and may be influenced by residual confounding (e.g., comorbidities, medication use, or lifestyle factors not fully captured). Together, these factors may affect the magnitude and generalizability of observed associations between gene expression and metabolic parameters, although they are less likely to invalidate within-cohort comparisons when analyses are adjusted for key covariates such as age and sex.
Ethics oversight	The study was approved by the Ethics Committee of the University of Leipzig (approval numbers: 363-10-13122010 and 017-12-230112) and adhered to the principles outlined in the Declaration of Helsinki.

Note that full information on the approval of the study protocol must also be provided in the manuscript.

Field-specific reporting

Please select the one below that is the best fit for your research. If you are not sure, read the appropriate sections before making your selection.

☒ Life sciences ☐ Behavioural & social sciences ☐ Ecological, evolutionary & environmental sciences

For a reference copy of the document with all sections, see nature.com/documents/nr-reporting-summary-flat.pdf

Life sciences study design

All studies must disclose on these points even when the disclosure is negative.

Sample size	No statistical method was used to predetermine sample size. Sample sizes were selected based on prior literature and our prior experience with similar experimental systems. These sample sizes are consistent with those commonly used in comparable studies and were sufficient to detect robust and reproducible effects in our assays.
Data exclusions	No data was excluded.
Replication	Each experiment was replicated in at least 2-3 independent studies to ensure the reliability and reproducibility of the findings.
Randomization	The experiments were not randomized.<br>Animals were randomly assigned to different groups, to have the same average body weight in each group.
Blinding	All data analyses and quantifications were conducted blindly.

Reporting for specific materials, systems and methods

We require information from authors about some types of materials, experimental systems and methods used in many studies. Here, indicate whether each material, system or method listed is relevant to your study. If you are not sure if a list item applies to your research, read the appropriate section before selecting a response.

Materials & experimental systems

n/a	Involved in the study	
☐	☒	Antibodies
☐	☒	Eukaryotic cell lines
☒	☐	Palaeontology and archaeology
☐	☒	Animals and other organisms
☒	☐	Clinical data
☒	☐	Dual use research of concern
☒	☐	Plants

Methods

n/a	Involved in the study	
☒	☐	ChIP-seq
☐	☒	Flow cytometry
☒	☐	MRI-based neuroimaging

Antibodies

Antibodies used	The antibodies are listed in Supplementary Table 2.
Validation	Anti-Slit3 antibody R&D Systems (AF3629): Slit3 was detected in immersion fixed paraffin-embedded sections of 13 d.p.c. mouse embryo using Goat Anti-Human/Mouse/Rat Slit3 Antigen Affinity-purified Polyclonal Antibody (Catalog # AF3629) at 5 µg/mL for 1 hour at room temperature followed by incubation with the Anti-Goat IgG VisUCyte™ HRP Polymer Antibody (VC004). Before incubation with the primary antibody, tissue was subjected to heat-induced epitope retrieval using Antigen Retrieval Reagent-Basic (CTS013). Tissue was stained using DAB (brown) and counterstained with hematoxylin (blue). Specific staining was localized to developing brain and cartillage.<br><br>Anti-Tyrosine Hydroxylase antibody Millipore Sigma (AB1542): Routinely evaluated by Western Blot on mouse brain lysates.

Anti-UCP1 antibody Abcam (Ab23841): In Western blot, ab23841 was shown to bind specifically to UCP1 from Adult Mouse and Rat Brown Adipose Tissue.

Anti-Total OXPHOS antibody Abcam (Ab110413): used to detect OXPHOS proteins from Rat liver mitochondria, Isolated mitochondria from mice brain, Isolated mitochondria from heart of human, Human skeletal muscle tissue lysate, and other sources.

Anti- β Actin antibody Cell Signaling (3700S): β-Actin (8H10D10) Mouse mAb detects endogenous levels of total β-actin protein in various cell types and tissues.

Anti-Perilipin 1 antibody Cell Signaling (9349S): Perilipin-1 (D1D8) XP® Rabbit mAb detects endogenous levels of total perilipin-1 protein in adipocytes and adipose tissue.

Anti-Robo1 antibody Abcam (ab7279): Rabbit Polyclonal ROBO1 antibody. Suitable for ELISA, WB, IHC-P, IHC-Fr and reacts with Synthetic peptide, Mouse, Human samples. Cited in 54 publications. Immunogen corresponding to Synthetic Peptide within Human ROBO1 aa 1600-1650 conjugated to Keyhole Limpet Haemocyanin.

Anti-Robo4 antibody Santa Cruz Biotech (sc-166872): robo4 Antibody (D-3) is a mouse monoclonal IgM κ robo4 antibody, cited in 5 publications, provided at 200 µg/ml. Western blot analysis of robo4 expression in K-562 (A) and HUV-EC-C (B) whole cell lysates.

Anti-Plexin A1 antibody Abcam (ab23391): Rabbit Polyclonal Plexin A1 antibody. Suitable for IP, ELISA, WB, ICC/IF and reacts with Mouse, Rat, Human, Transfected cell lysate - Mouse samples. Cited in 13 publications. Immunogen corresponding to Synthetic Peptide within Human PLXNA1 aa 150-200. Western blots showing Cos-7 cells transfected with mouse myc-tagged Plexin-A1, neonatal rat brain, or Plexin-A1 immunoprecipitated from myc-tagged Plexin-A1 transfected cells using anti-Myc or anti-Plexin-A1 (ab23391).

Anti-SNAP-tag antibody New England Biolabs (P9310S): The Anti-SNAP-tag® Antibody (Polyclonal) can be used for the detection of SNAP-tagged fusion proteins in Western blots (249 Citations).

Anti-HaloTag antibody Promega (G9211): Anti-HaloTag® Monoclonal Antibody is a mouse monoclonal antibody raised against the HaloTag® protein, which can be used to detect HaloTag® fusion proteins by Western blotting. Little to no cross-reactivity with other non-HaloTag® proteins.

PE anti-mouse F4/80 Antibody Biolegend (123110): Staining of macrophages inThioglycolate-elicited BALB/c mouse peritoneal macrophages stained with BM8 PE.

PE/Cyanine7 anti-mouse CD117 (c-Kit) Antibody Biolegend (105814): C57BL/6 mouse splenocytes were stained with anti-mouse CD117 (c-Kit) (clone 2B8) PE/Cyanine7 or , κ PE/Cyanine7 isotype control (right).

PE/Cyanine7 anti-mouse/human CD11b Antibody Biolegend (101216): C57BL/6 mouse bone marrow cells were stained with CD11b (clone M1/70) PE/Cyanine7 or rat IgG2b, κ PE/Cyanine7 isotype control.

PE/Cyanine7 anti-mouse CD45 Antibody Biolegend (103114): C57BL/6 mouse splenocytes stained with 30-F11 PE/Cyanine7.

Alexa Fluor® 488 anti-mouse CD31 Antibody Biolegend (102514): C57BL/6 mouse splenocytes stained with MEC13.3 Alexa Fluor® 488

Ly-6A/E (Sca-1) Monoclonal Antibody (D7), PerCP-Cyanine5.5 eBioscience (5015865): Staining of C57Bl/6 bone marrow cells with Mouse Hematopoietic Lineage and 98 published figures are shown.

Brilliant Violet 421™ anti-mouse CD140a Antibody Biolegend (135923): Mouse fibroblast NIH/3T3 cells were stained with CD140a (clone APA5) Brilliant Violet 421™ (or Rat IgG2a, κ Brilliant Violet 421™ isotype control.

Eukaryotic cell lines

Policy information about cell lines and Sex and Gender in Research

Cell line source(s)	Immortalized mouse brown fat SVF cell line (Derived from the stromal vascular fraction of mouse brown adipose tissue
Authentication	The cell line is not authenticated.
Mycoplasma contamination	The cell line was tested negative for Mycoplasma contamination.
Commonly misidentified lines (See ICLAC register)	No commonly misidentified cell line was used.

Animals and other research organisms

Policy information about studies involving animals; ARRIVE guidelines recommended for reporting animal research, and Sex and Gender in Research

Laboratory animals	Male Mus musculus (C57BL6J, stock no. 000664), aged 8–16 weeks, were used for studies involving AAV administration. Pdgfra-creER;Slit3^flox/flox or Slit3^iΔAPC mice were generated by crossing Slit3 floxed mice with the Pdgfra-creER strain (JAX strain 018280). Both male and female mice, aged 10–16 weeks, were included.
Wild animals	Study did not include wild animals.
Reporting on sex	Information regarding the sex of the animals is included in the manuscript.
Field-collected samples	Study did not involve samples collected from the field.
Ethics oversight	All experimental procedures involving animals were performed in compliance with all relevant ethical regulations applied to the use of small rodents and with approval by the Institutional Animal Care and Use Committees at New York University.

Note that full information on the approval of the study protocol must also be provided in the manuscript.

Plants

Seed stocks	*Report on the source of all seed stocks or other plant material used. If applicable, state the seed stock centre and catalogue number. If plant specimens were collected from the field, describe the collection location, date and sampling procedures.*
Novel plant genotypes	*Describe the methods by which all novel plant genotypes were produced. This includes those generated by transgenic approaches, gene editing, chemical/radiation-based mutagenesis and hybridization. For transgenic lines, describe the transformation method, the number of independent lines analyzed and the generation upon which experiments were performed. For gene-edited lines, describe the editor used, the endogenous sequence targeted for editing, the targeting guide RNA sequence (if applicable) and how the editor was applied.*
Authentication	*Describe any authentication procedures for each seed stock used or novel genotype generated. Describe any experiments used to assess the effect of a mutation and, where applicable, how potential secondary effects (e.g. second site T-DNA insertions, mosiacism, off-target gene editing) were examined.*

Flow Cytometry

Plots

Confirm that:

- [x] The axis labels state the marker and fluorochrome used (e.g. CD4-FITC).
- [x] The axis scales are clearly visible. Include numbers along axes only for bottom left plot of group (a 'group' is an analysis of identical markers).
- [x] All plots are contour plots with outliers or pseudocolor plots.
- [x] A numerical value for number of cells or percentage (with statistics) is provided.

Methodology

Sample preparation	Adipocytes and the Stromal Vascular Fraction (SVF) were isolated from mouse BAT following the procedure described previously5. The interscapular BAT was dissected, finely minced, and digested for 45 minutes using a cocktail containing type 1 collagenase (1.5 mg ml−1; Worthington Biochemical), dispase II (2.5 U ml−1; Stemcell Technologies), and fatty acid-free BSA (2%; Gemini Bio-Products) in Hanks' balanced salt solution (Corning Hanks' Balanced Salt Solution, with calcium and magnesium). The resulting dissociated tissue was subsequently centrifuged at 500g and 4°C for 10 minutes. Adipocytes, located in the uppermost layer, were gently collected using a wide-mouthed transfer pipette and filtered through a 100 µm cell strainer. Brown adipocytes were allowed to float for 5 min at room temperature before they were centrifuged at 30g for 5 minutes at room temperature. This cycle was repeated three times, after which the adipocytes were immediately lysed in Trizol. For the SVF isolation, the pellet was resuspended in 10 ml of 10% FBS in DMEM, filtered through a 100 µm cell strainer into a fresh 50-ml tube, and subsequently centrifuged at 500g for 7 minutes. Red blood cells were lysed in 2 ml of sterile ammonium-chloride-potassium lysis buffer (ACK Lysis Buffer, Lonza) for 5 minutes on ice. The cells were then filtered once more through a 40-µm cell strainer, washed with 20 ml of a solution containing 10% FBS in DMEM, and centrifuged at 500g for 7 minutes. The cells were resuspended in 1 ml of Cell Staining Buffer (BioLegend) before proceeding with staining.<br>Cells were stained using the fluorescently conjugated antibodies as outlined in Supplementary Table 2. The cells were then incubated with the antibodies at the specified dilutions from Supplementary Table 2 for a duration of 30 minutes, followed by two rounds of washing in Cell Staining Buffer (BioLegend). Cells were sorted using an SH800 sorter using a 100 µm sorting chip (Sony Biotechnology). Debris and doublets were excluded based on forward and side scatter gating, and 7-AAD was used to exclude dead cells. Following sorting, cells were centrifuged at 300 g for 5 minutes and lysed in Trizol for subsequent RNA isolation and gene expression analysis.
Instrument	SONY SH800 Cell Sorter

Software	Sony Cell Sorter Software
Cell population abundance	The cell population abundance is reported in Supplementary Figure 1f.
Gating strategy	The gating is reported in Supplementary Figure 1f. Cells were gated on FSC-A vs SSC-A to exclude debris, singlets were selected by FSC-H vs FSC-A, and viable cells were defined as 7-AAD negative. Macrophages were identified as Lin+ F4/80+. PDGFRα+ APCs were defined as CD140a+ Ly-6A+ within live singlets. The lineage markers include CD45, CD11b, and CD117.

☒ Tick this box to confirm that a figure exemplifying the gating strategy is provided in the Supplementary Information.
