# Supplementary material for: SLIT3 fragments orchestrate neurovascular expansion and thermogenesis in brown adipose tissue
Source: Nat Commun. 2026 Mar 25;17:2445. doi: 10.1038/s41467-026-70310-9 (PMC13018599; doi:10.1038/s41467-026-70310-9)

Figure 1c

Anti- Slit3 - FL

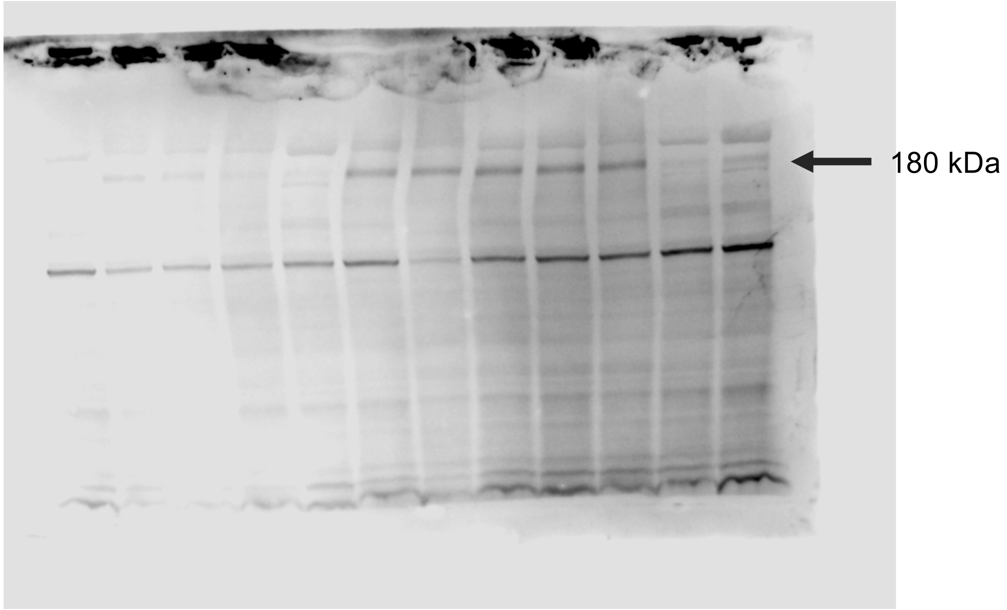

Anti- β-actin

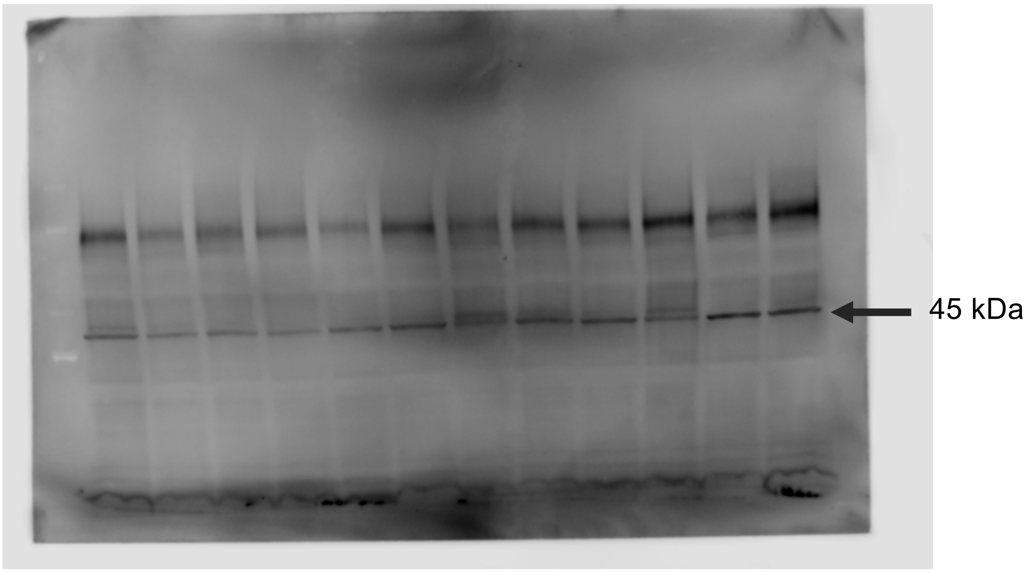

Figure 1g

Anti-β-actin and anti-UCP1

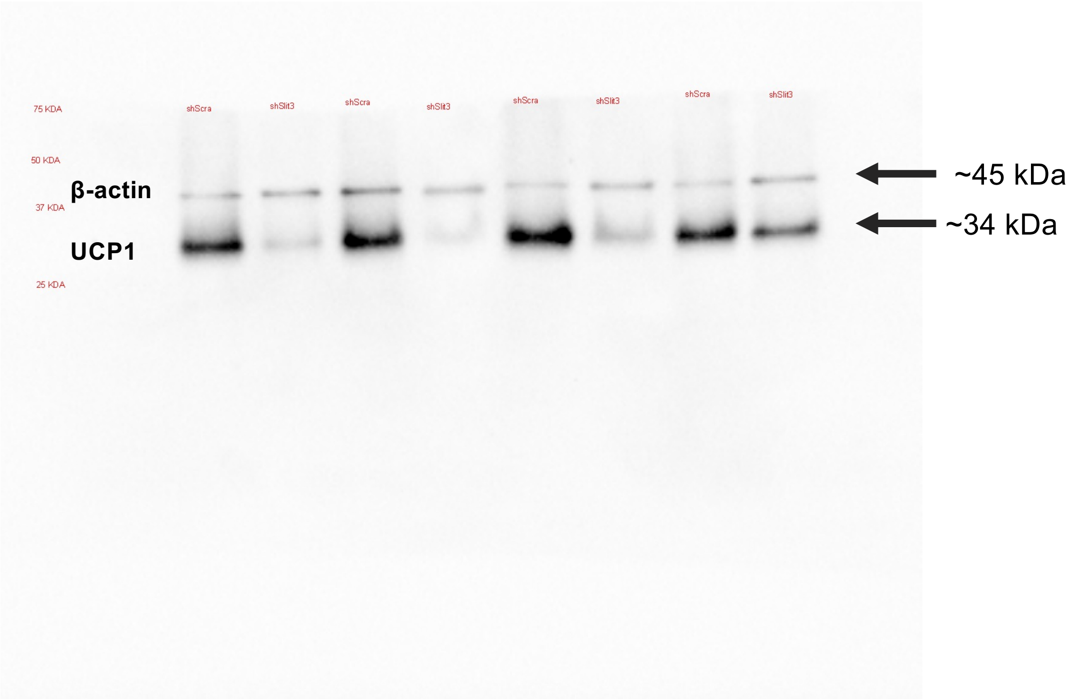

Total Protein

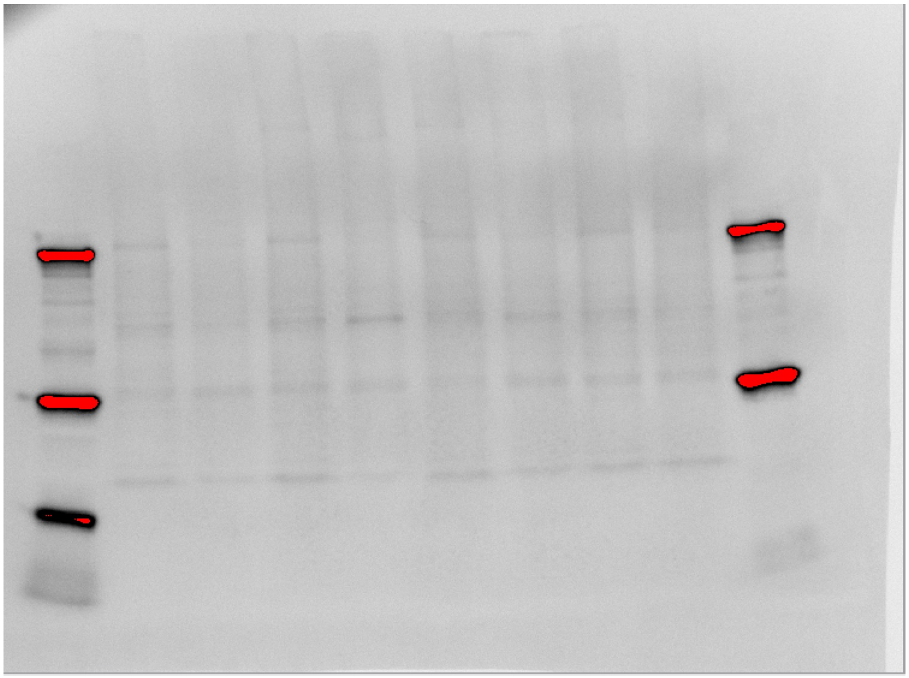

Figure 1j

Anti- OXPHOS

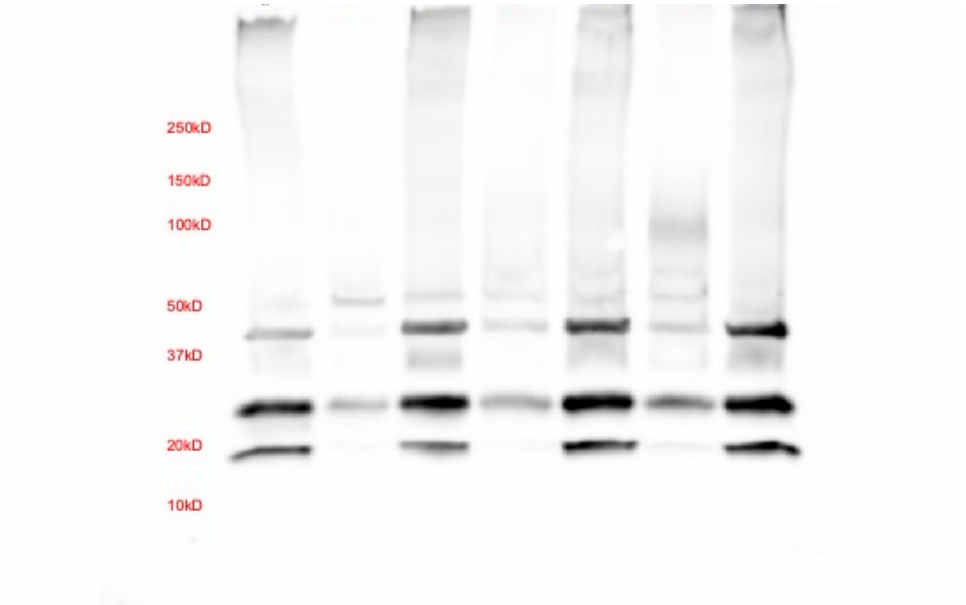

Total Protein

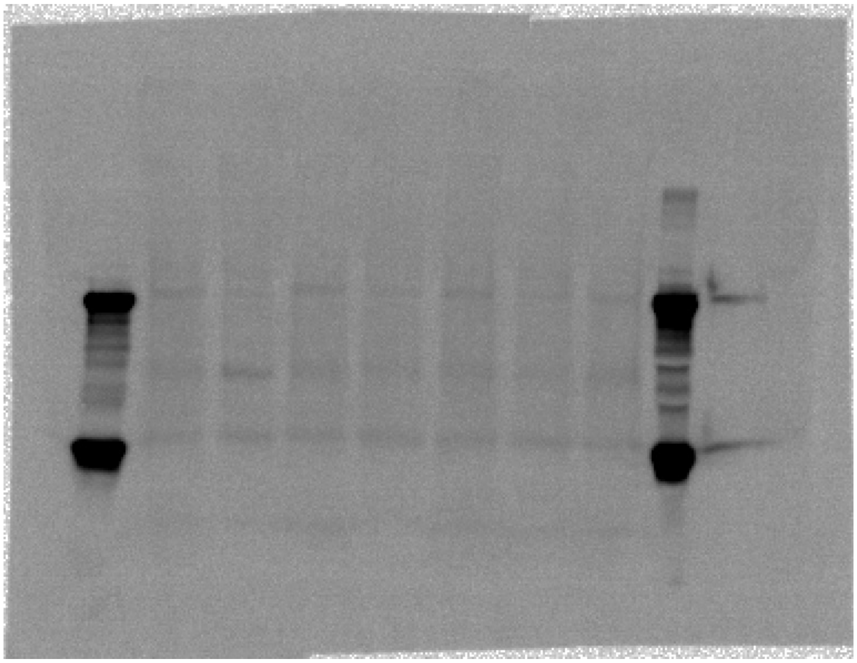

Figure 2i

Anti- Vinculin

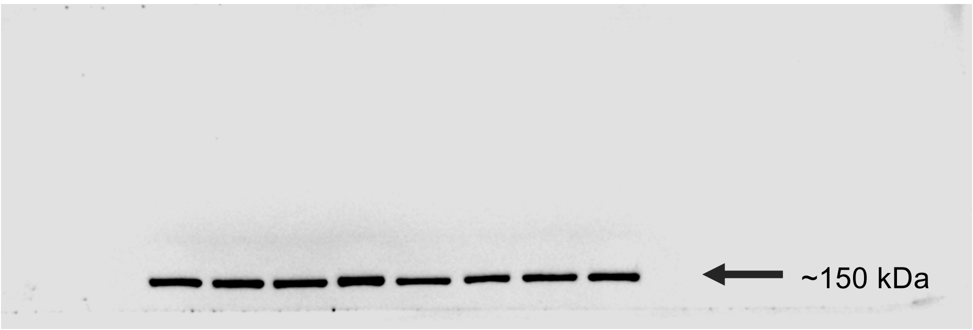

Anti- TH

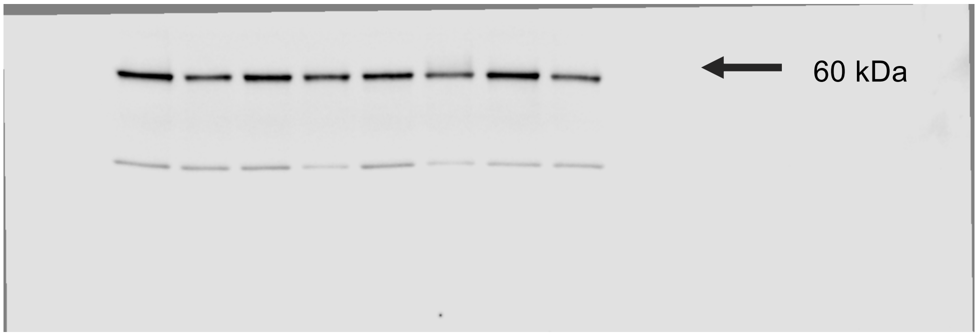

Total Protein

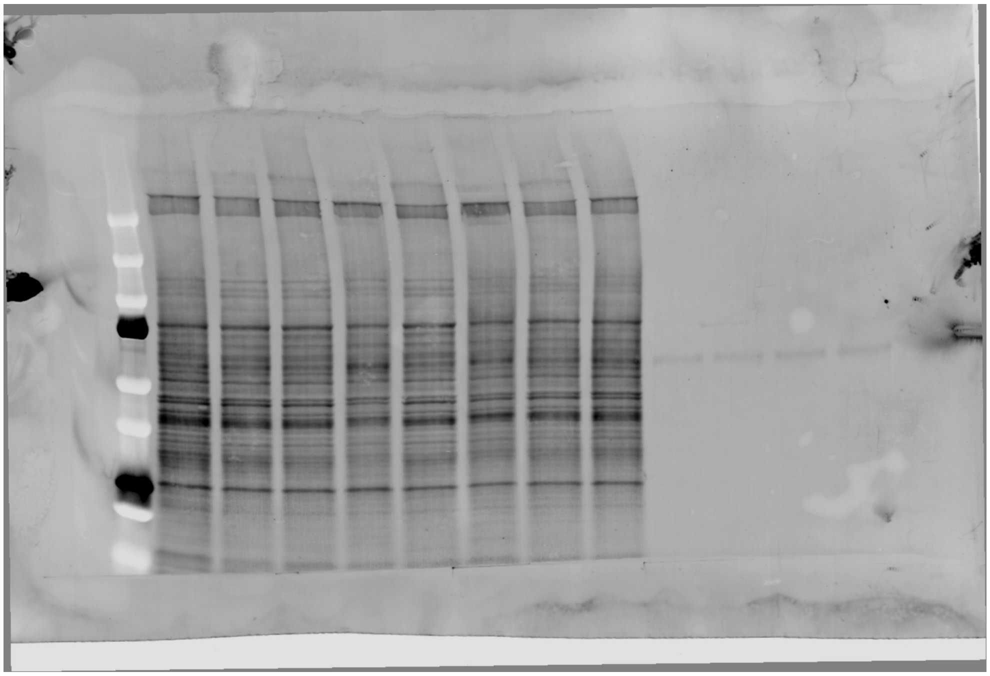

Experimental Analysis

520 Channel

Image Name: TP_shSlit3cold7days_ 18ug_memb1

Lane Detection: Manual

Protein: TP

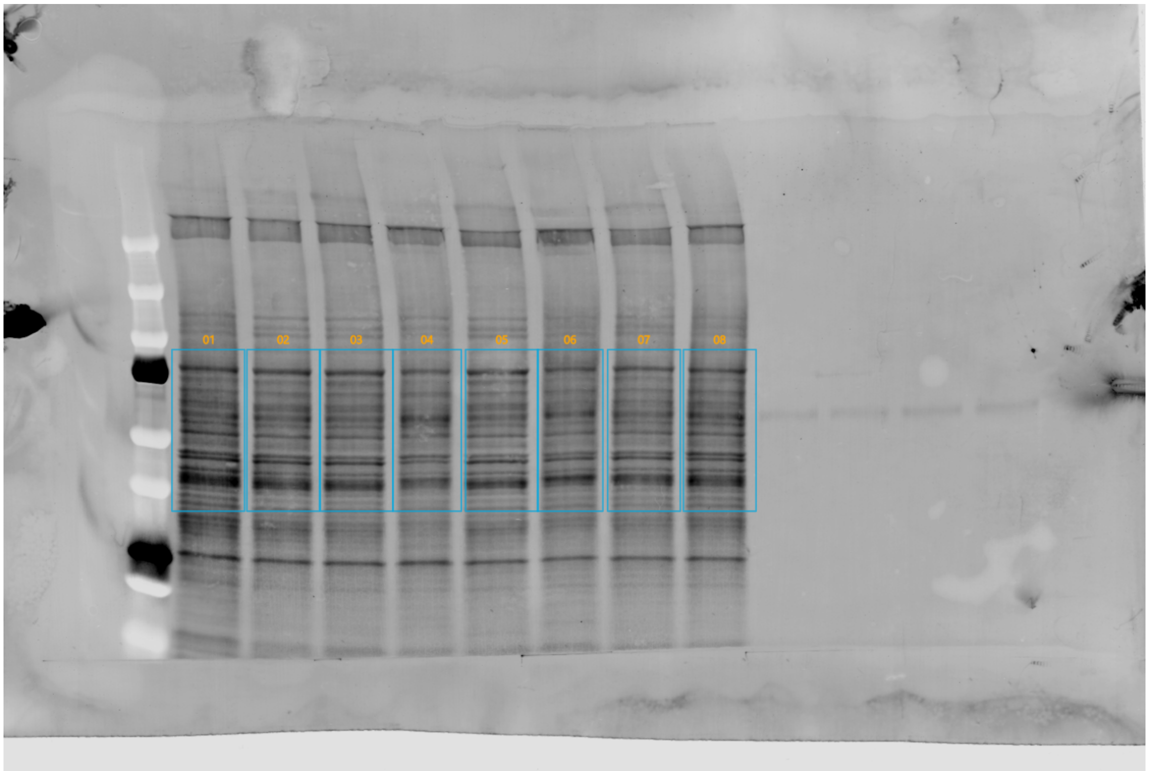

Total Protein Stain Analysis Table

Lane	Name	Signal	SNR	Replicate	Avg. Signal	Avg. SNR	Std. Dev.	% CV	Treatment (%)	Type
01	Lane01	15,400,000	27.5	WT	11,800,000	21.1	2,470,000	20.9	0	Sample
02	Lane02	11,700,000	20.9	KO	10,800,000	20.2	1,320,000	12.1	0	Sample
03	Lane03	11,300,000	20.1	WT	11,800,000	21.1	2,470,000	20.9	0	Sample
04	Lane04	9,160,000	17.5	KO	10,800,000	20.2	1,320,000	12.1	0	Sample
05	Lane05	9,940,000	17.8	WT	11,800,000	21.1	2,470,000	20.9	0	Sample
06	Lane06	10,500,000	20.9	KO	10,800,000	20.2	1,320,000	12.1	0	Sample
07	Lane07	10,600,000	18.9	WT	11,800,000	21.1	2,470,000	20.9	0	Sample
08	Lane08	12,100,000	21.6	KO	10,800,000	20.2	1,320,000	12.1	0	Sample

Chemi Channel

Image Name: TH_memb1

Lane Detection: Manual

Protein: TH

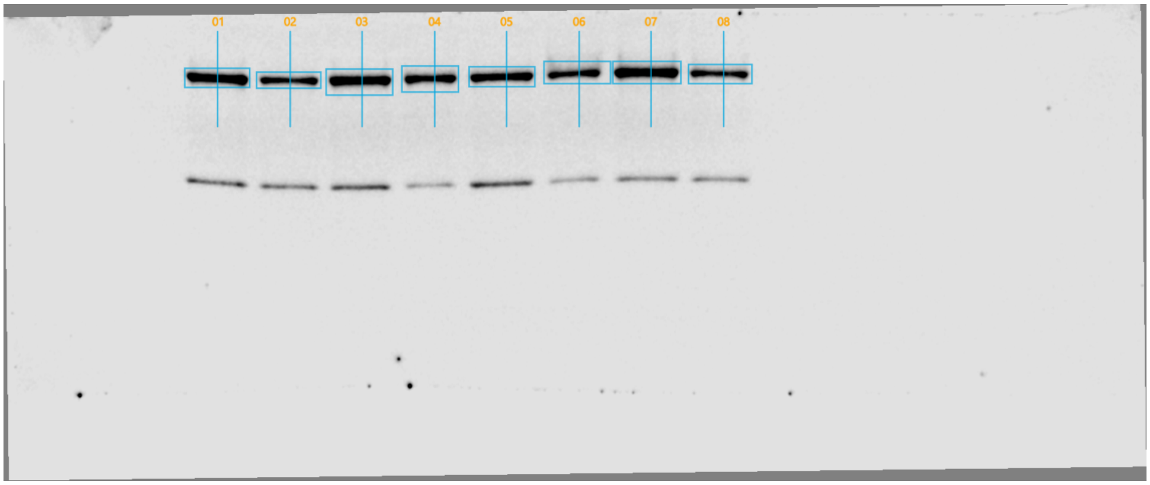

Protein Analysis Table

Lane	Name	MW	Normalized Signal	SNR	Replicate	Avg. Norm. Signal	Avg. SNR	Std. Dev.	% CV	Treatment (%)	Type
01	Lane01		4.07	57.1	WT	5.33	46.5	1.15	21.6	0	Sample
02	Lane02		2.47	35.9	KO	2.92	28.1	0.711	24.3	0	Sample
03	Lane03		5.66	38.5	WT	5.33	46.5	1.15	21.6	0	Sample
04	Lane04		3.89	25.2	KO	2.92	28.1	0.711	24.3	0	Sample
05	Lane05		4.85	42.1	WT	5.33	46.5	1.15	21.6	0	Sample
06	Lane06		3.00	25.2	KO	2.92	28.1	0.711	24.3	0	Sample
07	Lane07		6.76	48.5	WT	5.33	46.5	1.15	21.6	0	Sample
08	Lane08		2.32	26.1	KO	2.92	28.1	0.711	24.3	0	Sample

RGB Epi Channel

Image Name: TH_memb1

Lane Detection: Manual

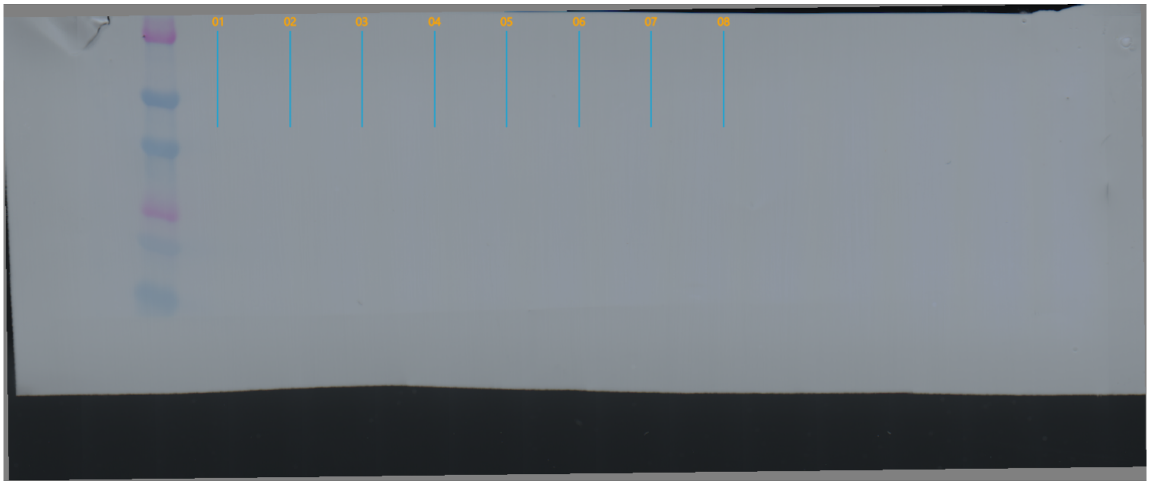

Summary

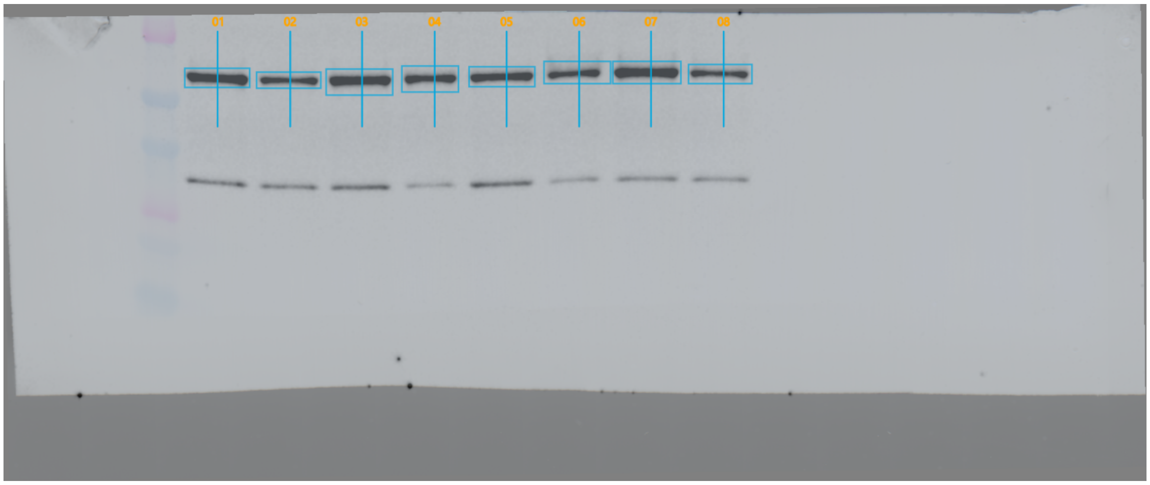

Experimental Observations
Not specified

Next Steps
Not specified

Summary
Not specified

Figure 3a

Anti- Slit3-FL

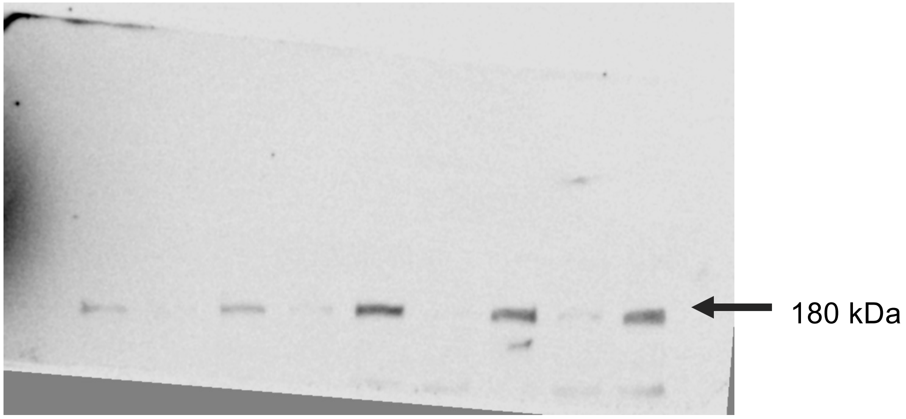

Anti- Vinculin

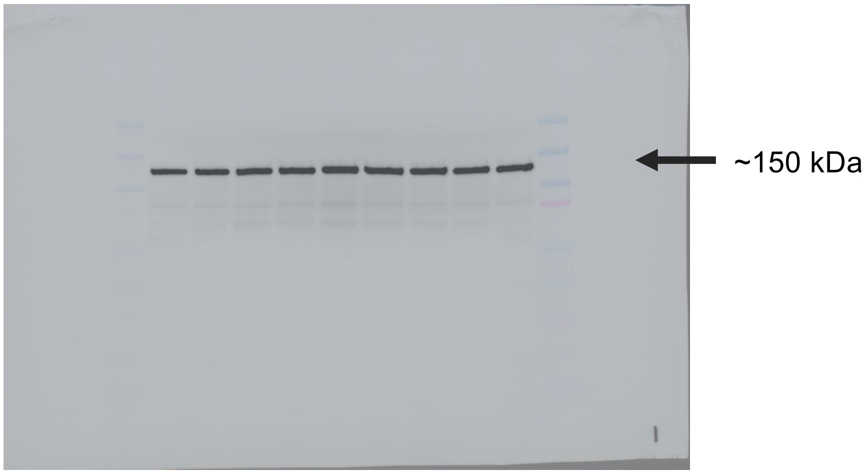

Total Protein

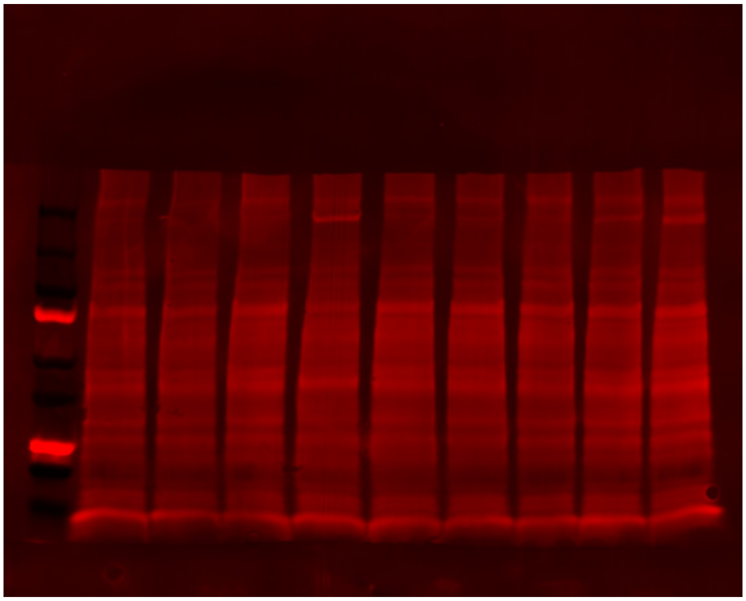

Experimental Analysis

520 Channel

Image Name: ERT2 Cohort 4 - BAT TP 20 ug

Lane Detection: Automatic Lane Finding

Protein: Total Protein

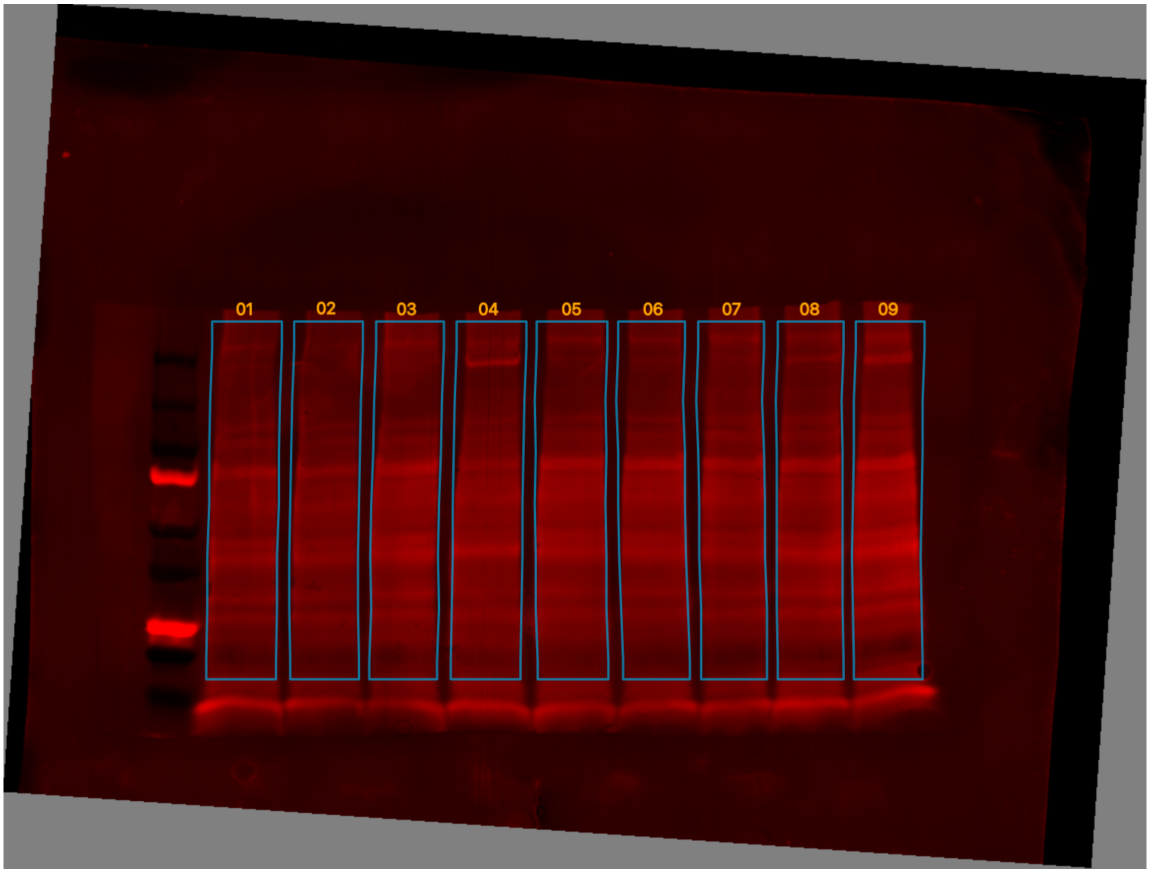

Total Protein Stain Analysis Table

Lane	Name	Signal	SNR	Replicate	Avg. Signal	Avg. SNR	Std. Dev.	% CV	Treatment (%)	Type
01	Lane01	20,300,000	7.47	WT	24,800,000	9.31	3,870,000	15.6	0	Sample
02	Lane02	18,700,000	6.98	KO	22,900,000	8.69	3,110,000	13.6	0	Sample
03	Lane03	24,900,000	9.44	WT	24,800,000	9.31	3,870,000	15.6	0	Sample
04	Lane04	24,700,000	9.09	KO	22,900,000	8.69	3,110,000	13.6	0	Sample
05	Lane05	25,500,000	9.28	WT	24,800,000	9.31	3,870,000	15.6	0	Sample
06	Lane06	22,500,000	8.66	KO	22,900,000	8.69	3,110,000	13.6	0	Sample
07	Lane07	22,800,000	8.90	WT	24,800,000	9.31	3,870,000	15.6	0	Sample
08	Lane08	25,700,000	10.0	KO	22,900,000	8.69	3,110,000	13.6	0	Sample
09	Lane09	30,700,000	11.5	WT	24,800,000	9.31	3,870,000	15.6	0	Sample

Chemi Channel

Image Name: ERT2 Cohort 4 - BAT (Slit3)

Lane Detection: Manual

Protein: Slit3

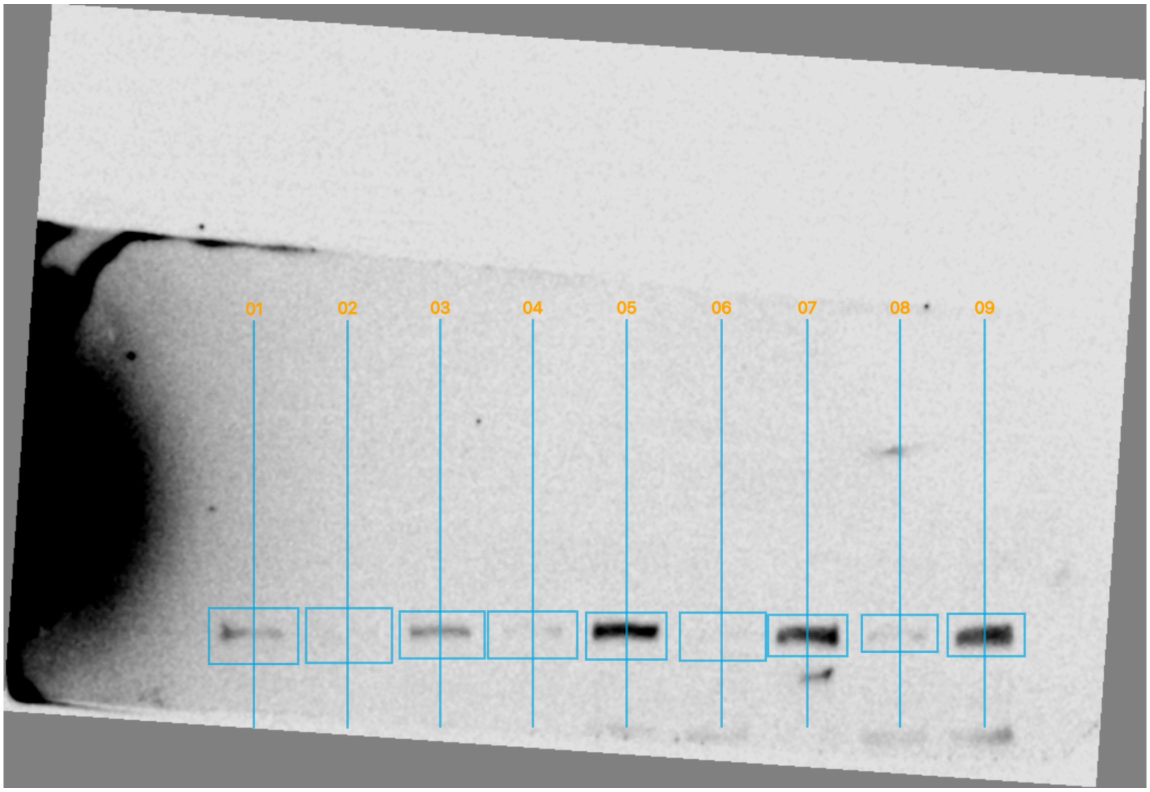

Protein Analysis Table

Lane	Name	MW	Normalized Signal	SNR	Replicate	Avg. Norm. Signal	Avg. SNR	Std. Dev.	% CV	Treatment (%)	Type
01	Lane01		0.084	0.445	WT	0.277	7.24	0.168	60.8	0	Sample
02	Lane02		0.022	0.256	KO	0.019	0.417	0.01	52.0	0	Sample
03	Lane03		0.122	3.14	WT	0.277	7.24	0.168	60.8	0	Sample
04	Lane04		0.027	0.639	KO	0.019	0.417	0.01	52.0	0	Sample
05	Lane05		0.449	11.3	WT	0.277	7.24	0.168	60.8	0	Sample
06	Lane06		0.005	0.107	KO	0.019	0.417	0.01	52.0	0	Sample
07	Lane07		0.424	10.3	WT	0.277	7.24	0.168	60.8	0	Sample
08	Lane08		0.022	0.664	KO	0.019	0.417	0.01	52.0	0	Sample
09	Lane09		0.305	10.9	WT	0.277	7.24	0.168	60.8	0	Sample

Figure 3c

Anti- Slit3

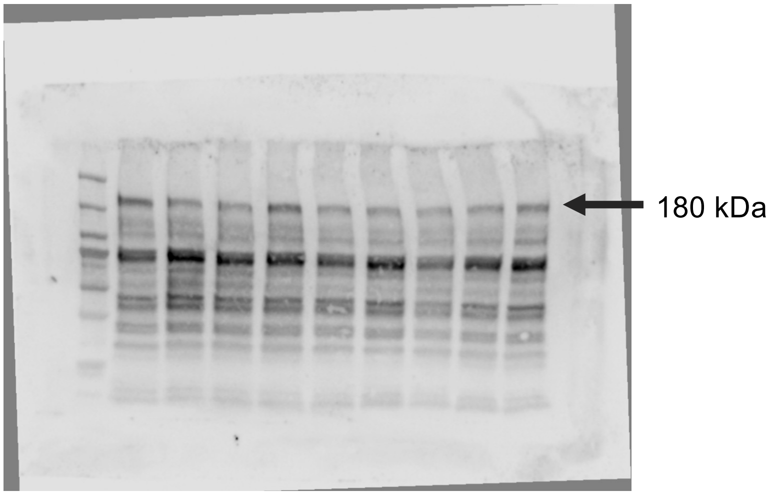

Anti- Vinculin

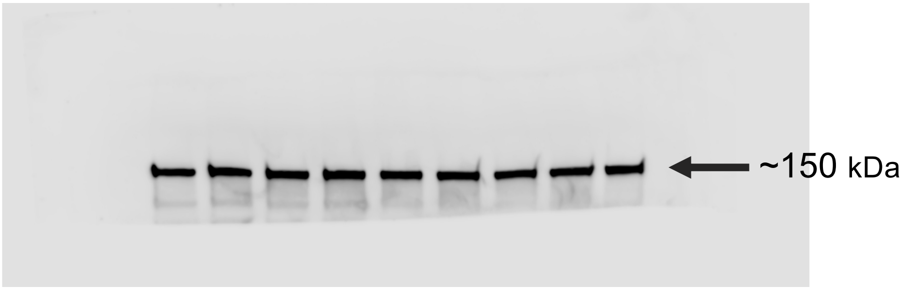

Total Protein

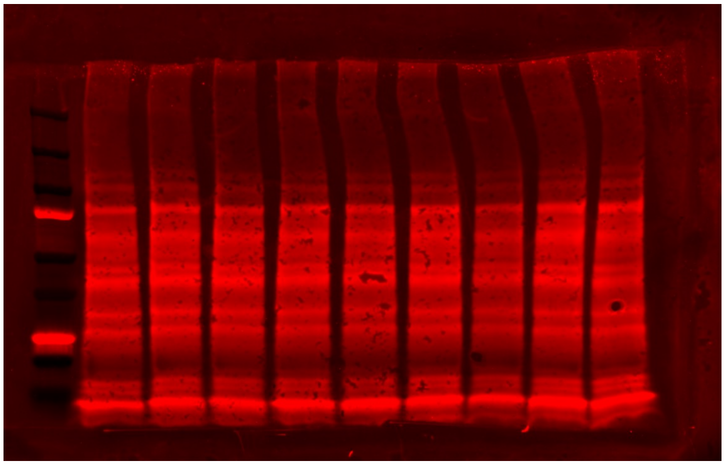

Experimental Analysis

520 Channel

Image Name: ERT2 Cohort 1 RT - Total Protein BAT Females (20ug) separated

Lane Detection: Automatic Lane Finding

Protein: total protein

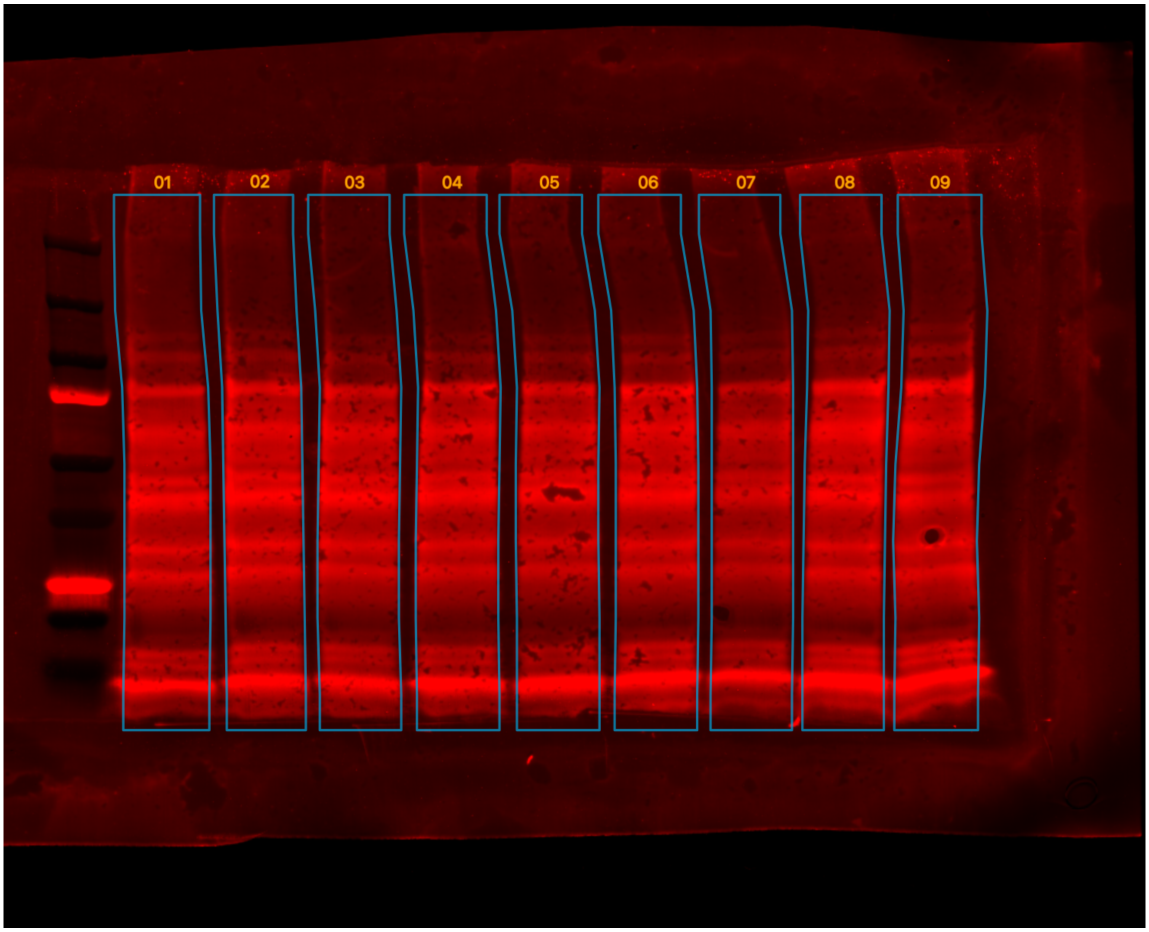

Total Protein Stain Analysis Table

Lane	Name	Signal	SNR	Replicate	Avg. Signal	Avg. SNR	Std. Dev.	% CV	Treatment (%)	Type
01	BAT 10	35,100,000	20.2	WT	33,200,000	20.0	1,950,000	5.89	0	Sample
02	BAT 11	35,000,000	22.0	WT	33,200,000	20.0	1,950,000	5.89	0	Sample
03	BAT 12	32,200,000	19.6	WT	33,200,000	20.0	1,950,000	5.89	0	Sample
04	BAT 13	33,100,000	19.9	WT	33,200,000	20.0	1,950,000	5.89	0	Sample
05	BAT 14	30,400,000	18.3	WT	33,200,000	20.0	1,950,000	5.89	0	Sample
06	BAT 6	34,100,000	20.5	KO	33,700,000	20.3	4,460,000	13.2	0	Sample
07	BAT 7	27,300,000	16.6	KO	33,700,000	20.3	4,460,000	13.2	0	Sample
08	BAT 8	36,800,000	22.4	KO	33,700,000	20.3	4,460,000	13.2	0	Sample
09	BAT 9	36,600,000	21.7	KO	33,700,000	20.3	4,460,000	13.2	0	Sample

Chemi Channel

Image Name: ERT2 Cohort 1 - RT Female BAT Slit3 (20ug)

Lane Detection: Automatic Lane Finding

Protein: Slit3

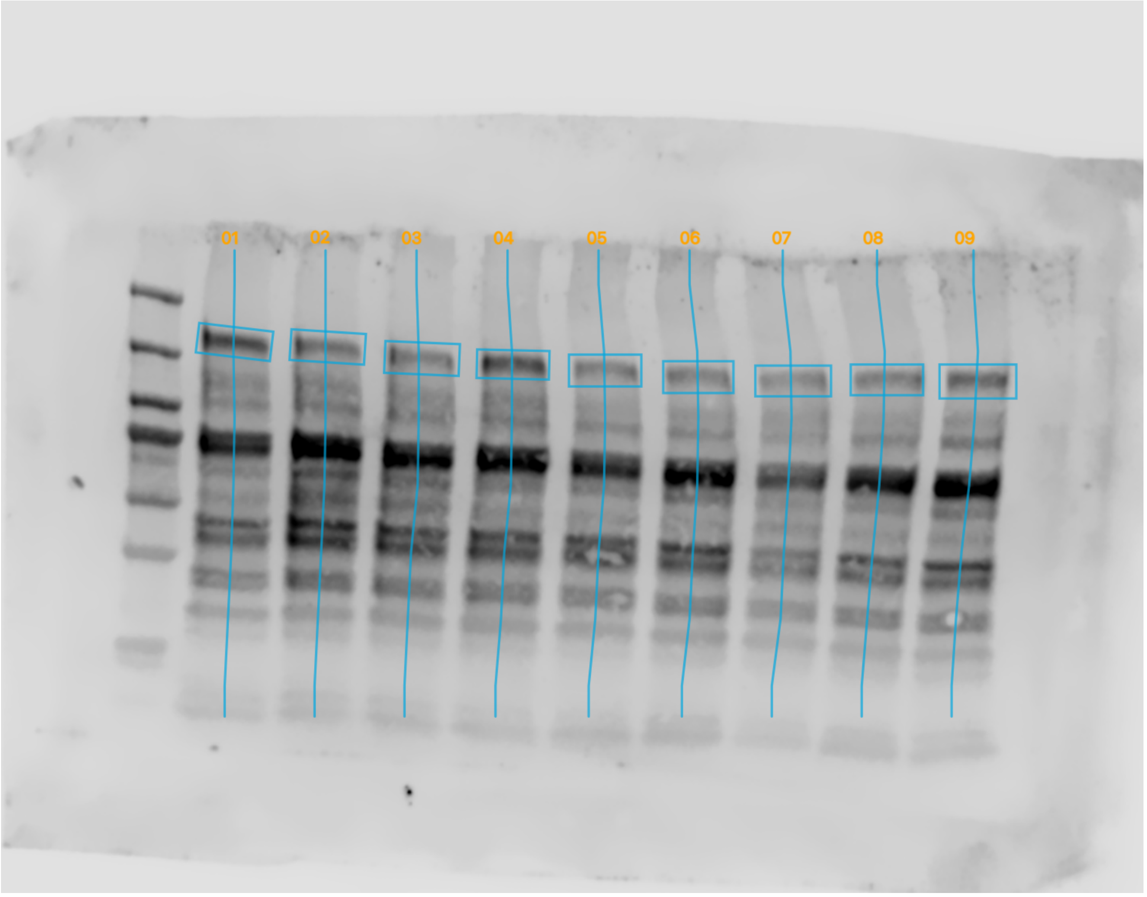

Protein Analysis Table

Lane	Name	MW	Normalized Signal	SNR	Replicate	Avg. Norm. Signal	Avg. SNR	Std. Dev.	% CV	Treatment (%)	Type
01	BAT 10		1.86	12.1	WT	1.51	10.1	0.324	21.4	0	Sample
02	BAT 11		1.34	8.20	WT	1.51	10.1	0.324	21.4	0	Sample
03	BAT 12		1.41	11.7	WT	1.51	10.1	0.324	21.4	0	Sample
04	BAT 13		1.83	12.4	WT	1.51	10.1	0.324	21.4	0	Sample
05	BAT 14		1.11	6.10	WT	1.51	10.1	0.324	21.4	0	Sample
06	BAT 6		0.504	0.827	KO	0.871	4.21	0.286	32.8	0	Sample
07	BAT 7		1.18	6.69	KO	0.871	4.21	0.286	32.8	0	Sample
08	BAT 8		0.97	7.88	KO	0.871	4.21	0.286	32.8	0	Sample
09	BAT 9		0.824	1.45	KO	0.871	4.21	0.286	32.8	0	Sample

Figure 3e

Anti- Slit3

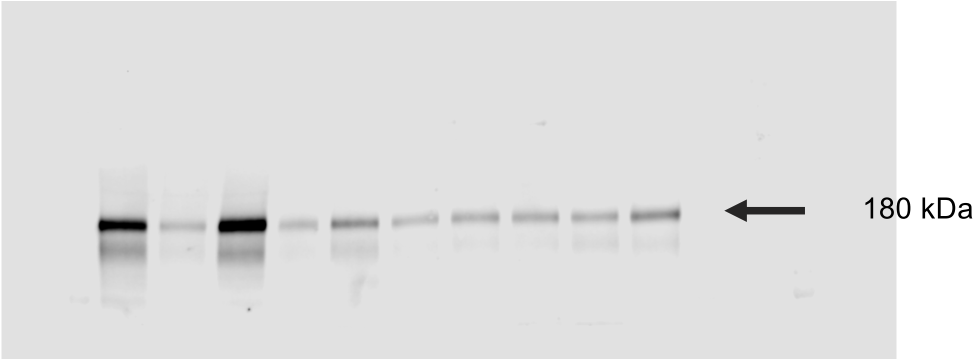

Anti- Vinculin

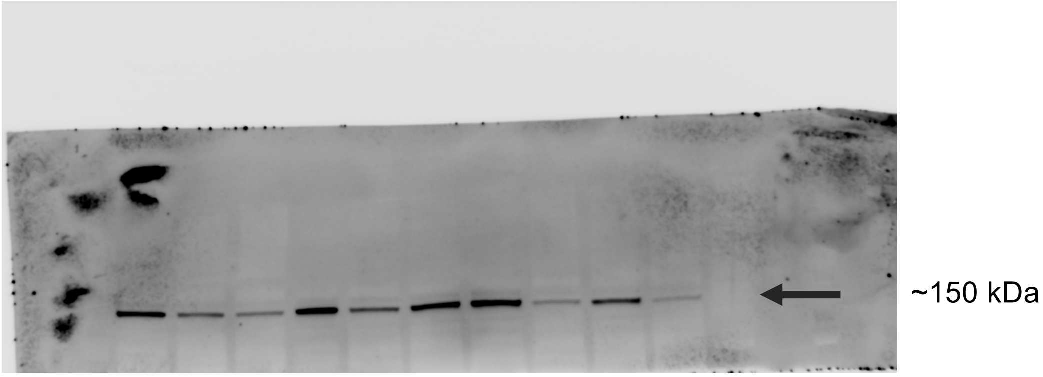

Total Protein

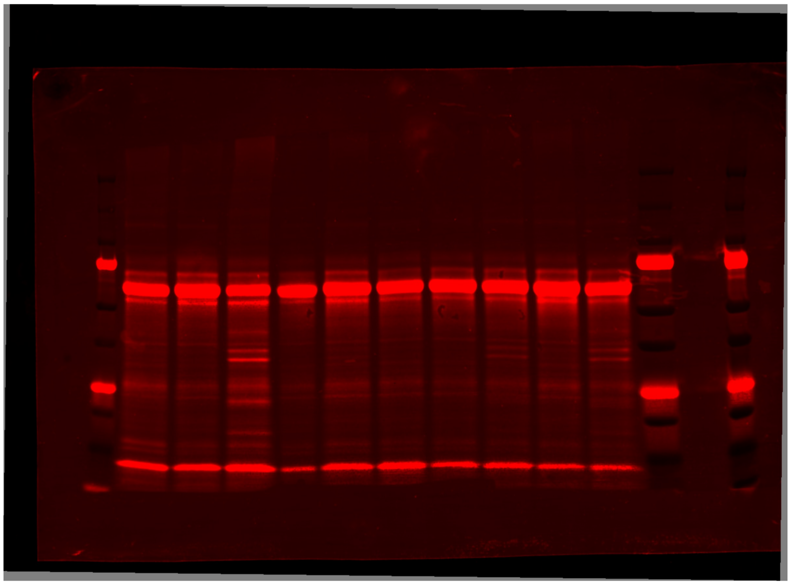

Experimental Analysis

520 Channel

Image Name: ERT2 Cohort 4 - ingWAT total protein redo

Lane Detection: Manual

Protein: total protein

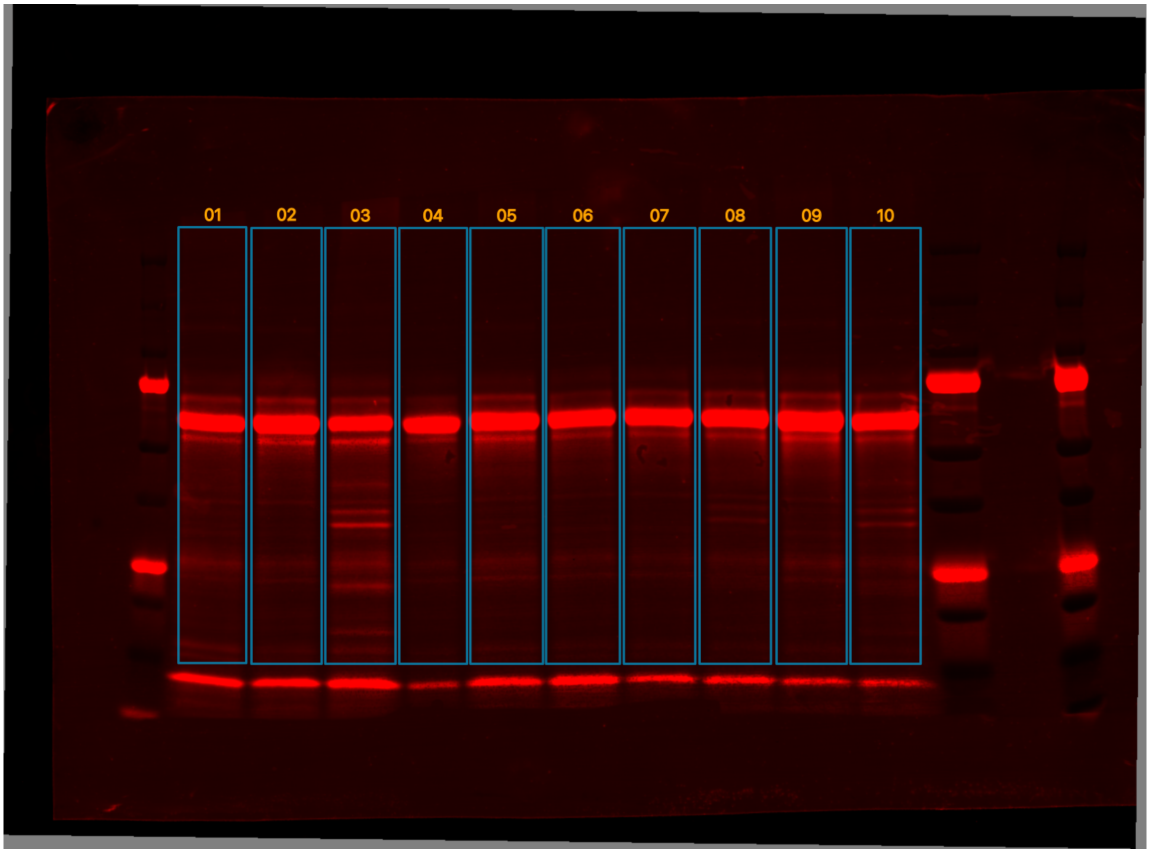

Total Protein Stain Analysis Table

Lane	Name	Signal	SNR	Replicate	Avg. Signal	Avg. SNR	Std. Dev.	% CV	Treatment (%)	Type
01	Lane01	17,100,000	14.8	WT	16,400,000	13.7	2,080,000	12.7	0	Sample
02	Lane02	16,800,000	14.1	KO	13,500,000	11.3	3,030,000	22.4	0	Sample
03	Lane03	18,200,000	15.2	WT	16,400,000	13.7	2,080,000	12.7	0	Sample
04	Lane04	8,990,000	7.77	KO	13,500,000	11.3	3,030,000	22.4	0	Sample
05	Lane05	16,300,000	13.2	WT	16,400,000	13.7	2,080,000	12.7	0	Sample
06	Lane06	12,300,000	9.74	KO	13,500,000	11.3	3,030,000	22.4	0	Sample
07	Lane07	12,900,000	10.5	WT	16,400,000	13.7	2,080,000	12.7	0	Sample
08	Lane08	14,400,000	11.9	KO	13,500,000	11.3	3,030,000	22.4	0	Sample
09	Lane09	17,400,000	14.6	WT	16,400,000	13.7	2,080,000	12.7	0	Sample
10	Lane10	15,200,000	12.7	KO	13,500,000	11.3	3,030,000	22.4	0	Sample

Chemi Channel

Image Name: ERT2 Cohort 4 - ingWAT Slit3 (reprobe)

Lane Detection: Manual

Protein: Slit3

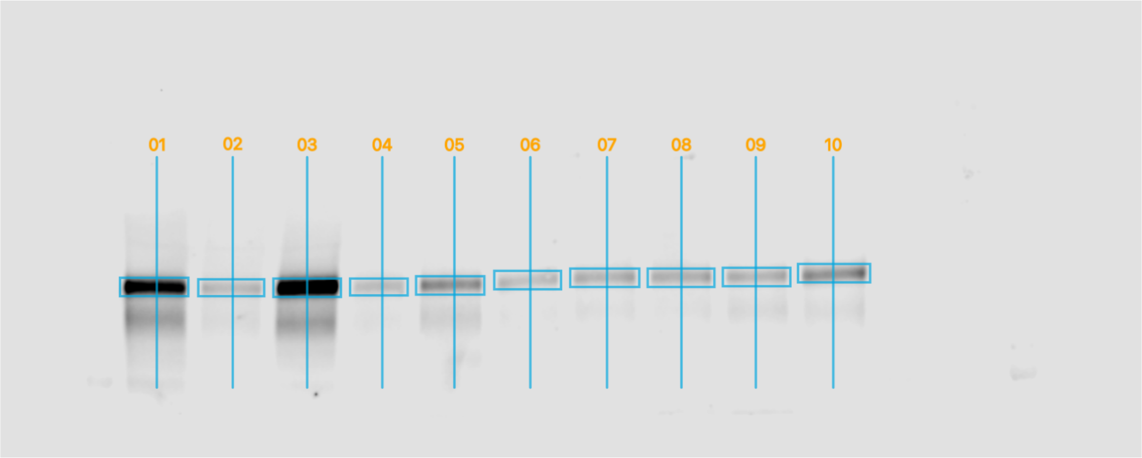

Protein Analysis Table

Lane	Name	MW	Normalized Signal	SNR	Replicate	Avg. Norm. Signal	Avg. SNR	Std. Dev.	% CV	Treatment (%)	Type
01	Lane01		39.5	7.36	WT	25.2	7.22	20.3	80.6	0	Sample
02	Lane02		6.18	4.95	KO	10.4	7.11	3.97	38.1	0	Sample
03	Lane03		53.8	10.3	WT	25.2	7.22	20.3	80.6	0	Sample
04	Lane04		10.0	6.13	KO	10.4	7.11	3.97	38.1	0	Sample
05	Lane05		13.4	5.89	WT	25.2	7.22	20.3	80.6	0	Sample
06	Lane06		8.93	6.81	KO	10.4	7.11	3.97	38.1	0	Sample
07	Lane07		11.6	6.81	WT	25.2	7.22	20.3	80.6	0	Sample
08	Lane08		10.0	7.69	KO	10.4	7.11	3.97	38.1	0	Sample
09	Lane09		7.69	5.69	WT	25.2	7.22	20.3	80.6	0	Sample
10	Lane10		16.9	9.98	KO	10.4	7.11	3.97	38.1	0	Sample

Figure 3g

Anti- Slit3

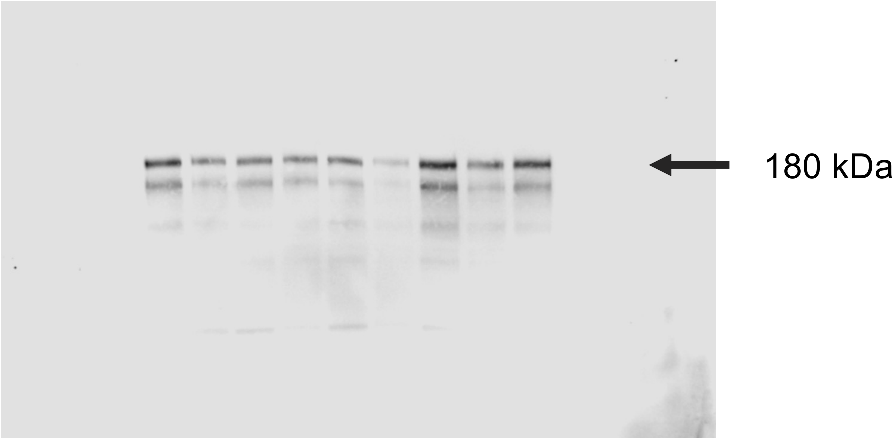

Anti- Vinculin

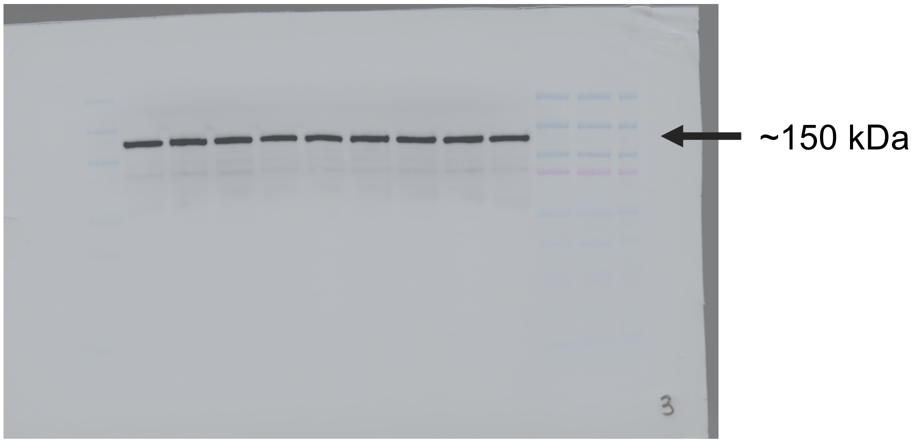

Total Protein

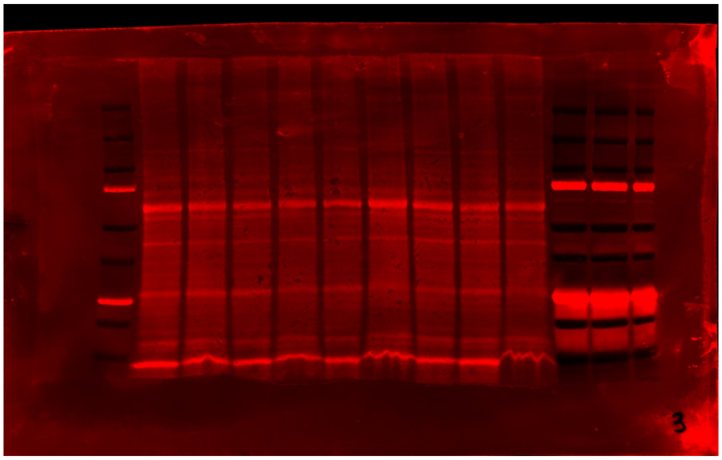

Experimental Analysis

520 Channel

Image Name: ERT2 Cohort 4 pgWAT total protein

Lane Detection: Automatic Lane Finding

Protein: Total Protein

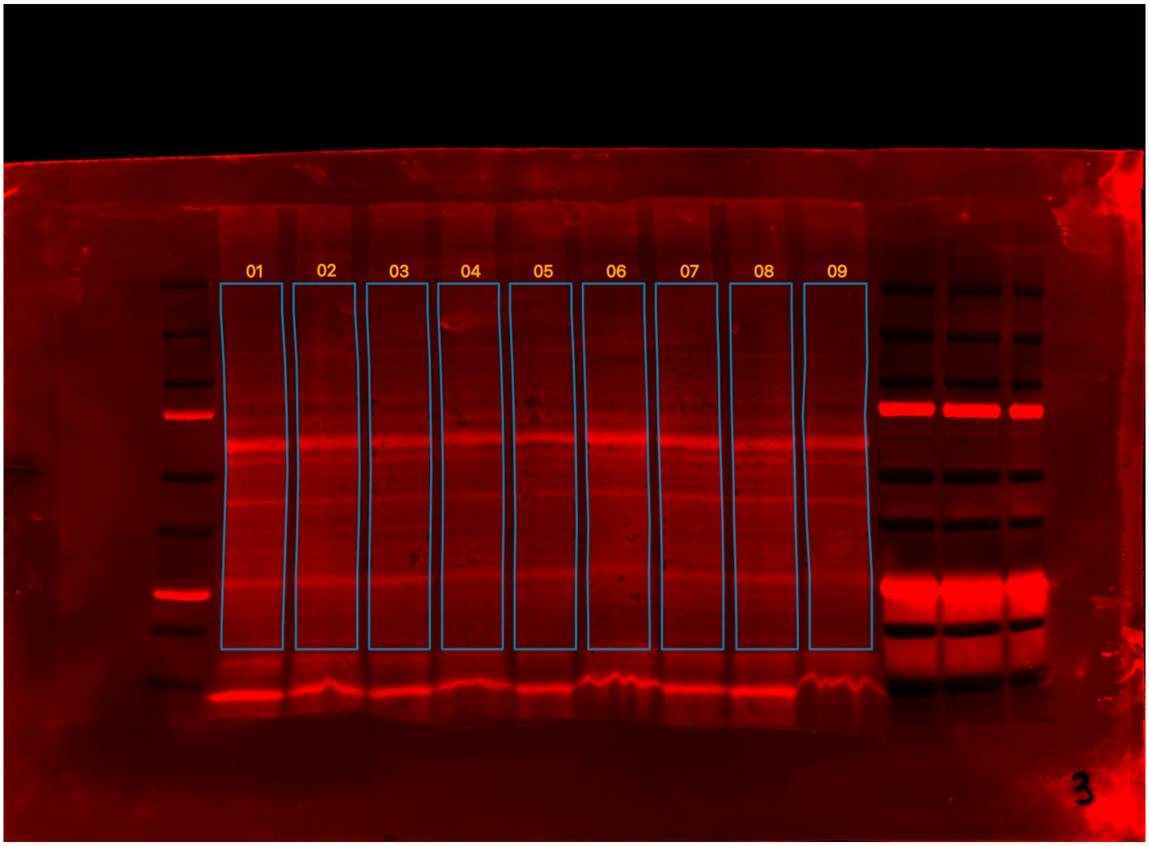

Total Protein Stain Analysis Table

Lane	Name	Signal	SNR	Replicate	Avg. Signal	Avg. SNR	Std. Dev.	% CV	Treatment (%)	Type
01	Lane01	20,700,000	7.23	WT	15,600,000	5.46	3,330,000	21.3	0	Sample
02	Lane02	18,900,000	6.58	KO	16,900,000	5.91	2,220,000	13.1	0	Sample
03	Lane03	14,400,000	5.09	WT	15,600,000	5.46	3,330,000	21.3	0	Sample
04	Lane04	13,900,000	4.91	KO	16,900,000	5.91	2,220,000	13.1	0	Sample
05	Lane05	12,900,000	4.50	WT	15,600,000	5.46	3,330,000	21.3	0	Sample
06	Lane06	18,200,000	6.36	KO	16,900,000	5.91	2,220,000	13.1	0	Sample
07	Lane07	17,200,000	5.99	WT	15,600,000	5.46	3,330,000	21.3	0	Sample
08	Lane08	16,500,000	5.77	KO	16,900,000	5.91	2,220,000	13.1	0	Sample
09	Lane09	13,000,000	4.47	WT	15,600,000	5.46	3,330,000	21.3	0	Sample

Chemi Channel

Image Name: ERT2 Cohort 4 - pgWAT Slit3 - no EPI

Lane Detection: Manual

Protein: Slit3

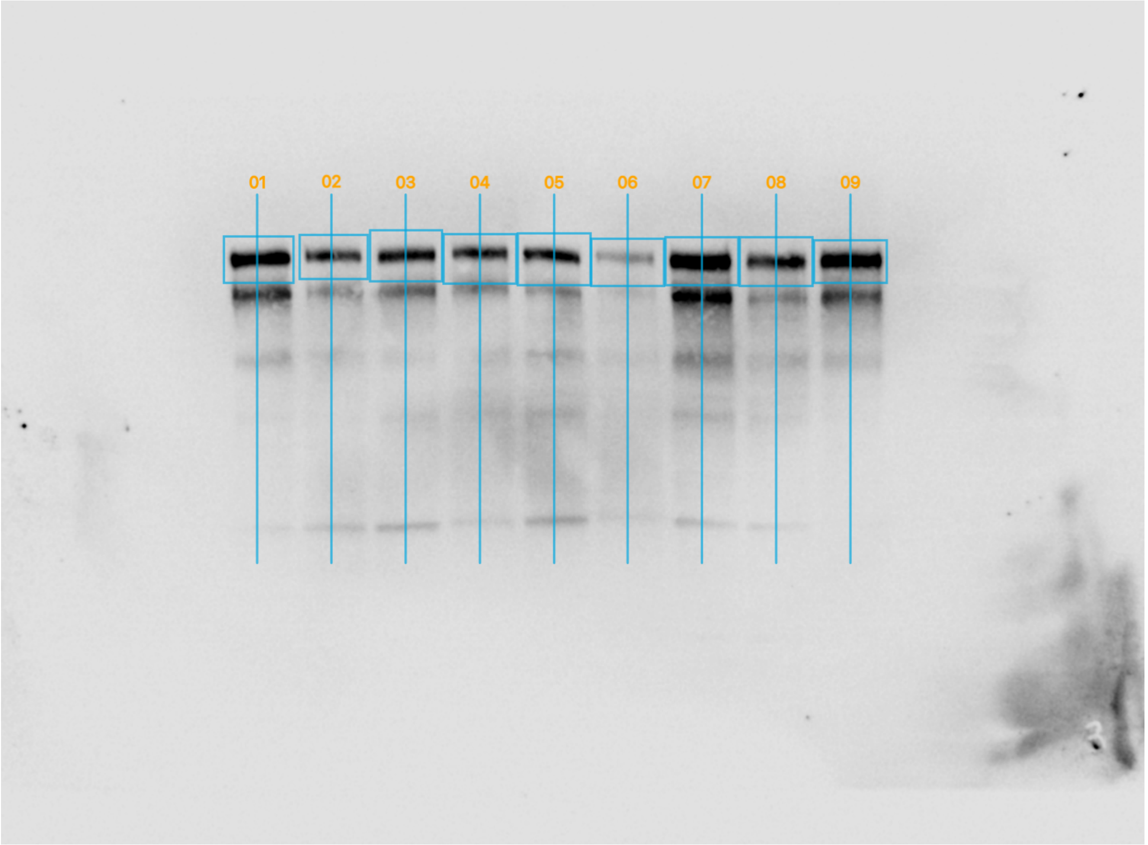

Protein Analysis Table

Lane	Name	MW	Normalized Signal	SNR	Replicate	Avg. Norm. Signal	Avg. SNR	Std. Dev.	% CV	Treatment (%)	Type
01	Lane01		1.52	4.93	WT	1.77	3.02	0.572	32.2	0	Sample
02	Lane02		0.733	2.53	KO	0.865	2.22	0.413	47.8	0	Sample
03	Lane03		1.39	3.05	WT	1.77	3.02	0.572	32.2	0	Sample
04	Lane04		1.29	3.29	KO	0.865	2.22	0.413	47.8	0	Sample
05	Lane05		1.27	1.95	WT	1.77	3.02	0.572	32.2	0	Sample
06	Lane06		0.351	1.39	KO	0.865	2.22	0.413	47.8	0	Sample
07	Lane07		2.04	2.26	WT	1.77	3.02	0.572	32.2	0	Sample
08	Lane08		1.08	1.68	KO	0.865	2.22	0.413	47.8	0	Sample
09	Lane09		2.65	2.92	WT	1.77	3.02	0.572	32.2	0	Sample

Figure 4b

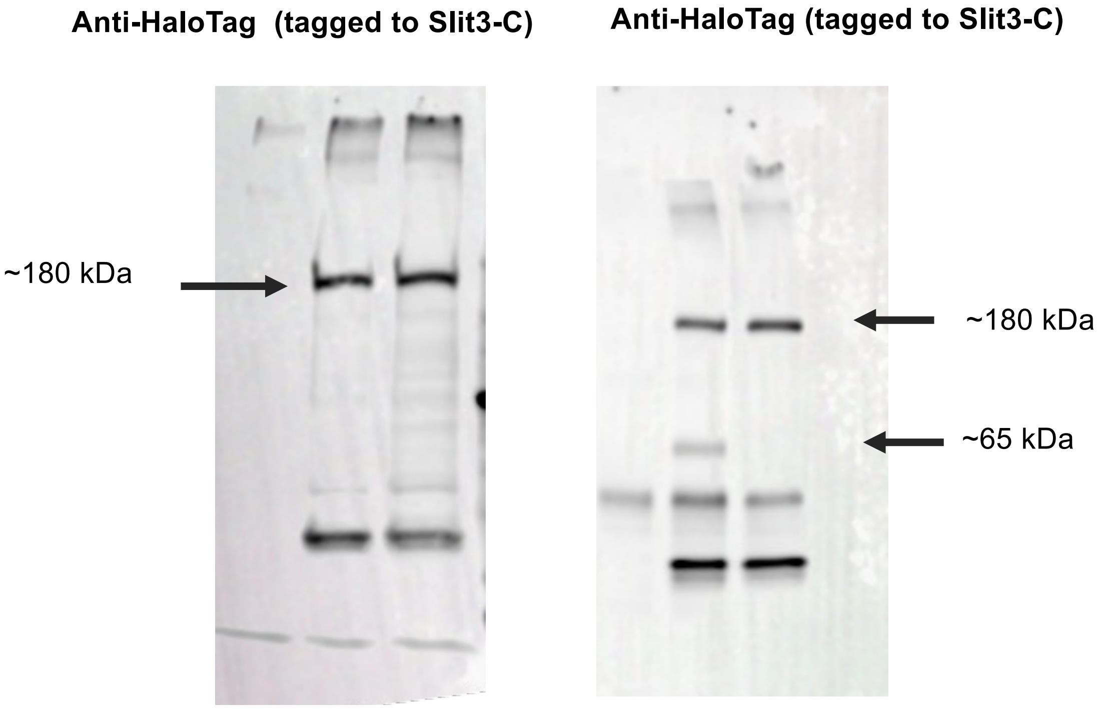

Figure 4c

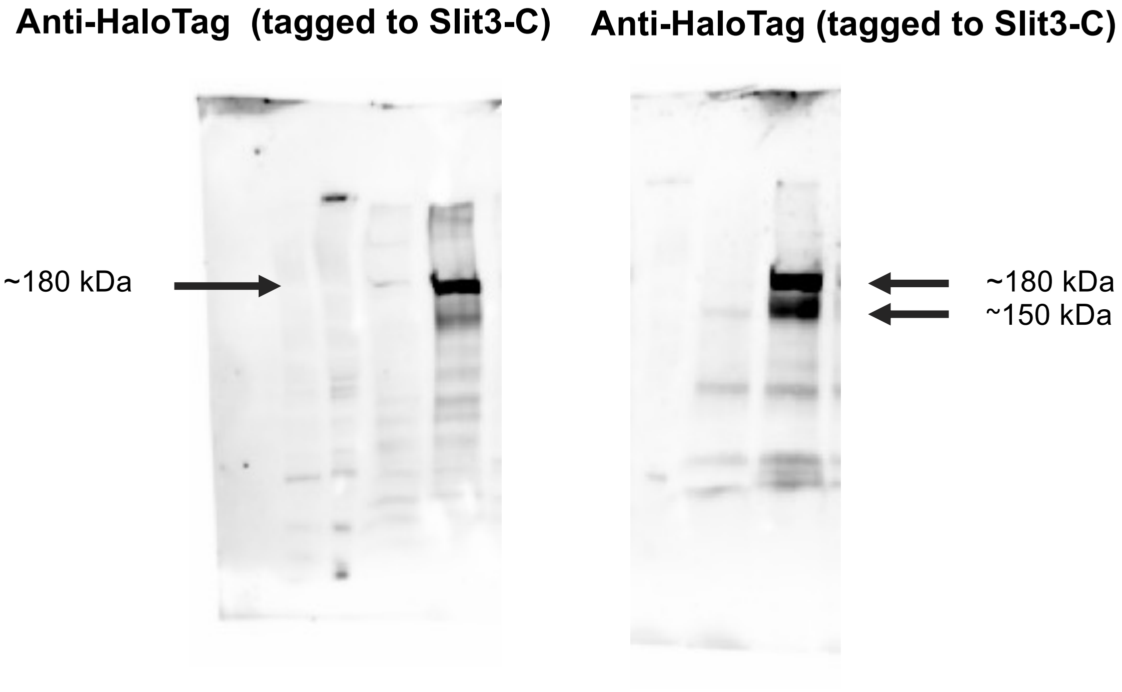

Figure 4f

Anti-Slit3

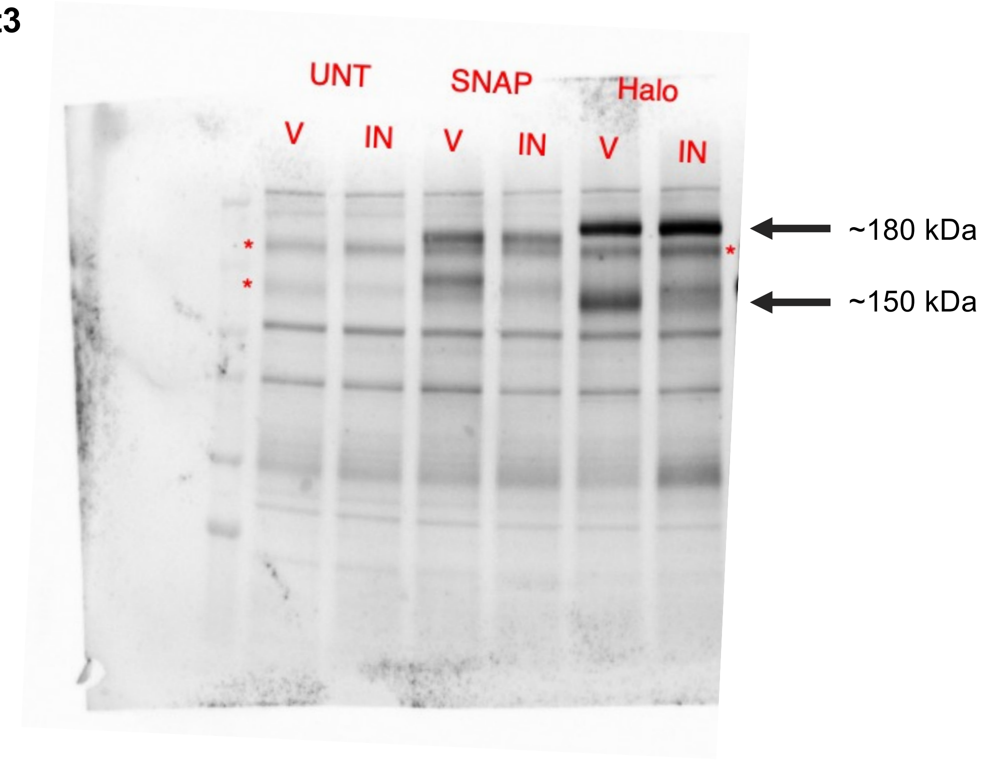

Figure 4g

Anti-SNAP tag

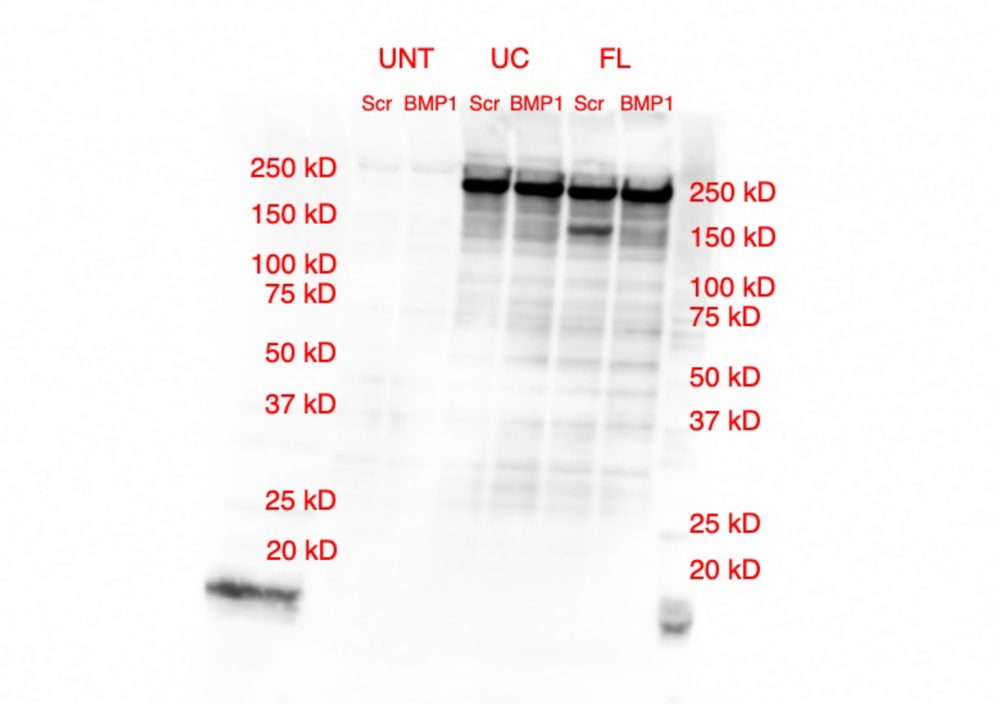

Figure 5i

Anti- TH

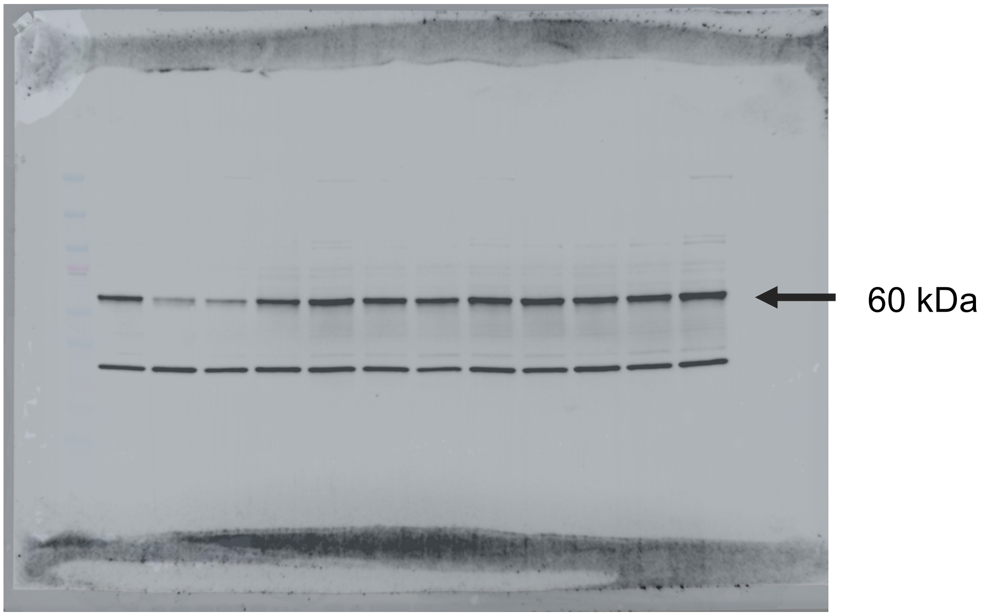

Total Protein

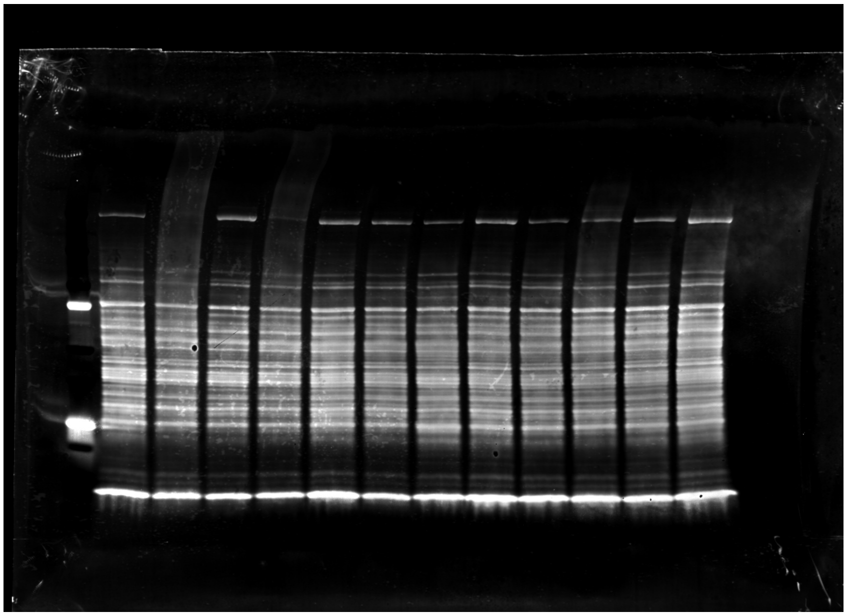

Experimental Analysis

520 Channel

Image Name: Dual - TH

Lane Detection: Automatic Lane Finding

Protein: Total Protein stain

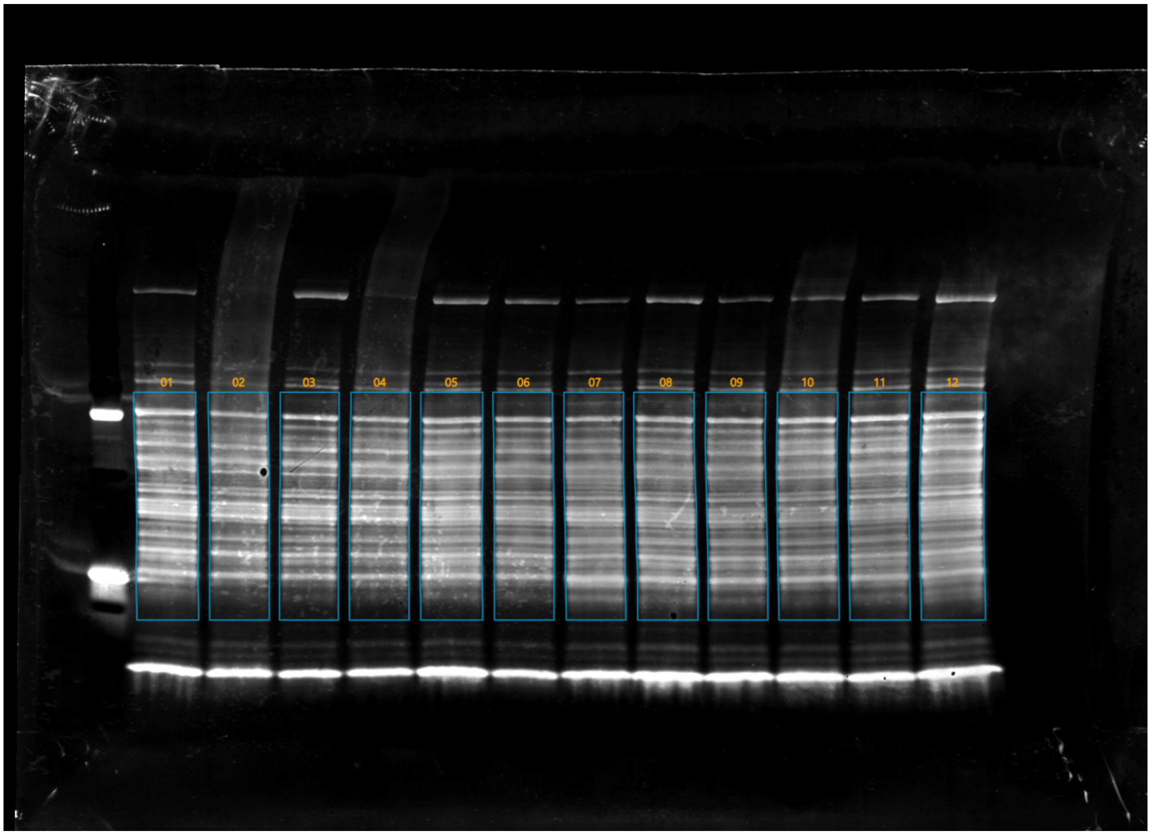

Total Protein Stain Analysis Table

Lane	Name	Signal	SNR	Replicate	Avg. Signal	Avg. SNR	Std. Dev.	% CV	Treatment (%)	Type
01	GFP	27,600,000	19.8	Rep-Grp-01-02-03-0...	22,400,000	16.5	2,920,000	13.0	0	Sample
02	GFP	22,600,000	16.9	Rep-Grp-01-02-03-0...	22,400,000	16.5	2,920,000	13.0	0	Sample
03	GFP	20,600,000	15.6	Rep-Grp-01-02-03-0...	22,400,000	16.5	2,920,000	13.0	0	Sample
04	GFP	21,800,000	16.3	Rep-Grp-01-02-03-0...	22,400,000	16.5	2,920,000	13.0	0	Sample
05	GFP	25,400,000	18.2	Rep-Grp-01-02-03-0...	22,400,000	16.5	2,920,000	13.0	0	Sample
06	GFP	19,200,000	14.3	Rep-Grp-01-02-03-0...	22,400,000	16.5	2,920,000	13.0	0	Sample
07	Slit3 FL	20,900,000	15.6	Rep-Grp-01-02-03-0...	22,400,000	16.5	2,920,000	13.0	0	Sample
08	Slit3 FL	21,900,000	16.1	Rep-Grp-01-02-03-0...	22,400,000	16.5	2,920,000	13.0	0	Sample
09	Slit3 FL	19,200,000	14.3	Rep-Grp-01-02-03-0...	22,400,000	16.5	2,920,000	13.0	0	Sample
10	Slit3 FL	20,600,000	15.2	Rep-Grp-01-02-03-0...	22,400,000	16.5	2,920,000	13.0	0	Sample
11	Slit3 FL	21,800,000	16.1	Rep-Grp-01-02-03-0...	22,400,000	16.5	2,920,000	13.0	0	Sample
12	Slit3 FL	27,700,000	19.2	Rep-Grp-01-02-03-0...	22,400,000	16.5	2,920,000	13.0	0	Sample

Chemi Channel

Image Name: Dual - TH

Lane Detection: Automatic Lane Finding

Protein: Slit3 FL

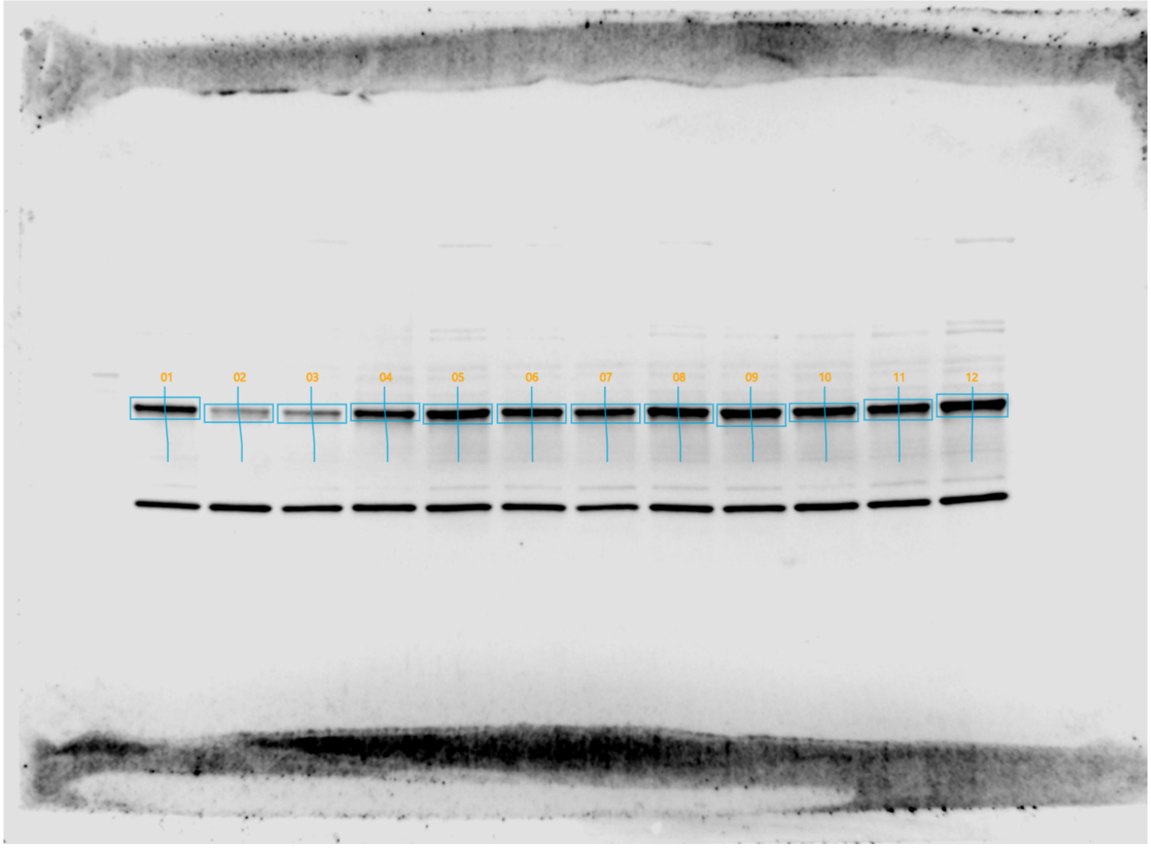

Protein Analysis Table

Lane	Name	MW	Normalized Signal	SNR	Replicate	Avg. Norm. Signal	Avg. SNR	Std. Dev.	% CV	Treatment (%)	Type
01	GFP		2.45	18.8	Rep-Grp-01-02-03...	3.64	15.1	1.42	39.2	0	Sample
02	GFP		1.10	11.1	Rep-Grp-01-02-03...	3.64	15.1	1.42	39.2	0	Sample
03	GFP		1.29	9.72	Rep-Grp-01-02-03...	3.64	15.1	1.42	39.2	0	Sample
04	GFP		2.92	20.2	Rep-Grp-01-02-03...	3.64	15.1	1.42	39.2	0	Sample
05	GFP		4.20	16.7	Rep-Grp-01-02-03...	3.64	15.1	1.42	39.2	0	Sample
06	GFP		4.47	16.7	Rep-Grp-01-02-03...	3.64	15.1	1.42	39.2	0	Sample
07	Slit3 FL		3.40	12.5	Rep-Grp-01-02-03...	3.64	15.1	1.42	39.2	0	Sample
08	Slit3 FL		4.53	15.8	Rep-Grp-01-02-03...	3.64	15.1	1.42	39.2	0	Sample
09	Slit3 FL		5.62	13.2	Rep-Grp-01-02-03...	3.64	15.1	1.42	39.2	0	Sample
10	Slit3 FL		4.57	15.7	Rep-Grp-01-02-03...	3.64	15.1	1.42	39.2	0	Sample
11	Slit3 FL		4.32	14.2	Rep-Grp-01-02-03...	3.64	15.1	1.42	39.2	0	Sample
12	Slit3 FL		4.77	16.8	Rep-Grp-01-02-03...	3.64	15.1	1.42	39.2	0	Sample

Figure 5i

Anti - UCP1

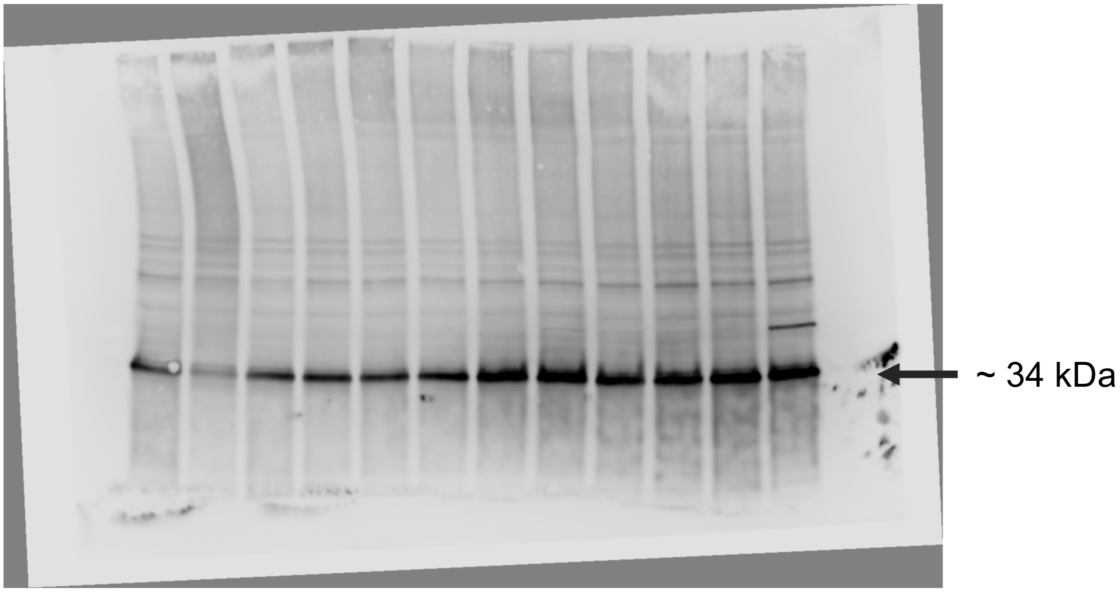

Total Protein

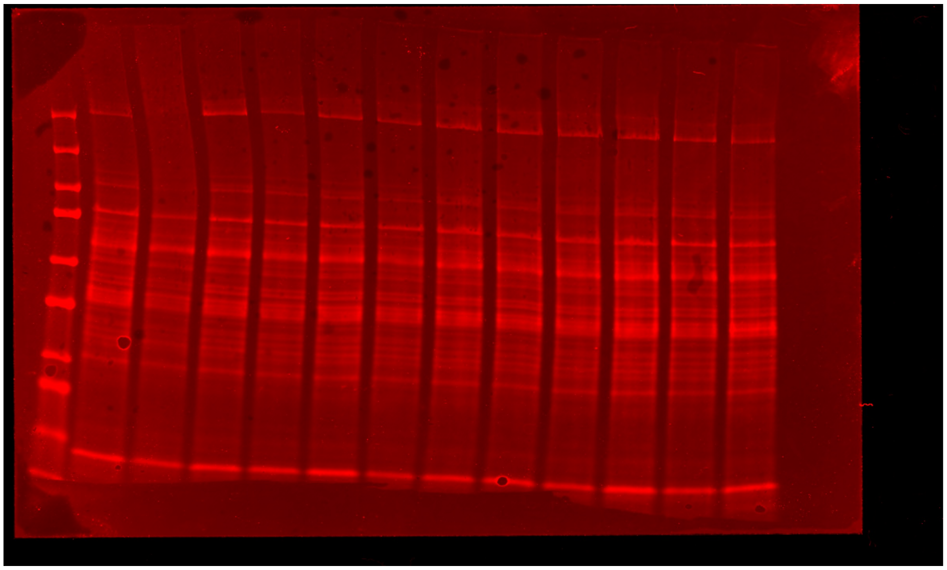

Experimental Analysis

700 Channel

Image Name: TotalProtein GFPxFL

Lane Detection: Automatic Lane Finding

Protein: Total Protein

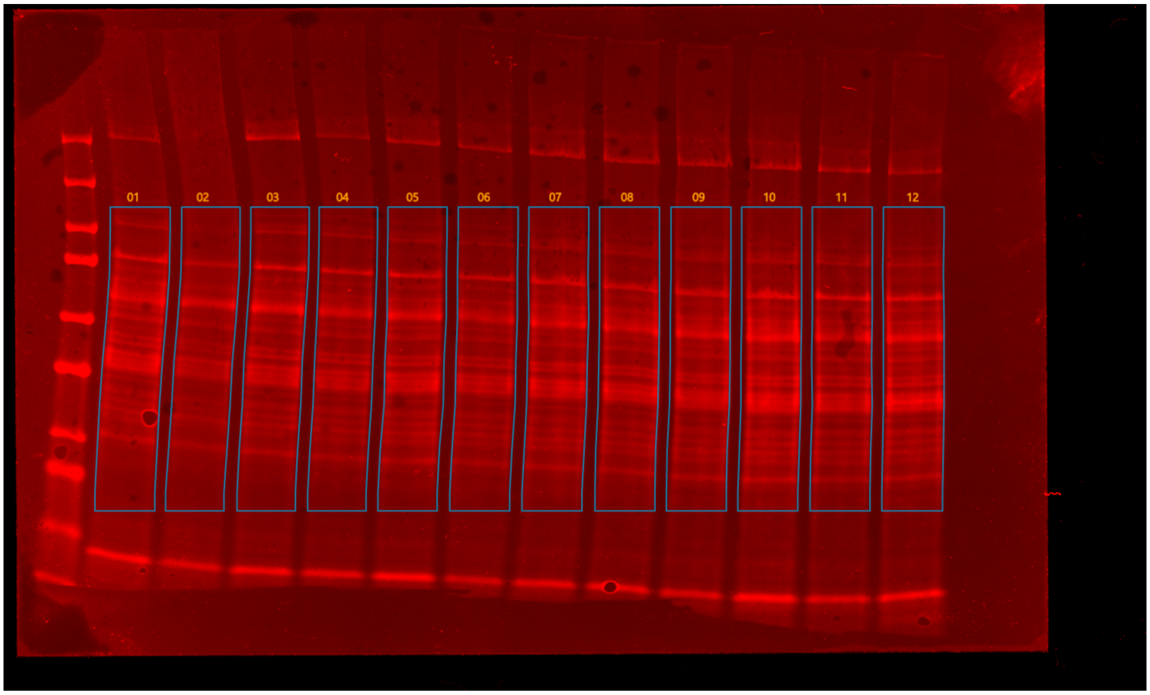

Total Protein Stain Analysis Table

Lane	Name	Signal	SNR	Replicate	Avg. Signal	Avg. SNR	Std. Dev.	% CV	Treatment (%)	Type
01	GFP	141,000	19.1	Rep-Grp-01-02-03-0...	115,000	15.6	13,600	11.9	0	Sample
02	GFP	103,000	14.3	Rep-Grp-01-02-03-0...	115,000	15.6	13,600	11.9	0	Sample
03	GFP	111,000	15.5	Rep-Grp-01-02-03-0...	115,000	15.6	13,600	11.9	0	Sample
04	GFP	98,000	13.6	Rep-Grp-01-02-03-0...	115,000	15.6	13,600	11.9	0	Sample
05	GFP	113,000	15.5	Rep-Grp-01-02-03-0...	115,000	15.6	13,600	11.9	0	Sample
06	GFP	98,500	13.3	Rep-Grp-01-02-03-0...	115,000	15.6	13,600	11.9	0	Sample
07	Slit3 FL	119,000	16.1	Rep-Grp-01-02-03-0...	115,000	15.6	13,600	11.9	0	Sample
08	Slit3 FL	112,000	15.2	Rep-Grp-01-02-03-0...	115,000	15.6	13,600	11.9	0	Sample
09	Slit3 FL	114,000	15.4	Rep-Grp-01-02-03-0...	115,000	15.6	13,600	11.9	0	Sample
10	Slit3 FL	135,000	18.4	Rep-Grp-01-02-03-0...	115,000	15.6	13,600	11.9	0	Sample
11	Slit3 FL	107,000	14.5	Rep-Grp-01-02-03-0...	115,000	15.6	13,600	11.9	0	Sample
12	Slit3 FL	127,000	17.0	Rep-Grp-01-02-03-0...	115,000	15.6	13,600	11.9	0	Sample

Chemi Channel

Image Name: Ucp1andTH-Slit36FL

Lane Detection: Automatic Lane Finding

Protein: UCP1

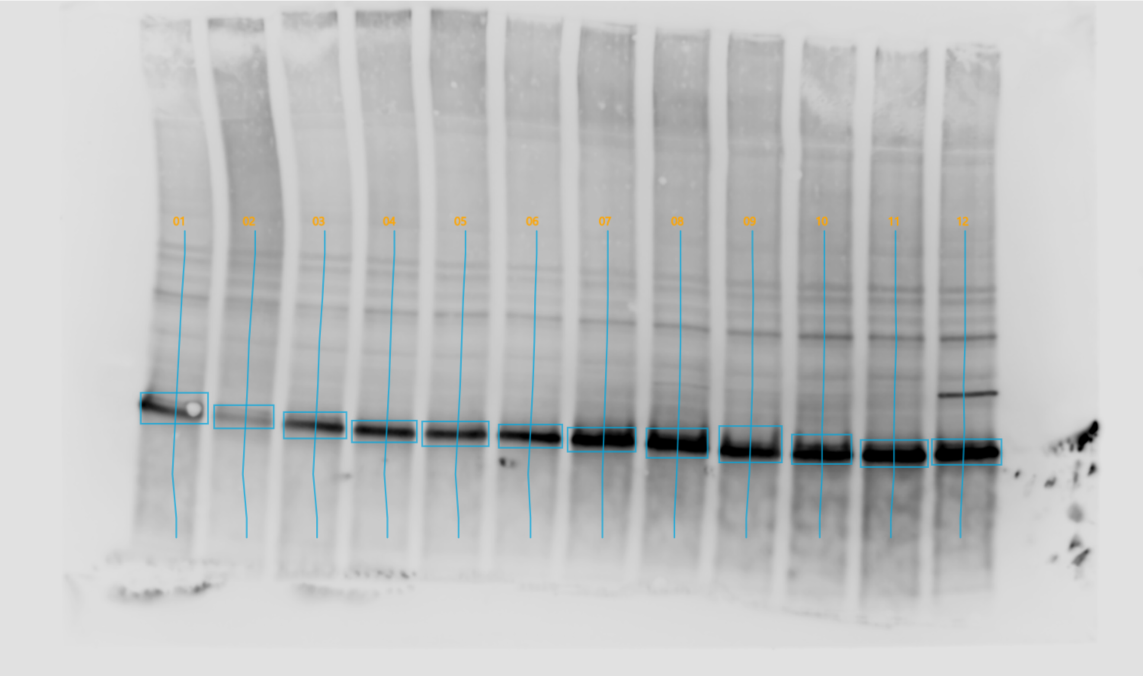

Protein Analysis Table

Lane	Name	MW	Normalized Signal	SNR	Replicate	Avg. Norm. Signal	Avg. SNR	Std. Dev.	% CV	Treatment (%)	Type
01	GFP		35.8	4.53	Rep-Grp-01-02-03...	62.0	5.80	21.1	34.1	0	Sample
02	GFP		20.3	2.95	Rep-Grp-01-02-03...	62.0	5.80	21.1	34.1	0	Sample
03	GFP		48.2	6.81	Rep-Grp-01-02-03...	62.0	5.80	21.1	34.1	0	Sample
04	GFP		59.2	7.75	Rep-Grp-01-02-03...	62.0	5.80	21.1	34.1	0	Sample
05	GFP		52.0	6.23	Rep-Grp-01-02-03...	62.0	5.80	21.1	34.1	0	Sample
06	GFP		66.7	6.82	Rep-Grp-01-02-03...	62.0	5.80	21.1	34.1	0	Sample
07	Slit3 FL		76.2	5.88	Rep-Grp-01-02-03...	62.0	5.80	21.1	34.1	0	Sample
08	Slit3 FL		83.7	5.46	Rep-Grp-01-02-03...	62.0	5.80	21.1	34.1	0	Sample
09	Slit3 FL		74.9	5.48	Rep-Grp-01-02-03...	62.0	5.80	21.1	34.1	0	Sample
10	Slit3 FL		53.7	5.41	Rep-Grp-01-02-03...	62.0	5.80	21.1	34.1	0	Sample
11	Slit3 FL		91.4	6.32	Rep-Grp-01-02-03...	62.0	5.80	21.1	34.1	0	Sample
12	Slit3 FL		81.9	5.96	Rep-Grp-01-02-03...	62.0	5.80	21.1	34.1	0	Sample

Figure 5I

Anti- TH

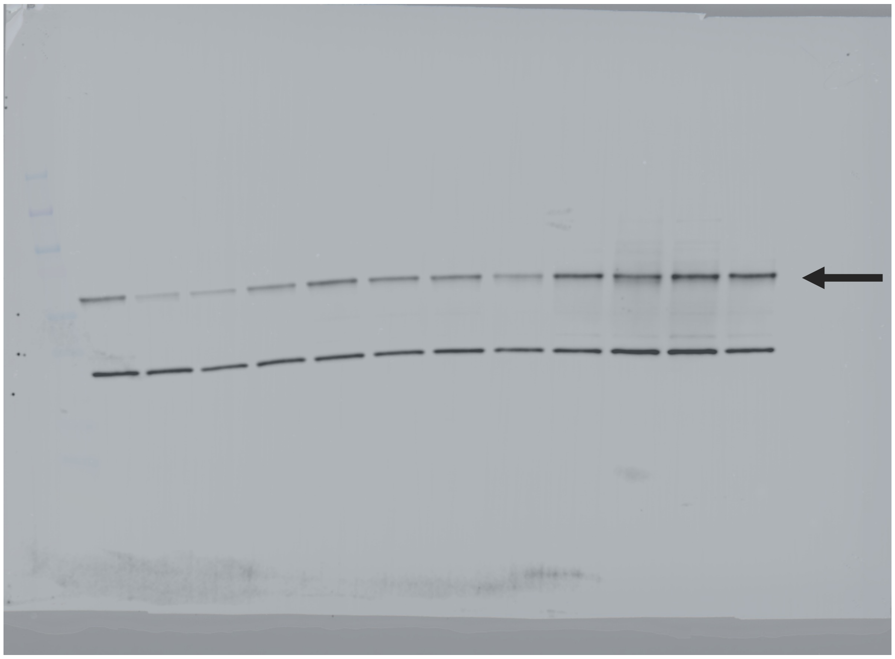

60 kDa

Total Protein

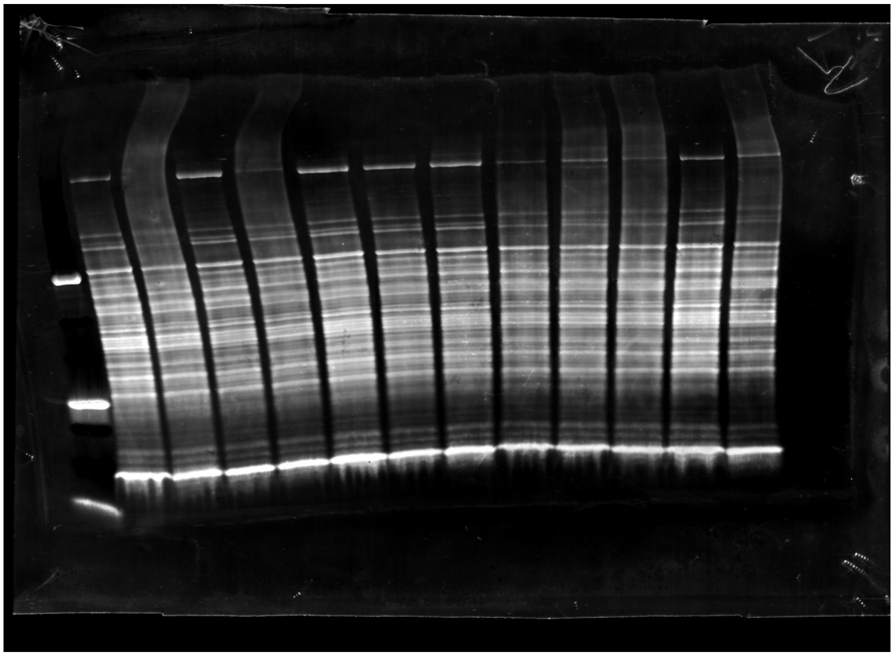

Experimental Analysis

520 Channel

Image Name: Kali - TH

Lane Detection: Automatic Lane Finding

Protein: Total Protein

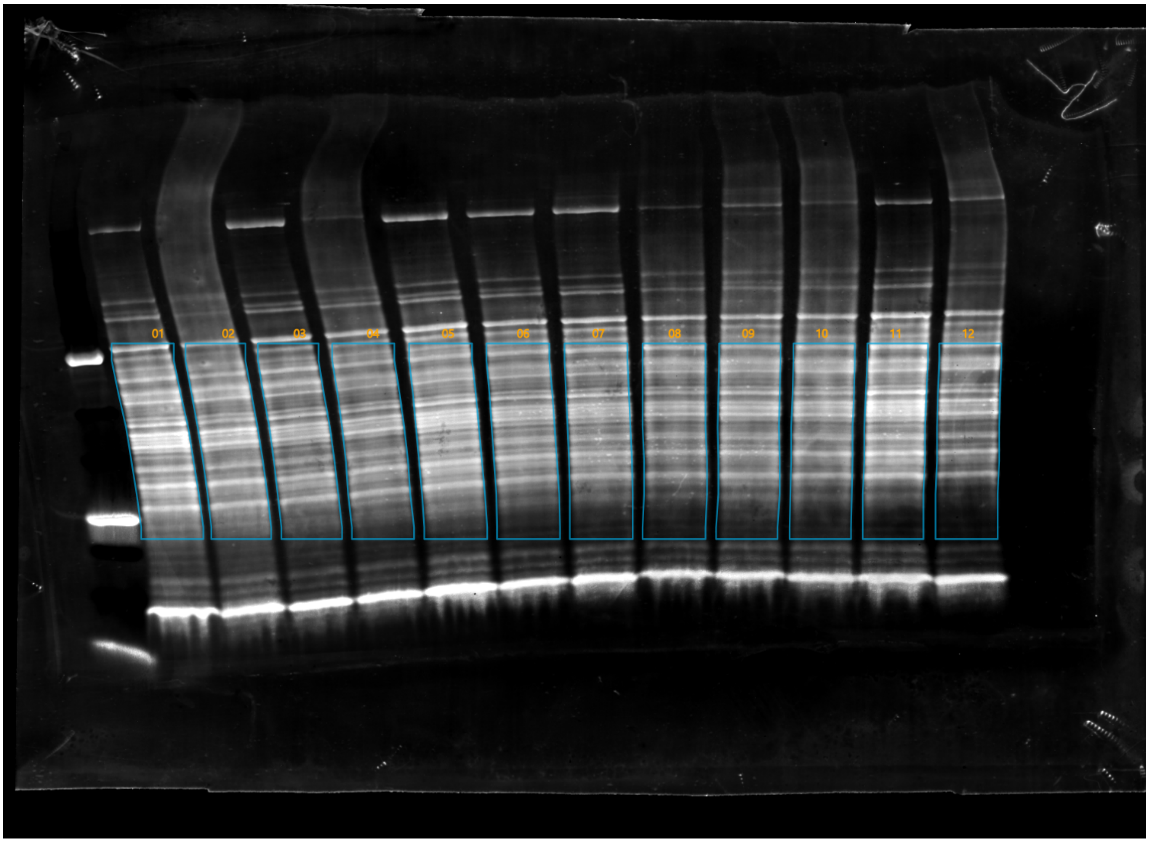

Total Protein Stain Analysis Table

Lane	Name	Signal	SNR	Replicate	Avg. Signal	Avg. SNR	Std. Dev.	% CV	Treatment (%)	Type
01	GFP	26,600,000	18.3	Rep-Grp-01-02-03-0...	20,900,000	14.5	3,040,000	14.5	0	Sample
02	GFP	23,500,000	16.8	Rep-Grp-01-02-03-0...	20,900,000	14.5	3,040,000	14.5	0	Sample
03	GFP	21,000,000	14.8	Rep-Grp-01-02-03-0...	20,900,000	14.5	3,040,000	14.5	0	Sample
04	GFP	20,400,000	14.0	Rep-Grp-01-02-03-0...	20,900,000	14.5	3,040,000	14.5	0	Sample
05	GFP5	24,900,000	16.9	Rep-Grp-01-02-03-0...	20,900,000	14.5	3,040,000	14.5	0	Sample
06	GFP	20,300,000	13.8	Rep-Grp-01-02-03-0...	20,900,000	14.5	3,040,000	14.5	0	Sample
07	Slit3 N	19,900,000	13.5	Rep-Grp-01-02-03-0...	20,900,000	14.5	3,040,000	14.5	0	Sample
08	Slit3 N	17,700,000	12.0	Rep-Grp-01-02-03-0...	20,900,000	14.5	3,040,000	14.5	0	Sample
09	Slit3 N	19,400,000	13.5	Rep-Grp-01-02-03-0...	20,900,000	14.5	3,040,000	14.5	0	Sample
10	Slit3 N	18,900,000	13.1	Rep-Grp-01-02-03-0...	20,900,000	14.5	3,040,000	14.5	0	Sample
11	Slit3 N	22,300,000	15.7	Rep-Grp-01-02-03-0...	20,900,000	14.5	3,040,000	14.5	0	Sample
12	Slit3 N	15,900,000	10.9	Rep-Grp-01-02-03-0...	20,900,000	14.5	3,040,000	14.5	0	Sample

Chemi Channel

Image Name: Kali - TH

Lane Detection: Automatic Lane Finding

Protein: TH

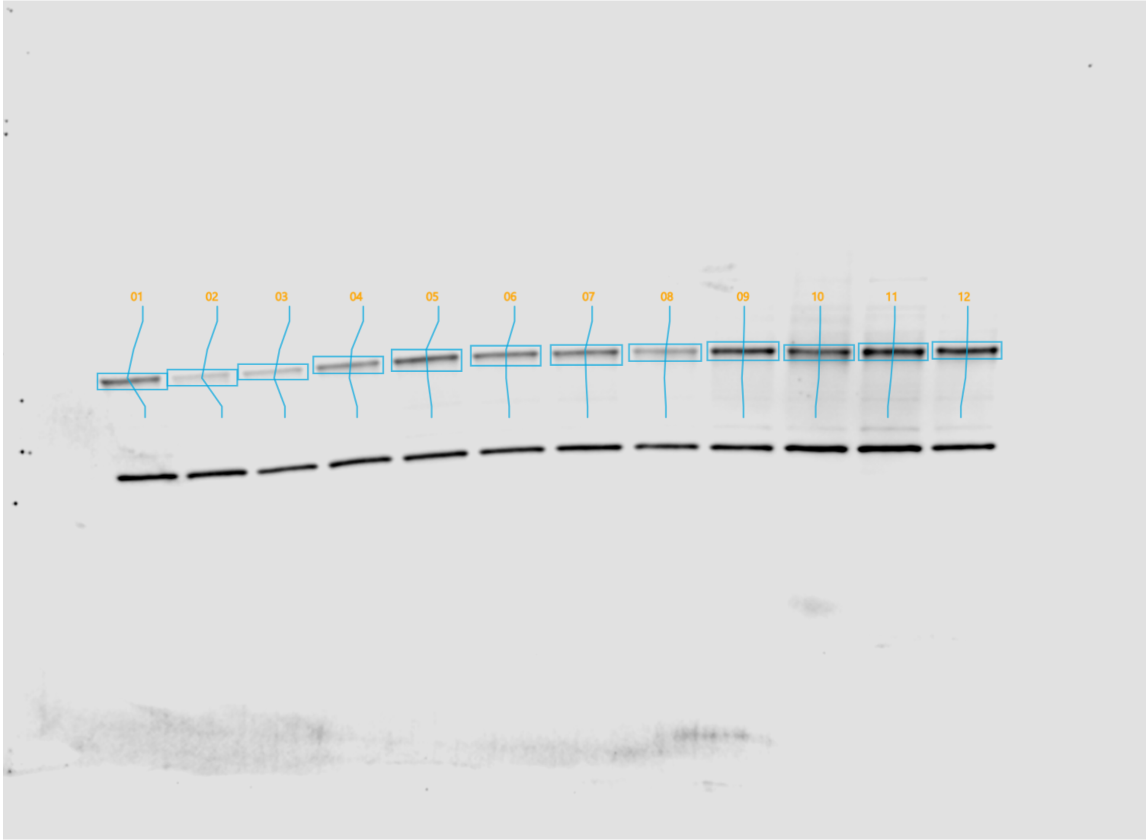

Protein Analysis Table

Lane	Name	MW	Normalized Signal	SNR	Replicate	Avg. Norm. Signal	Avg. SNR	Std. Dev.	% CV	Treatment (%)	Type
01	GFP		2.13	7.57	Rep-Grp-01-02-03...	3.30	9.65	1.82	55.1	0	Sample
02	GFP		0.844	4.47	Rep-Grp-01-02-03...	3.30	9.65	1.82	55.1	0	Sample
03	GFP		0.993	6.62	Rep-Grp-01-02-03...	3.30	9.65	1.82	55.1	0	Sample
04	GFP		2.23	10.7	Rep-Grp-01-02-03...	3.30	9.65	1.82	55.1	0	Sample
05	GFP5		2.97	9.79	Rep-Grp-01-02-03...	3.30	9.65	1.82	55.1	0	Sample
06	GFP		2.84	8.62	Rep-Grp-01-02-03...	3.30	9.65	1.82	55.1	0	Sample
07	Slit3 N		3.22	9.02	Rep-Grp-01-02-03...	3.30	9.65	1.82	55.1	0	Sample
08	Slit3 N		2.35	8.50	Rep-Grp-01-02-03...	3.30	9.65	1.82	55.1	0	Sample
09	Slit3 N		5.01	14.2	Rep-Grp-01-02-03...	3.30	9.65	1.82	55.1	0	Sample
10	Slit3 N		5.09	12.4	Rep-Grp-01-02-03...	3.30	9.65	1.82	55.1	0	Sample
11	Slit3 N		5.28	10.5	Rep-Grp-01-02-03...	3.30	9.65	1.82	55.1	0	Sample
12	Slit3 N		6.64	13.5	Rep-Grp-01-02-03...	3.30	9.65	1.82	55.1	0	Sample

Figure 5I

Anti- UCP1

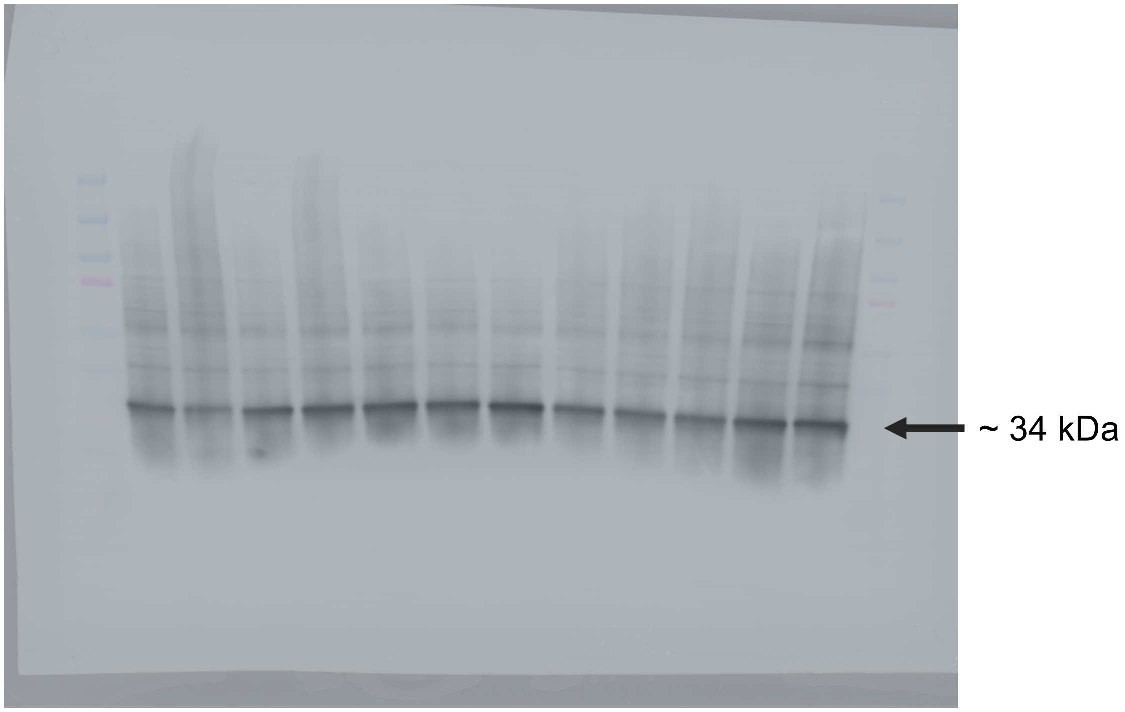

Total Protein

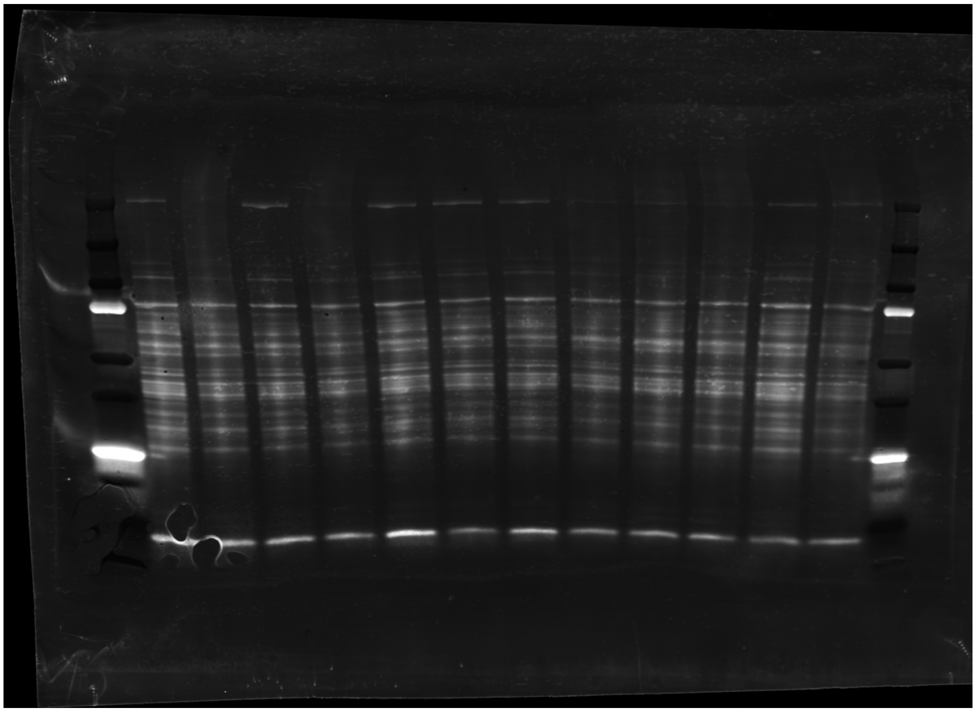

Experimental Analysis

520 Channel

Image Name: Dual - UCP1

Lane Detection: Automatic Lane Finding

Protein: Total Protein stain

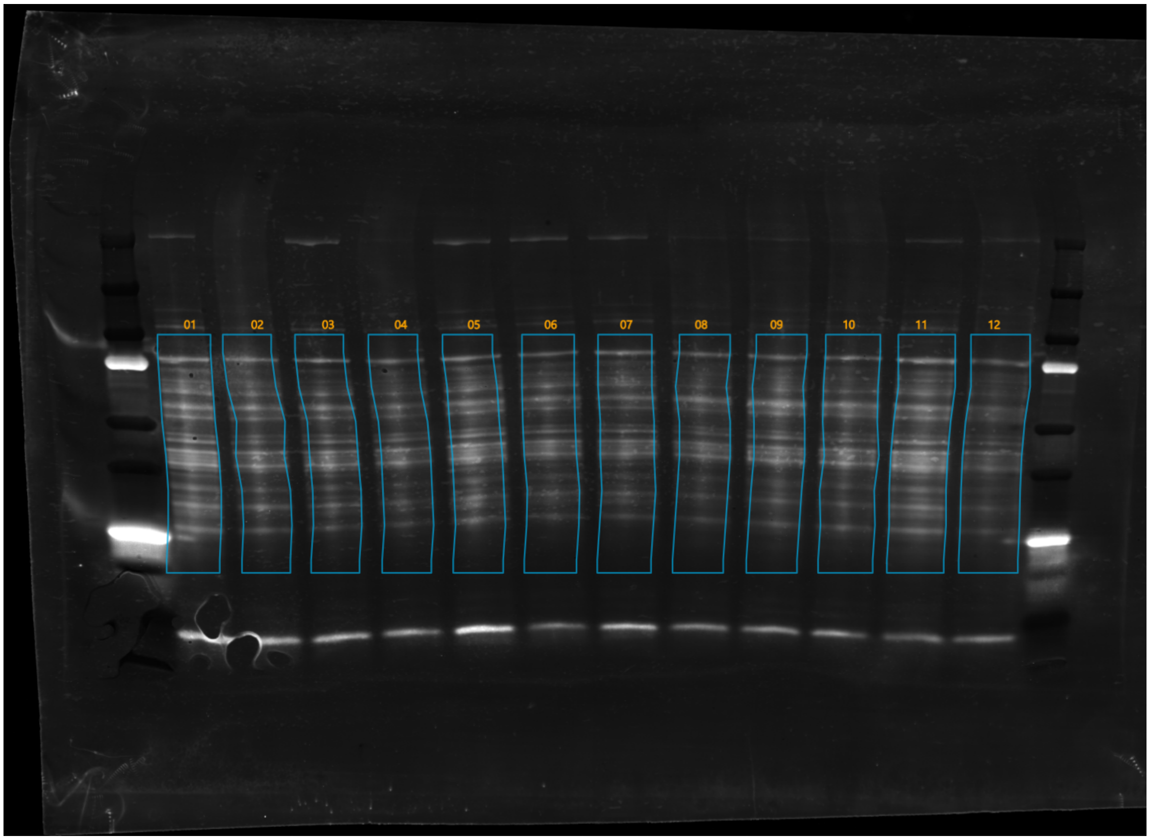

Total Protein Stain Analysis Table

Lane	Name	Signal	SNR	Replicate	Avg. Signal	Avg. SNR	Std. Dev.	% CV	Treatment (%)	Type
01	Lane01	14,800,000	11.4	GFP	8,760,000	7.10	3,280,000	37.4	0	Sample
02	Lane02	8,930,000	7.54	GFP	8,760,000	7.10	3,280,000	37.4	0	Sample
03	Lane03	7,010,000	5.92	GFP	8,760,000	7.10	3,280,000	37.4	0	Sample
04	Lane04	5,860,000	4.87	GFP	8,760,000	7.10	3,280,000	37.4	0	Sample
05	Lane05	9,470,000	7.75	GFP	8,760,000	7.10	3,280,000	37.4	0	Sample
06	Lane06	6,480,000	5.07	GFP	8,760,000	7.10	3,280,000	37.4	0	Sample
07	Lane07	6,790,000	5.16	N terminal	8,420,000	6.32	2,710,000	32.2	0	Sample
08	Lane08	5,960,000	4.81	N terminal	8,420,000	6.32	2,710,000	32.2	0	Sample
09	Lane09	8,500,000	6.85	N terminal	8,420,000	6.32	2,710,000	32.2	0	Sample
10	Lane10	8,760,000	6.56	N terminal	8,420,000	6.32	2,710,000	32.2	0	Sample
11	Lane11	13,500,000	9.72	N terminal	8,420,000	6.32	2,710,000	32.2	0	Sample
12	Lane12	6,990,000	4.83	N terminal	8,420,000	6.32	2,710,000	32.2	0	Sample

Chemi Channel

Image Name: Dual - UCP1

Lane Detection: Automatic Lane Finding

Protein: Ucp1

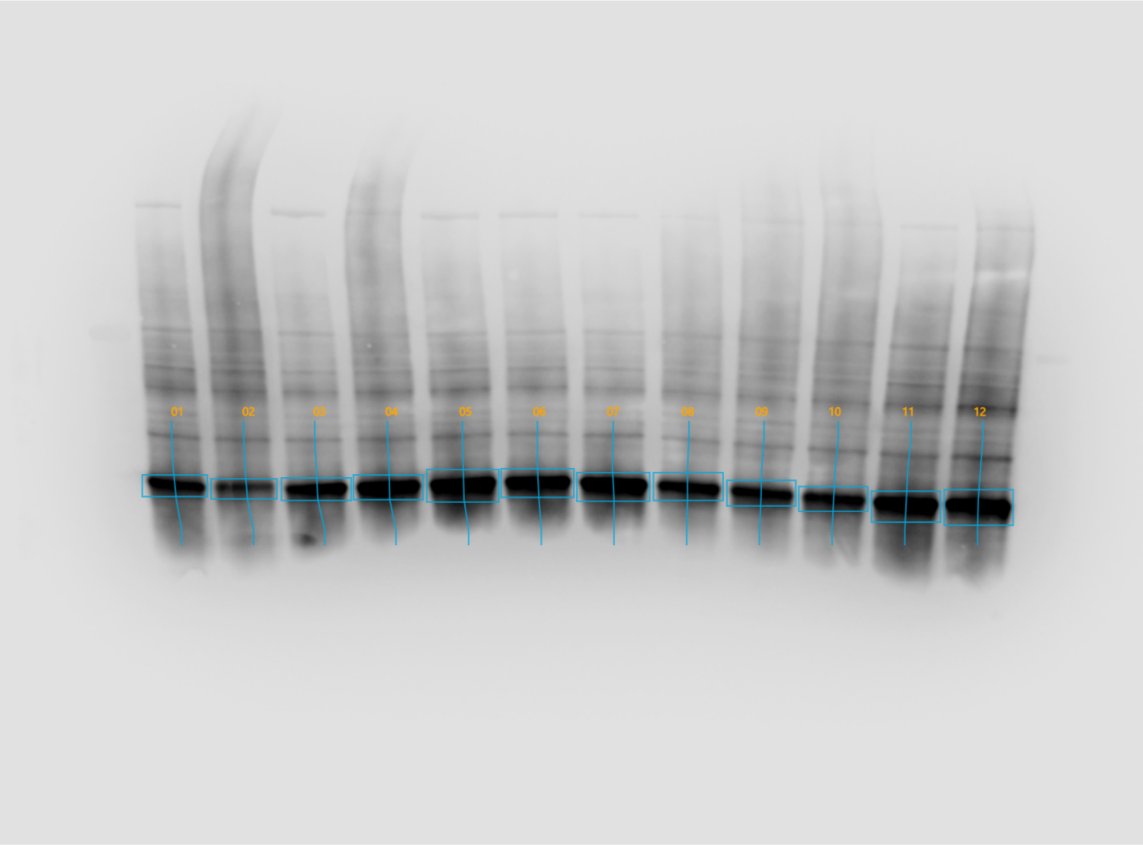

Protein Analysis Table

Lane	Name	MW	Normalized Signal	SNR	Replicate	Avg. Norm. Signal	Avg. SNR	Std. Dev.	% CV	Treatment (%)	Type
01	Lane01		38.4	1.78	GFP	111	2.55	58.1	52.3	0	Sample
02	Lane02		46.3	1.83	GFP	111	2.55	58.1	52.3	0	Sample
03	Lane03		111	3.16	GFP	111	2.55	58.1	52.3	0	Sample
04	Lane04		170	3.28	GFP	111	2.55	58.1	52.3	0	Sample
05	Lane05		130	2.30	GFP	111	2.55	58.1	52.3	0	Sample
06	Lane06		171	2.94	GFP	111	2.55	58.1	52.3	0	Sample
07	Lane07		196	3.57	N terminal	128	2.88	49.1	38.5	0	Sample
08	Lane08		149	3.40	N terminal	128	2.88	49.1	38.5	0	Sample
09	Lane09		94.0	3.13	N terminal	128	2.88	49.1	38.5	0	Sample
10	Lane10		83.5	2.58	N terminal	128	2.88	49.1	38.5	0	Sample
11	Lane11		78.2	2.40	N terminal	128	2.88	49.1	38.5	0	Sample
12	Lane12		166	2.22	N terminal	128	2.88	49.1	38.5	0	Sample

Figure 5o

Anti- TH

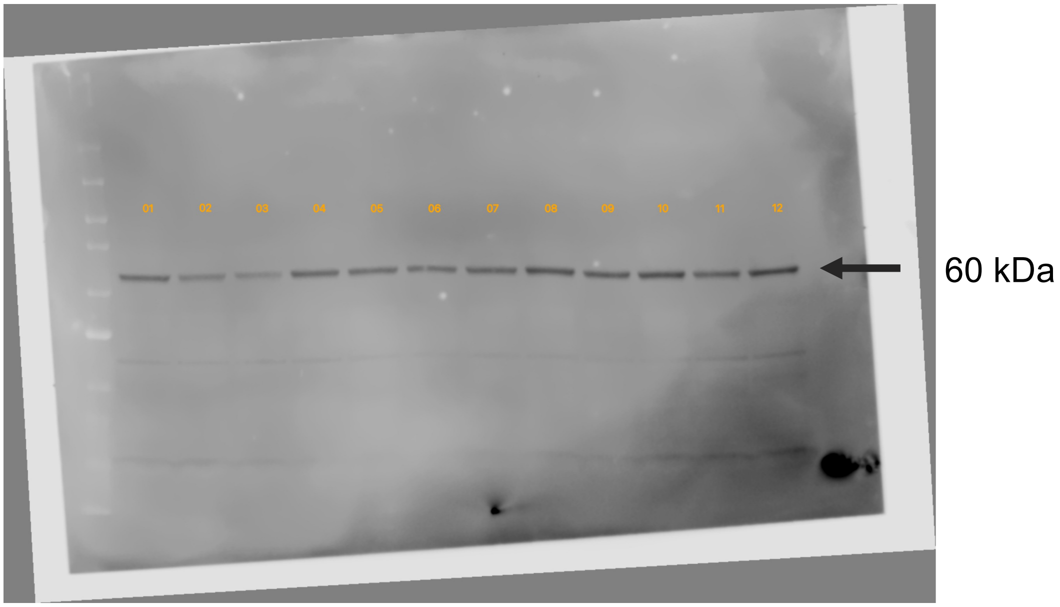

Total Protein

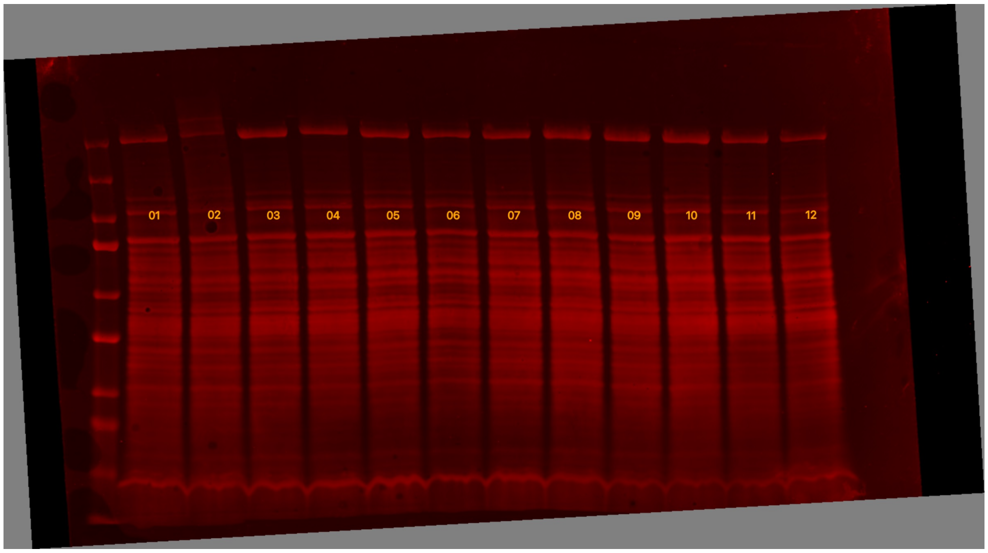

Experimental Analysis

700 Channel

Image Name: Total protein stain for TH in SLIT3-C OE

Lane Detection: Automatic Lane Finding

Protein: Total Protein

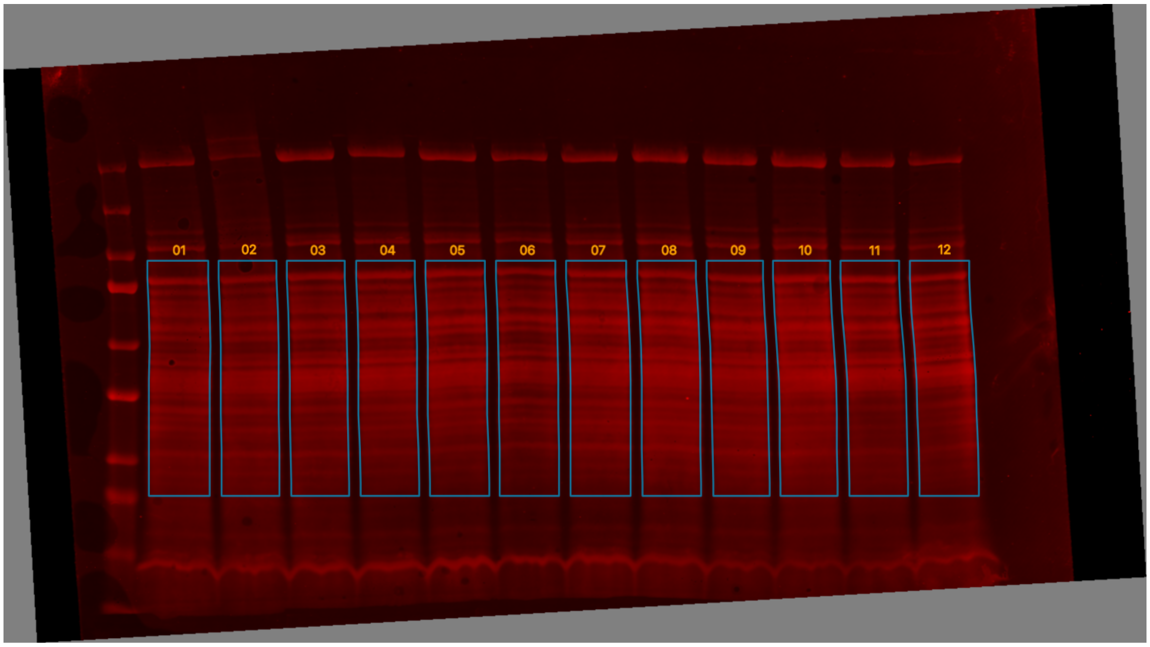

Total Protein Stain Analysis Table

Lane	Name	Signal	SNR	Replicate	Avg. Signal	Avg. SNR	Std. Dev.	% CV	Treatment (%)	Type
01	Lane01	294,000	29.0	GFP	283,000	28.8	17,100	6.03	0	Sample
02	Lane02	275,000	28.5	GFP	283,000	28.8	17,100	6.03	0	Sample
03	Lane03	281,000	29.1	GFP	283,000	28.8	17,100	6.03	0	Sample
04	Lane04	293,000	29.9	GFP	283,000	28.8	17,100	6.03	0	Sample
05	Lane05	302,000	30.5	GFP	283,000	28.8	17,100	6.03	0	Sample
06	Lane06	255,000	25.7	GFP	283,000	28.8	17,100	6.03	0	Sample
07	Lane07	328,000	32.7	SLIT3-C	326,000	33.5	23,000	7.06	0	Sample
08	Lane08	349,000	35.7	SLIT3-C	326,000	33.5	23,000	7.06	0	Sample
09	Lane09	314,000	33.4	SLIT3-C	326,000	33.5	23,000	7.06	0	Sample
10	Lane10	357,000	37.4	SLIT3-C	326,000	33.5	23,000	7.06	0	Sample
11	Lane11	296,000	30.3	SLIT3-C	326,000	33.5	23,000	7.06	0	Sample
12	Lane12	315,000	31.8	SLIT3-C	326,000	33.5	23,000	7.06	0	Sample

Chemi Channel

Image Name: 0000725-TH in SLIT3-C OE

Lane Detection: Automatic Lane Finding

Protein: TH

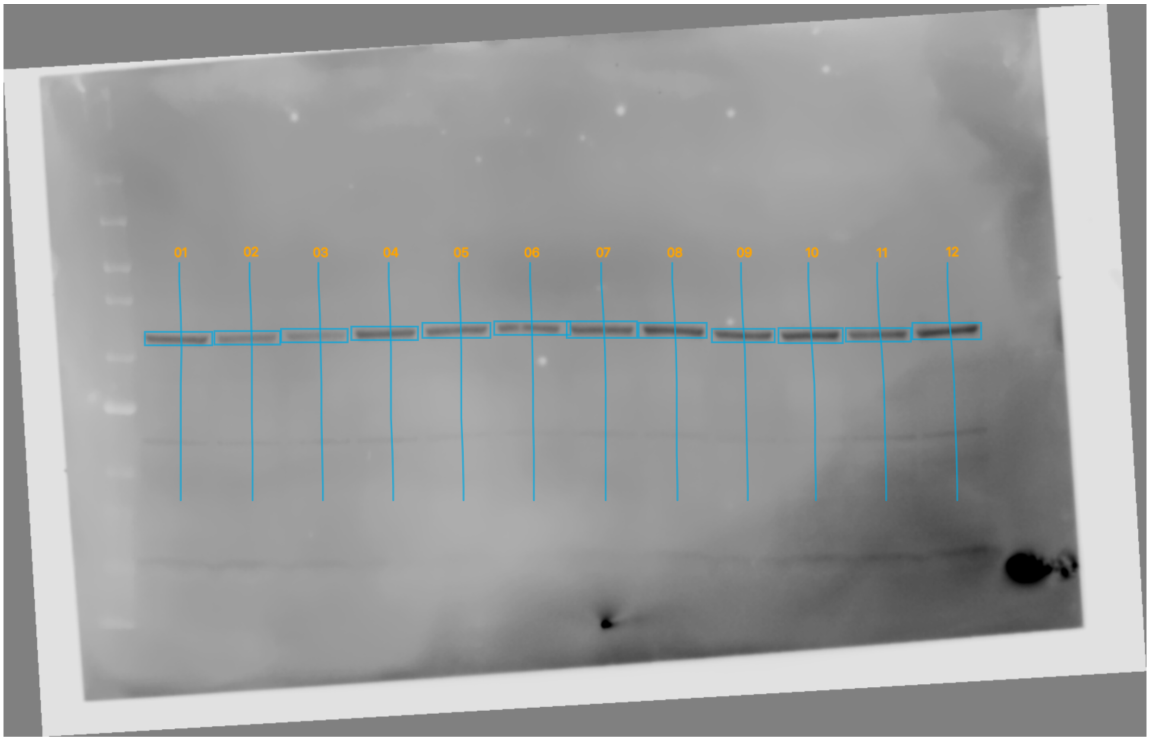

Protein Analysis Table

Lane	Name	MW	Normalized Signal	SNR	Replicate	Avg. Norm. Signal	Avg. SNR	Std. Dev.	% CV	Treatment (%)	Type
01	Lane01		2.21	5.20	GFP	1.93	3.84	0.578	29.9	0	Sample
02	Lane02		1.45	3.86	GFP	1.93	3.84	0.578	29.9	0	Sample
03	Lane03		1.11	2.99	GFP	1.93	3.84	0.578	29.9	0	Sample
04	Lane04		2.66	5.84	GFP	1.93	3.84	0.578	29.9	0	Sample
05	Lane05		2.32	2.96	GFP	1.93	3.84	0.578	29.9	0	Sample
06	Lane06		1.86	2.15	GFP	1.93	3.84	0.578	29.9	0	Sample
07	Lane07		2.30	3.74	SLIT3-C	2.71	9.07	0.297	11.0	0	Sample
08	Lane08		2.83	7.35	SLIT3-C	2.71	9.07	0.297	11.0	0	Sample
09	Lane09		2.50	13.4	SLIT3-C	2.71	9.07	0.297	11.0	0	Sample
10	Lane10		2.98	18.0	SLIT3-C	2.71	9.07	0.297	11.0	0	Sample
11	Lane11		2.59	9.05	SLIT3-C	2.71	9.07	0.297	11.0	0	Sample
12	Lane12		3.07	2.79	SLIT3-C	2.71	9.07	0.297	11.0	0	Sample

Figure 5o

Anti-UCP1

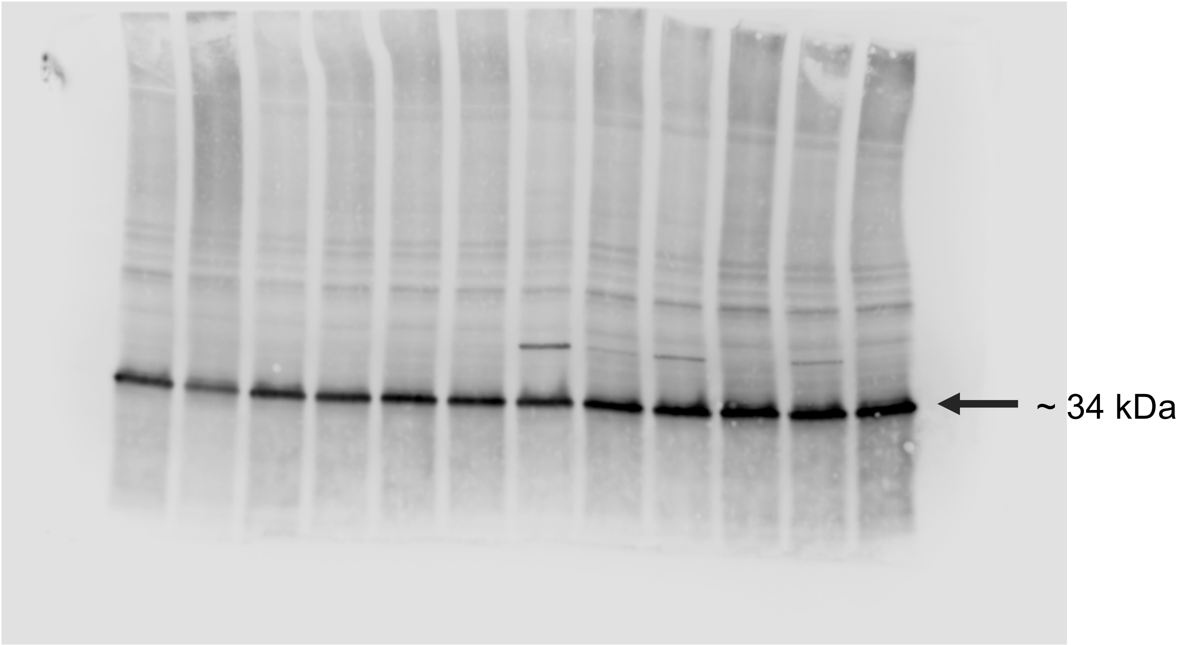

Total Protein

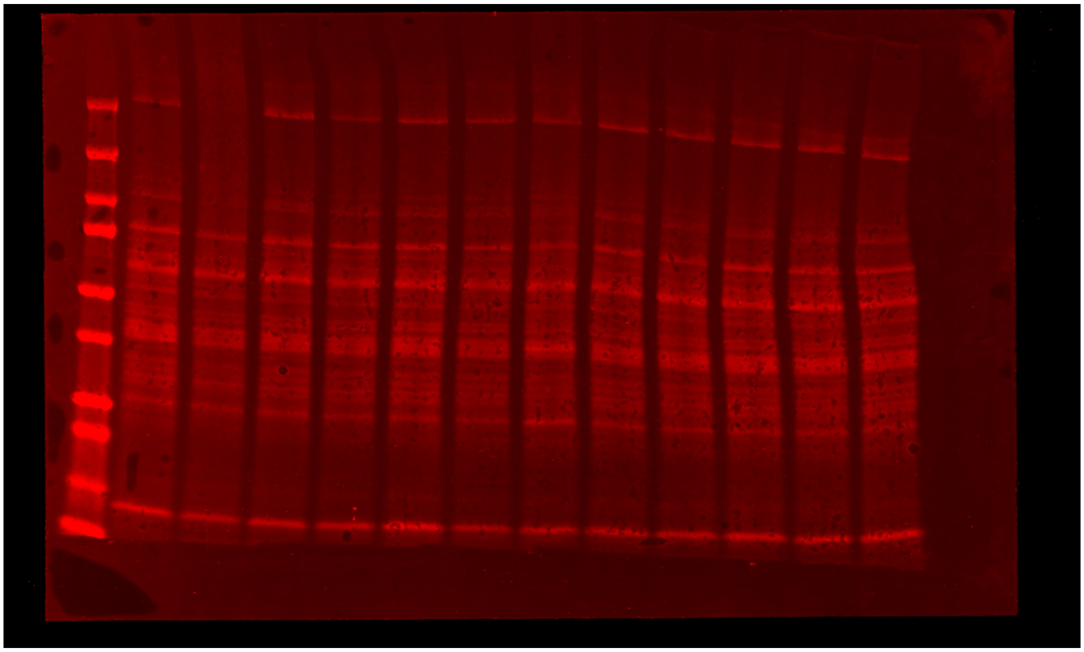

Experimental Analysis

700 Channel

Image Name: TotalproteinGFPxC

Lane Detection: Automatic Lane Finding

Protein: TOTAL PROTEIN

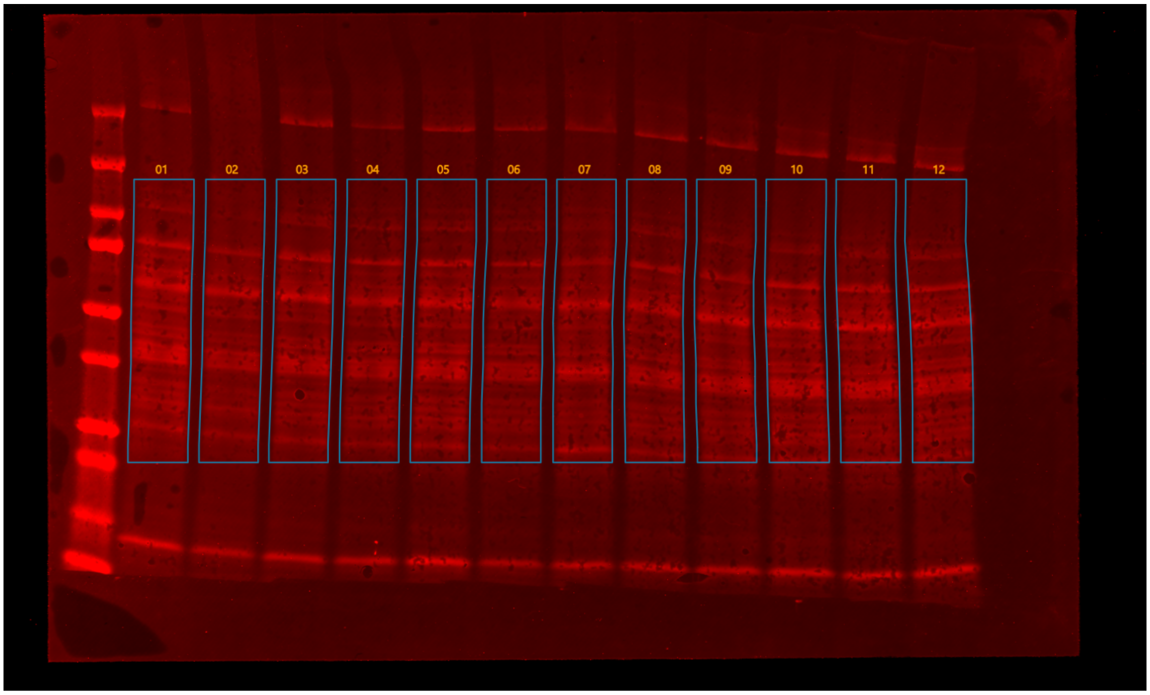

Total Protein Stain Analysis Table

Lane	Name	Signal	SNR	Replicate	Avg. Signal	Avg. SNR	Std. Dev.	% CV	Treatment (%)	Type
01	GFP	163,000	20.6	Rep-Grp-01-02-03-0...	113,000	14.4	17,400	15.4	0	Sample
02	GFP	117,000	15.0	Rep-Grp-01-02-03-0...	113,000	14.4	17,400	15.4	0	Sample
03	GFP	116,000	14.9	Rep-Grp-01-02-03-0...	113,000	14.4	17,400	15.4	0	Sample
04	GFP	99,600	12.8	Rep-Grp-01-02-03-0...	113,000	14.4	17,400	15.4	0	Sample
05	GFP	117,000	15.0	Rep-Grp-01-02-03-0...	113,000	14.4	17,400	15.4	0	Sample
06	GFP	94,100	12.1	Rep-Grp-01-02-03-0...	113,000	14.4	17,400	15.4	0	Sample
07	Slit3 C	107,000	13.7	Rep-Grp-01-02-03-0...	113,000	14.4	17,400	15.4	0	Sample
08	Slit3 C	103,000	13.2	Rep-Grp-01-02-03-0...	113,000	14.4	17,400	15.4	0	Sample
09	Slit3 C	101,000	13.0	Rep-Grp-01-02-03-0...	113,000	14.4	17,400	15.4	0	Sample
10	Slit3 C	114,000	14.5	Rep-Grp-01-02-03-0...	113,000	14.4	17,400	15.4	0	Sample
11	Slit3 C	112,000	14.2	Rep-Grp-01-02-03-0...	113,000	14.4	17,400	15.4	0	Sample
12	Slit3 C	113,000	14.1	Rep-Grp-01-02-03-0...	113,000	14.4	17,400	15.4	0	Sample

Chemi Channel

Image Name: ucp1 and THSlit3C

Lane Detection: Automatic Lane Finding

Protein: ucp1

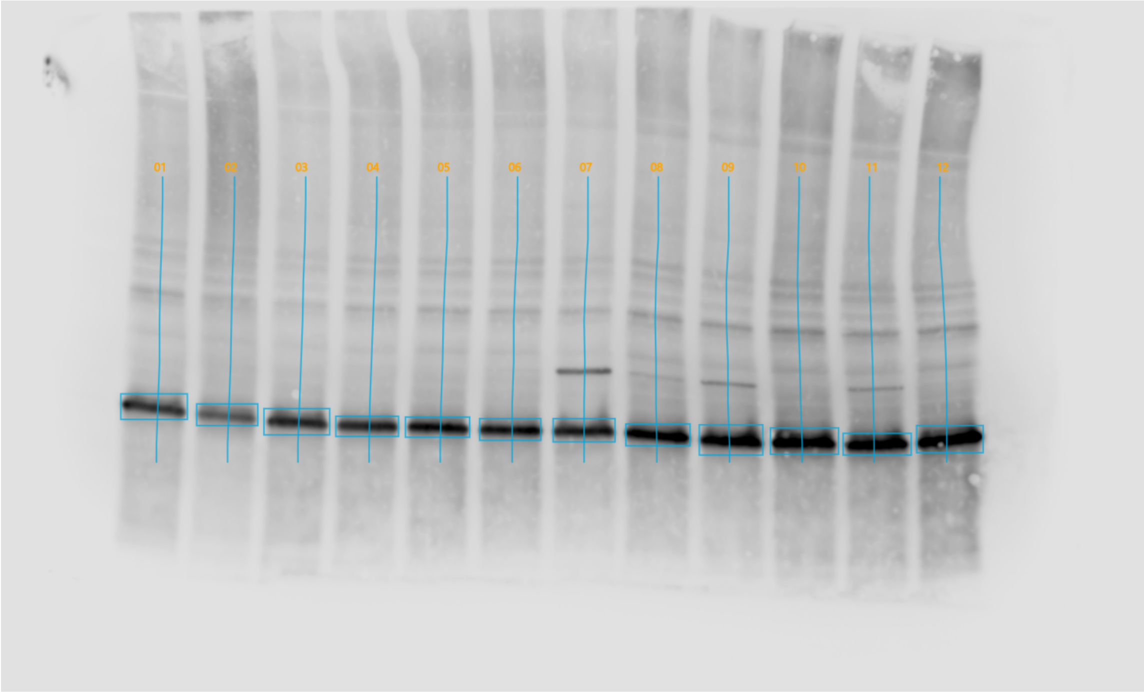

Protein Analysis Table

Lane	Name	MW	Normalized Signal	SNR	Replicate	Avg. Norm. Signal	Avg. SNR	Std. Dev.	% CV	Treatment (%)	Type
01	GFP		33.2	7.67	Rep-Grp-01-02-03...	54.2	6.80	15.2	28.1	0	Sample
02	GFP		29.3	5.13	Rep-Grp-01-02-03...	54.2	6.80	15.2	28.1	0	Sample
03	GFP		46.9	7.22	Rep-Grp-01-02-03...	54.2	6.80	15.2	28.1	0	Sample
04	GFP		47.2	8.52	Rep-Grp-01-02-03...	54.2	6.80	15.2	28.1	0	Sample
05	GFP		44.1	7.27	Rep-Grp-01-02-03...	54.2	6.80	15.2	28.1	0	Sample
06	GFP		54.8	8.59	Rep-Grp-01-02-03...	54.2	6.80	15.2	28.1	0	Sample
07	Slit3 C		49.8	7.20	Rep-Grp-01-02-03...	54.2	6.80	15.2	28.1	0	Sample
08	Slit3 C		61.4	6.83	Rep-Grp-01-02-03...	54.2	6.80	15.2	28.1	0	Sample
09	Slit3 C		76.6	5.52	Rep-Grp-01-02-03...	54.2	6.80	15.2	28.1	0	Sample
10	Slit3 C		70.3	5.52	Rep-Grp-01-02-03...	54.2	6.80	15.2	28.1	0	Sample
11	Slit3 C		62.7	6.57	Rep-Grp-01-02-03...	54.2	6.80	15.2	28.1	0	Sample
12	Slit3 C		73.7	5.56	Rep-Grp-01-02-03...	54.2	6.80	15.2	28.1	0	Sample

Figure 6d

Input
Anti-HaloTag

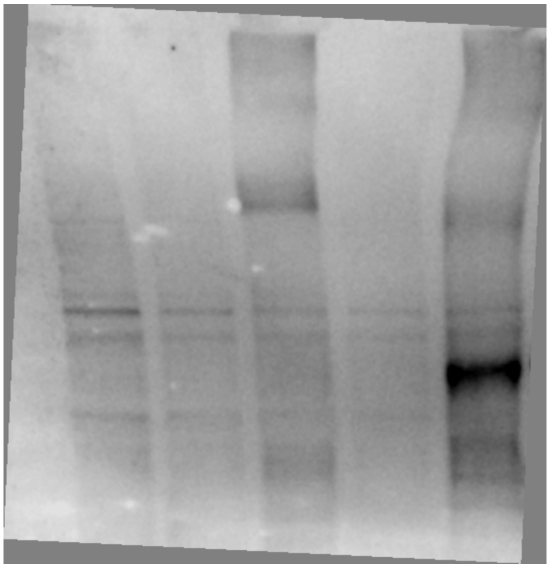

IP: His-tag
Anti-HaloTag

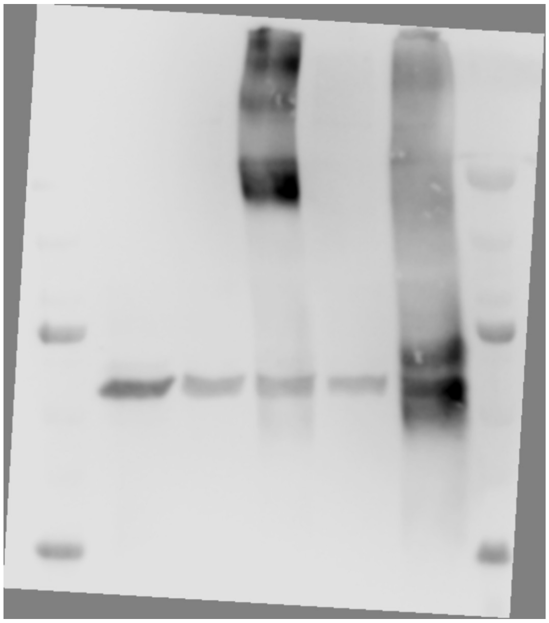

IP: His-tag
Anti-His-tag

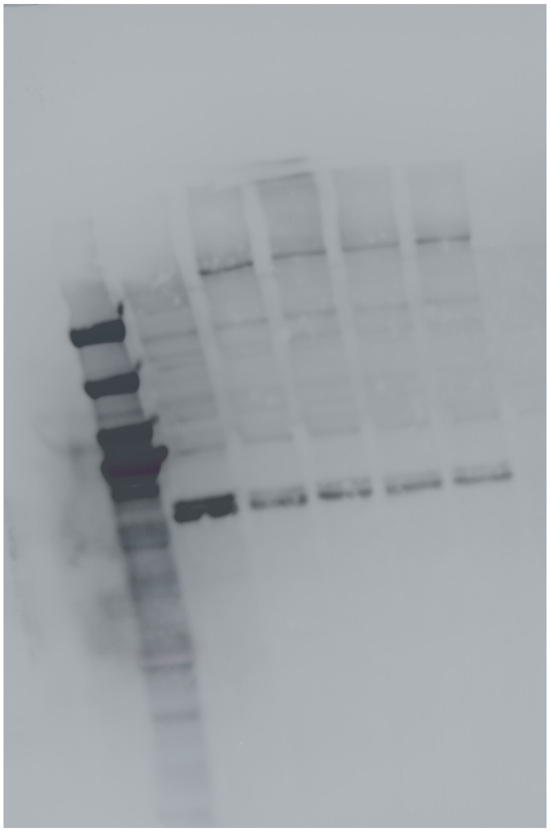

Supplement: Supplementary file 4 — Source data [file 41467_2026_70310_MOESM4_ESM.zip › Source Data 2.pdf]
